# The Role of Inflammation and Immunity in Cardiovascular Disease: Molecular Mechanisms and Therapeutic Targets

**DOI:** 10.1002/mco2.70878

**Published:** 2026-07-19

**Authors:** Jiaxiang Rong, Zhen Wang, Xiaoxiao Lin, Ziwen Lei, Qianqian Huang, Hang Liu, Fei Luan, Junbo Zou, Yajun Shi

**Affiliations:** ^1^ Shaanxi Province Key Laboratory of New Drugs and Chinese Medicine Foundation Research, School of Pharmacy Shaanxi University of Chinese Medicine Xi'an Shaanxi China; ^2^ Department of Pharmacy Affiliated Hospital of Shaanxi University of Chinese Medicine Xianyang China

**Keywords:** cardiovascular diseases, cGAS–STING pathway, immune network, inflammation, mitochondrial danger signaling, mitophagy

## Abstract

Cardiovascular inflammation is increasingly recognized not merely as a secondary response to hemodynamic or metabolic injury, but as a determinant of disease initiation, progression, and remodeling. In the injured heart and vasculature, danger sensing, inflammatory priming, inflammasome activation, nucleic acid recognition, immunometabolic rewiring, immunothrombosis, adaptive immune remodeling, and defective resolution form interdependent circuits rather than isolated pathways. A key challenge is to understand how these circuits shift from adaptive clearance and repair to persistent immune activation, fibrosis, and functional decline. Here, we synthesize evidence on immune landscapes and core inflammatory networks in cardiovascular diseases, focusing on TLR–NF‐κB signaling, NLRP3 inflammasomes, cGAS–STING‐dependent cytosolic DNA sensing, alternative mitochondrial nucleic acid‐sensing platforms, and redox‐immunometabolic gating. We highlight mitochondrial quality control and mitochondria‐derived DAMPs, including mtDNA, mtROS, ATP, cardiolipin, and oxidized lipids, as an upstream interface linking metabolic stress to sterile immune activation. We further organize disease‐specific inflammatory patterns through a stage‐cell‐threshold perspective across ischemic injury, vascular and metabolic disease, and cardiomyopathic remodeling. Finally, we discuss network‐guided therapeutic strategies, translational limitations, biomarkers, endpoints, and safety considerations. This integrated perspective provides a conceptual basis for moving cardiovascular inflammatory therapy from broad suppression toward more precise network regulation.

## Graphical abstract

Inflammation in cardiovascular disease is a dynamic and context‐dependent network rather than a secondary response to injury. Mitochondrial danger signaling links metabolic stress to immune activation through TLR priming, NLRP3 inflammasome amplification, cGAS–STING signaling, and immunometabolic gating, providing a basis for more precise therapeutic intervention.

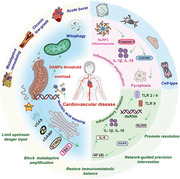

## 1 Introduction

Cardiovascular diseases (CVDs) remain among the leading causes of morbidity and mortality worldwide and continue to impose a substantial clinical and socioeconomic burden [1, 2]. Historically, these disorders have been interpreted mainly through the frameworks of hemodynamic overload, metabolic imbalance, ischemic injury, and structural remodeling [3, 4, 5]. With the continued development of cardiovascular immunology and inflammation biology, however, it has become increasingly clear that CVDs are not driven solely by mechanical or metabolic stress. Inflammatory disequilibrium and immune dysregulation also participate in disease initiation, progression, and clinical outcomes [6]. Under physiological conditions, the cardiovascular system maintains a regulated immune homeostasis that supports tissue surveillance, debris clearance, and adaptive repair without eliciting excessive inflammatory injury. Under pathological conditions, persistent oxidative stress, mitochondrial dysfunction, metabolic disturbance, and cellular injury disrupt this balance, leading to the accumulation of endogenous damage‐associated molecular patterns (DAMPs), sterile inflammation, maladaptive remodeling, and progressive functional decline [7]. Therefore, CVDs should be understood not merely as mechanical or metabolic disorders, but as conditions shaped by the continuous interaction between cardiometabolic stress and immune‐inflammatory regulation.

Over the past decade, substantial progress has been made in defining individual inflammatory mechanisms involved in cardiovascular pathology, including pattern‐recognition receptor (PRR) signaling, inflammasome activation, immunothrombosis, adaptive immune remodeling, and nucleic acid‐sensing pathways [8, 9]. Several recent reviews have also summarized cyclic GMP–AMP synthase (cGAS)‐stimulator of interferon genes (STING) signaling and selected inflammatory pathways in cardiovascular and noncardiovascular contexts [10, 11]. Nevertheless, the existing literature remains largely centered on individual signaling axes and does not fully explain how these pathways are organized into an interconnected and dynamically evolving inflammatory network in CVDs [7]. This limitation is important because cardiovascular inflammation is rarely determined by a single pathway in isolation. Instead, it is shaped jointly by multiple interacting layers, including danger signal sensing, inflammatory priming, signal amplification, metabolic adaptation, immune‐cell remodeling, and impaired resolution [12, 13, 14]. Thus, a more integrative perspective is needed to clarify how these inflammatory modules interact and why distinct inflammatory patterns emerge across different CVD settings.

Mitochondrial danger signaling provides an important entry point for such an integrated understanding. Mitochondrial dysfunction not only compromises bioenergetic homeostasis, but also promotes the release or exposure of multiple immunologically active molecules, including mitochondrial DNA (mtDNA), mitochondrial reactive oxygen species (mtROS), oxidized lipids, and cardiolipin (CL) [7, 15]. These mitochondria‐derived DAMPs can activate multiple innate immune platforms and link metabolic stress to sterile inflammation. Among these mechanisms, the mitophagy–mtDNA–cGAS–STING axis is particularly informative because it illustrates how impaired mitochondrial quality control (MQC) can convert metabolic disequilibrium into innate and adaptive immune activation [16, 17]. However, mitochondrial danger signaling should not be viewed as a single linear pathway. Its inflammatory consequences depend on disease stage, dominant cell type, signal intensity, mitochondrial reserve, and the capacity for inflammatory resolution. From this standpoint, cardiovascular inflammation is better understood as a dynamic, multilayered, and context‐dependent regulatory network rather than a parallel collection of isolated signaling pathways.

This review is organized to move progressively from the general immune landscape of CVDs to disease‐specific inflammatory organization and therapeutic translation. Details regarding the literature search strategy and scope of this review are provided in the Supporting Information. We first outline key components of the cardiovascular immune landscape, including danger signal sensing, inflammasome‐mediated amplification, immunothrombosis, adaptive immunity, and inflammatory resolution. We then discuss core inflammatory signaling networks, with emphasis on Toll‐like receptors (TLR)‐related initiation, NOD‐like receptor (NLR) family pyrin domain containing 3 (NLRP3)‐mediated amplification, cGAS–STING‐dependent nucleic acid sensing, alternative mitochondrial immune platforms, and immunometabolic gating. Next, we analyze how MQC and mitophagy regulate mitochondrial DAMP generation and inflammatory network activation. On this basis, representative CVD settings are organized through a stage‐cell‐threshold perspective to interpret acute high‐intensity inflammation in ischemic injury, chronic low‐grade inflammation in vascular and metabolic disease, and maladaptive inflammatory maintenance in stress overload and cardiomyopathy. Finally, we discuss network‐guided therapeutic strategies, preclinical and clinical evidence, unresolved mechanistic questions, and future translational priorities for more precise inflammatory and immune modulation in CVDs.

## 2 Immune Landscape in Cardiovascular Diseases

CVDs have long been regarded as disorders driven primarily by hemodynamic abnormalities, metabolic disturbances, and structural injury. However, accumulating evidence indicates that their initiation and progression are also shaped by dysregulated immune signaling [8, 18, 19]. Physiologically, the heart and vascular system maintain immune homeostasis that permits tissue surveillance, debris clearance, and adaptive remodeling without excessive inflammatory injury. This balance can be disrupted by persistent mechanical stress, metabolic burden, oxidative injury, and mitochondrial dysfunction, leading to the accumulation of endogenous DAMPs, including oxidized lipids, extracellular matrix fragments, reactive oxygen species (ROS), mtDNA, and CL [7, 20, 21, 22]. When DAMP generation exceeds the buffering capacity of local quality‐control and resolution systems, sterile inflammation is initiated and progressively reshapes the myocardial and vascular microenvironment. Therefore, elucidating the immune architecture underlying this transition provides a conceptual framework for integrating specific molecular axes into a broader inflammatory network.

### 2.1 Pattern‐Recognition Receptors in Cardiovascular Diseases

PRRs serve as the principal sensors for detecting endogenous danger signals in cardiovascular tissues. Unlike pathogen‐induced inflammation, sterile inflammation in the cardiovascular system is mainly triggered by DAMPs released during cellular stress, necrosis, oxidative injury, or mitochondrial dysfunction [7, 23].

TLRs represent a major PRR family involved in cardiovascular pathology. TLRs can be activated by oxidized lipids, extracellular matrix fragments, or nucleic acid debris, and subsequently transmit signals through myeloid differentiation primary response 88 (MyD88)‐dependent or TIR‐domain‐containing adaptor‐inducing interferon‐β (TRIF)‐dependent pathways. These signaling cascades ultimately converge on the activation of nuclear factor‐kappa B (NF‐κB) and mitogen‐activated protein kinase (MAPK) pathways. Activation of these axes drives the transcription of proinflammatory cytokines, chemokines, and adhesion molecules, thereby promoting leukocyte recruitment and endothelial activation [24, 25].

NLRs constitute another important class of PRRs. Among these receptors, NLRP3 is particularly important because it nucleates inflammasome assembly, activates caspase‐1, and promotes the maturation of interleukin‐1β (IL‐1β) and interleukin‐18 (IL‐18). Inflammasome activation has been closely associated with myocardial injury, progression of heart failure (HF), and atherosclerotic plaque destabilization [26].

In addition, cytosolic nucleic acid sensors recognize DNA or RNA that is aberrantly localized within the cytoplasm. Among these sensors, cGAS activates STING, thereby initiating innate immune signaling programs. Increasing evidence suggests that this pathway plays an important role in sterile cardiovascular inflammation, particularly when mitochondrial or nuclear DNA is aberrantly exposed to the cytosol.

Together, these PRR systems form an interconnected sensing network that converts endogenous stress signals into inflammatory activation.

### 2.2 Inflammasomes and Signal Amplification Circuits

Danger sensing alone is insufficient to sustain persistent inflammation. Instead, inflammatory amplification modules determine the intensity, duration, and pathological consequences of immune activation. Within cardiovascular inflammatory signaling, NF‐κB, the TANK‐binding kinase 1 (TBK1)–interferon regulatory factor 3 (IRF3) axis, and inflammasome‐related pathways serve as central hubs that convert initial danger sensing into sustained inflammatory responses.

The NF‐κB pathway is broadly involved in multiple cellular processes and serves as a central transcriptional integrator downstream of several PRRs, including TLRs and selected NLRs. Its activation induces the expression of proinflammatory cytokines, chemokines, and adhesion molecules, thereby promoting leukocyte infiltration and stromal cell activation. Persistent NF‐κB activation has been closely associated with chronic low‐grade inflammation and structural remodeling in HF and atherosclerosis.

TBK1, an IκB kinase (IKK)‐related kinase, consists of an N‐terminal kinase domain, a central ubiquitin‐like domain (ULD), and a C‐terminal elongated helix domain, which together support its kinase activity [27]. IRF3, a member of the interferon regulatory factor family, contains a DNA‐binding domain (DBD), a transcriptional activation domain (TAD), and a response domain (RD), arranged from the N‐terminus to the C‐terminus, and acts as a key transcription factor in immune responses [28]. The TBK1–IRF3 axis is mainly activated by cytosolic nucleic acid‐sensing pathways, such as cGAS–STING. This signaling axis promotes Type I interferon (IFN‐I) responses and the expression of interferon‐stimulated genes (ISGs), thereby linking intracellular danger signal recognition to broader programs of immune reprogramming [29].

The NLRP3 inflammasome represents another important amplification module that couples PRR‐derived priming signals to Caspase‐1‐dependent cytokine maturation and inflammatory cell death. Through the release of IL‐1β and the induction of pyroptosis, inflammasome activation can markedly intensify local inflammatory responses and promote fibrotic remodeling [26].

Taken together, these amplification pathways transform local danger sensing into sustained inflammatory cascades, thereby exerting profound effects on cardiovascular structure and function.

### 2.3 Immunothrombosis: Complement–Coagulation–Platelet Interplay

In CVDs, inflammatory signaling and the hemostatic system do not operate as independent processes but instead form a tightly coupled response network. Although the complement system has traditionally been recognized as a key component of host defense against pathogens, it also plays a critical regulatory role in sterile cardiovascular inflammation. Complement activation not only increases endothelial permeability and promotes leukocyte recruitment, but also influences fibrin formation and thrombus stability through its interactions with the coagulation cascade and platelet activation [8, 30]. Meanwhile, activated platelets release chemokines and upregulate multiple surface adhesion and immunoregulatory molecules, thereby further amplifying inflammatory signaling and promoting the accumulation of immune cells at sites of vascular injury. This reciprocal amplification among complement components, coagulation factors, platelets, and immune cells constitutes the central basis of immunothrombosis.

This process is particularly relevant to plaque rupture, microvascular obstruction, and ischemia–reperfusion injury. Moderate activation may help contain tissue damage, whereas excessive or sustained activation exacerbates ischemic injury and drives adverse cardiac and vascular remodeling. Thus, the interplay between immune and hemostatic systems represents a major mechanistic interface through which inflammation contributes to acute cardiovascular events.

### 2.4 Adaptive Immunity and Resolution Mechanisms

Although innate immune signaling predominates during the early phase of sterile cardiovascular inflammation, adaptive immunity also contributes substantially to chronic remodeling and disease progression. Distinct T cell subsets regulate cytokine profiles, fibroblast activation, extracellular matrix deposition, and tissue repair. Proinflammatory effector T cells may aggravate myocardial injury, whereas regulatory T cells help restrain excessive inflammation and support reparative remodeling [31, 32]. In specific cardiovascular pathological contexts, B cells and autoantibody responses may also influence myocardial contractile function, vascular integrity, and chronic immune activation. Notably, adaptive immunity can either amplify inflammatory injury or promote its resolution, depending on the pattern of antigen exposure, cellular composition, metabolic state, and local cytokine milieu [7]. Persistent antigenic stimulation, metabolic stress, or mitochondria‐derived signals may sustain adaptive immune activation, thereby contributing to the chronic low‐grade inflammatory state observed in HF and advanced atherosclerosis [12].

Resolution mechanisms are equally important in determining the outcome of cardiovascular inflammation. Efficient clearance of cellular debris, restoration of mitochondrial integrity, metabolic rebalancing, macrophage phenotype transition, regulatory T cell activity, and specialized proresolving mediators collectively promote the shift from inflammatory activation toward tissue repair [7, 13, 33]. By contrast, impaired resolution allows inflammatory signals to persist, thereby promoting fibrosis, vascular dysfunction, and progressive functional decline [13]. Therefore, adaptive immune remodeling and inflammatory resolution should be viewed as interconnected processes rather than separate events. Together, they determine whether cardiovascular inflammation remains a controlled reparative response or evolves into chronic pathological injury.

Taken together, the immune landscape of CVDs consists of several coordinated layers, including danger sensing, inflammatory amplification, immunothrombotic coupling, adaptive immune remodeling, and inflammatory resolution. These layers do not operate independently but interact through shared upstream danger signals, overlapping transcriptional programs, and reciprocal communication between immune cells and cardiovascular structural cells. Within this multilayered network, mitochondria‐derived signals and nucleic acid‐sensing pathways occupy central positions by tightly linking metabolic stress to innate immune activation. Thus, cardiovascular inflammatory phenotypes are not determined by isolated pathway abnormalities, but instead reflect the dynamic imbalance of interconnected immune modules across different pathological contexts.

## 3 Core Inflammatory Signaling Networks in Cardiovascular Diseases

**FIGURE 1:**
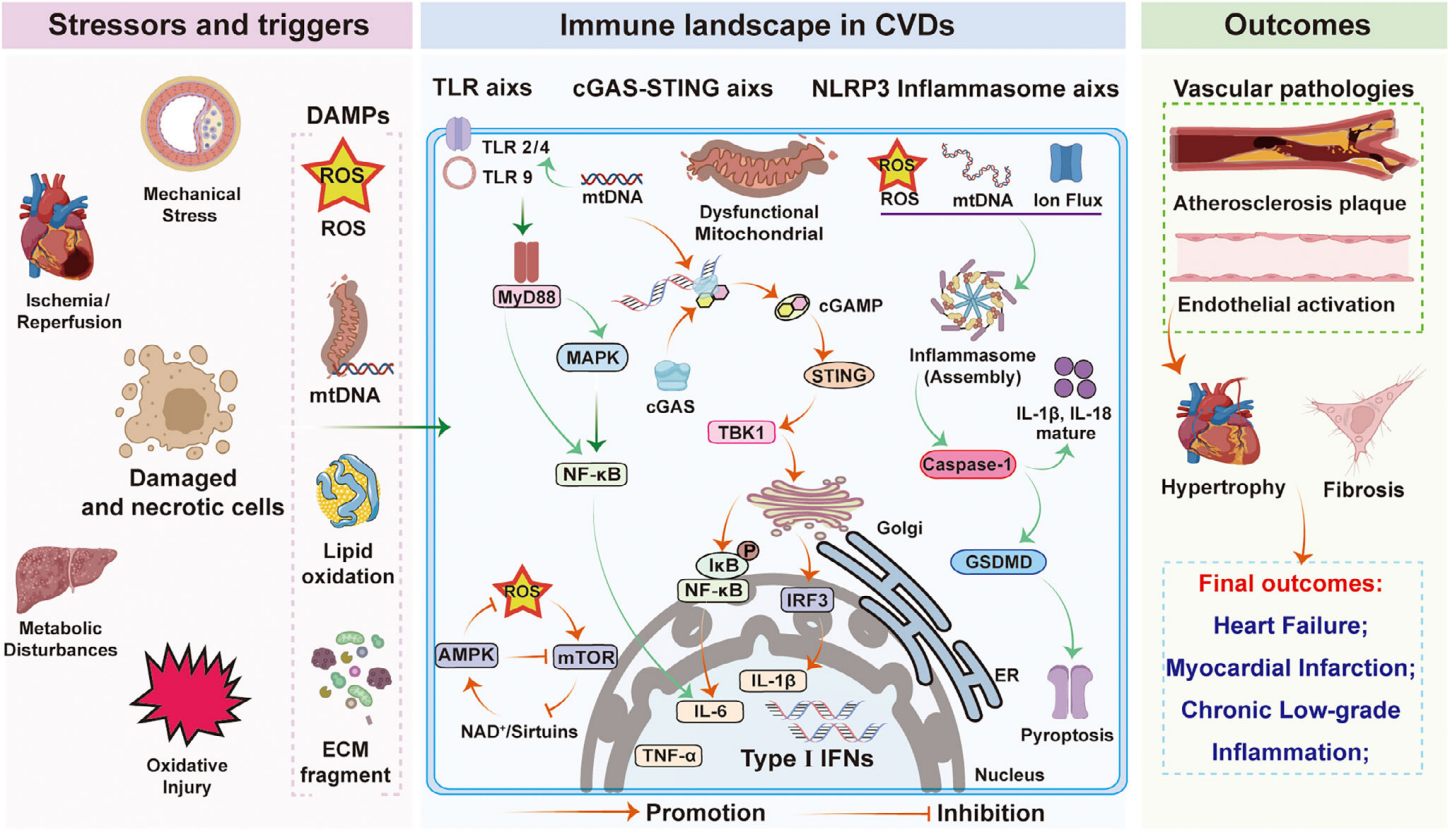
Immune landscape and representative inflammatory signaling networks in cardiovascular diseases. Pathological stressors trigger the release of DAMPs, which activate the TLR, cGAS–STING, and NLRP3 inflammasome axes in cardiovascular tissues. These interconnected pathways drive inflammatory priming, Type I interferon and cytokine responses, caspase‐1 activation, and pyroptosis, while being shaped by immunometabolic regulation. Collectively, they contribute to endothelial activation, plaque formation, hypertrophy, fibrosis, and adverse cardiovascular outcomes.

The immune architecture outlined above ultimately converges on several core signaling networks that determine the initiation, amplification, persistence, and context‐specific features of inflammatory responses in CVDs. Rather than acting as isolated pathways, these networks integrate danger sensing, transcriptional activation, inflammasome assembly, nucleic acid recognition, redox stress, and metabolic adaptation, with an integrated overview provided in Figure 1. Among them, TLR signaling mainly provides inflammatory initiation and priming, the NLRP3 inflammasome amplifies inflammatory execution, cGAS–STING signaling links aberrant cytosolic DNA sensing to innate immune activation, and immunometabolic regulation shapes the threshold and duration of inflammatory responses. Understanding these core modules and their interactions provides a mechanistic basis for interpreting the heterogeneous inflammatory phenotypes observed across different cardiovascular settings.

### 3.1 TLR Signaling in Inflammatory Initiation and Inflammasome Priming

TLRs constitute one of the principal sensing systems through which sterile danger signals initiate inflammatory responses in CVDs. In addition to recognizing pathogen‐associated molecular patterns, several members of the TLR family, including TLR2, TLR4, and TLR9, can detect endogenous DAMPs released from injured or stressed cardiovascular tissues, such as oxidized lipids, extracellular matrix fragments, heat shock proteins, and mtDNA [7, 34]. Upon ligand binding, TLRs recruit adaptor proteins and activate two major downstream branches, namely, the MyD88‐dependent pathway and the TRIF‐dependent pathway. These signaling cascades ultimately converge on transcriptional programs that regulate inflammatory gene expression [25, 34].

Activation of the MyD88‐dependent pathway rapidly engages NF‐κB and MAPK signaling, thereby inducing the transcription of proinflammatory cytokines, including tumor necrosis factor‐α (TNF‐α) and interleukin‐6 (IL‐6) [25, 34]. In parallel, the TRIF‐dependent pathway activates interferon‐related transcriptional programs and contributes to broader innate immune activation [25]. Together, these outputs reshape the inflammatory state of the cardiovascular microenvironment. In endothelial cells, TLR activation promotes the expression of adhesion molecules such as intercellular adhesion molecule‐1 (ICAM‐1) and vascular cell adhesion molecule‐1 (VCAM‐1), as well as chemokines that facilitate leukocyte adhesion and transendothelial migration. In cardiomyocytes and stromal cells, sustained TLR signaling can promote cytokine‐driven hypertrophic responses, fibroblast activation, and interstitial fibrosis [35].

Beyond directly inducing inflammatory gene expression, TLR signaling also provides the priming signal required for inflammasome activation. Through NF‐κB‐dependent transcription, TLR activation upregulates NLRP3 and the precursor forms of IL‐1β and IL‐18, thereby licensing cells for subsequent inflammasome assembly [36]. This process constitutes the first stage of the classical two‐signal model of inflammasome activation, in which transcriptional priming precedes the second activation signal triggered by cellular stress. Accordingly, the TLR–NF‐κB axis functions not only as an initiating mechanism for inflammatory activation, but also as a critical upstream platform that determines the responsiveness of downstream amplification modules. Sustained activation of this axis may therefore contribute to the chronic low‐grade inflammation observed in advanced atherosclerosis and HF.

### 3.2 NLRP3 Inflammasome in Inflammatory Amplification

The NLRP3 inflammasome is a major amplification module that links cellular stress to inflammatory cytokine maturation and inflammatory cell death, thereby playing a central role in CVDs. Its activation generally follows the classical two‐signal model. The first step is the priming signal, which is most commonly mediated by NF‐κB‐dependent transcription downstream of PRRs, such as TLR signaling. This priming phase induces the expression of NLRP3 itself as well as the precursor forms of IL‐1β and IL‐18, thereby licensing cells for subsequent inflammasome assembly [37].

The second step is the activation signal, which is triggered by multiple forms of cellular stress. Changes in ion flux, mitochondrial dysfunction, and the accumulation of ROS are among the most representative stimuli, and these signals can induce NLRP3 oligomerization [38]. Activated NLRP3 subsequently recruits apoptosis‐associated speck‐like protein containing a caspase recruitment domain (ASC) and procaspase‐1 to form a multiprotein inflammasome complex. Assembly of this complex promotes the activation of caspase‐1, which in turn cleaves pro‐IL‐1β and pro‐IL‐18 to generate their biologically active mature forms.

Activated caspase‐1 also cleaves gasdermin D, releasing its pore‐forming N‐terminal fragment and thereby inducing pyroptosis, a lytic form of inflammatory cell death. Membrane rupture during pyroptosis leads to the release of inflammatory mediators and additional danger signals, further amplifying local immune responses [39]. In myocardial tissue, this cascade has been closely associated with infarct expansion after ischemic injury, adverse cardiac remodeling, and contractile dysfunction. In the vascular wall, NLRP3 activation contributes to endothelial dysfunction, macrophage activation, and atherosclerotic plaque instability [26, 40].

Mitochondrial dysfunction represents an important upstream source of NLRP3‐activating signals. Excessive mitochondrial ROS production, altered mitochondrial membrane permeability, and the cytosolic release of mtDNA can all enhance inflammasome activation [37, 38]. These findings support the concept that mitochondria are not merely metabolic organelles, but also critical regulators of inflammatory amplification. By generating and releasing mitochondria‐derived danger signals, mitochondrial stress links metabolic disturbance to innate immune activation and helps determine the threshold for NLRP3 inflammasome activation in CVDs.

### 3.3 cGAS–STING Pathway in Cytosolic DNA Sensing

The cGAS–STING pathway functions as a central cytosolic DNA‐sensing system that converts aberrant DNA exposure into innate immune activation. Under physiological conditions, genomic DNA is confined to the nucleus, whereas mtDNA remains sequestered within mitochondria [29]. Therefore, the appearance of DNA in the cytosol represents a spatial danger signal associated with infection, cellular stress, organelle damage, or cell death. In CVDs, cytosolic DNA is largely derived from endogenous sources, particularly mtDNA released during mitochondrial dysfunction, oxidative stress, ischemia–reperfusion injury, metabolic disturbance, or cardiomyocyte death [10].

cGAS serves as the primary cytosolic DNA sensor in this pathway. Upon binding to dsDNA, cGAS undergoes conformational activation and catalyzes the synthesis of 2′3′‐cyclic GMP–AMP (cGAMP) from adenosine triphosphate (ATP) and guanosine triphosphate (GTP). cGAMP then binds to STING, an ER‐localized adaptor protein, inducing STING oligomerization and conformational rearrangement. One of the key events following STING activation is its trafficking from the ER to the ERGIC and Golgi apparatus, where STING assembles downstream signaling complexes and recruits TBK1 [20, 41, 42]. In sterile cardiovascular injury, mitochondrial damage and altered membrane permeability can promote mtDNA leakage into the cytosol, thereby providing an endogenous trigger for cGAS activation and subsequent STING‐dependent inflammatory signaling [43].

After STING activation and trafficking, TBK1 is recruited to the cytosolic C‐terminal region of STING and becomes activated through phosphorylation. Activated TBK1 subsequently phosphorylates IRF3, leading to IRF3 dimerization and nuclear translocation. Once in the nucleus, IRF3 drives the transcription of IFN‐I and ISGs, thereby establishing an antiviral‐like transcriptional program in response to cytosolic DNA exposure [28]. In CVDs, this response may be particularly relevant when mitochondrial injury or nuclear DNA damage generates persistent intracellular nucleic acid stress.

In parallel with the TBK1–IRF3 axis, activated STING can also engage NF‐κB‐dependent inflammatory transcriptional programs [44]. This branch may be activated through TBK1‐dependent or TBK1‐independent mechanisms and ultimately converges on IKK complex activation and NF‐κB nuclear translocation. Once activated, NF‐κB promotes the transcription of multiple proinflammatory mediators, including TNF‐α, IL‐6, IL‐1β, and chemokines [41, 45, 46]. The NF‐κB branch provides a rapid inflammatory output that complements the IFN‐I program and links DNA sensing to leukocyte recruitment, cytokine production, and local inflammatory amplification. Moreover, STING‐dependent NF‐κB activation can interact with TLR signaling and inflammasome‐related pathways, further embedding cGAS–STING within the broader inflammatory network of CVDs [47, 48].

In cardiovascular pathology, the functional consequences of cGAS–STING activation are highly context dependent. Transient activation after acute injury may contribute to damage recognition, clearance of injured cells, and initiation of reparative responses. By contrast, sustained activation driven by chronic mitochondrial dysfunction, persistent oxidative stress, or continuous DNA leakage can promote prolonged inflammatory signaling, immune‐cell infiltration, endothelial dysfunction, fibrosis, and adverse cardiac remodeling [28, 49]. Thus, cGAS–STING should not be regarded as a uniformly detrimental pathway, but rather as a cytosolic DNA‐sensing module whose effects depend on disease stage, cellular context, activation intensity, and duration.

Taken together, the cGAS–STING pathway constitutes a core module that translates the aberrant cytosolic localization of DNA into innate immune activation and occupies a central position in linking mitochondrial injury, nucleic acid sensing, and sterile inflammatory amplification. However, it does not represent the sole route through which mitochondrial danger signals are conveyed, and its activation threshold as well as pathological consequences are jointly shaped by other nucleic acid‐sensing platforms and the surrounding state of metabolic stress.

### 3.4 Immunometabolic and Redox Regulation of Inflammatory Signaling

In CVDs, inflammatory signaling is tightly coupled to cellular metabolic and redox states. Rather than serving merely as background conditions, immunometabolic and redox pathways shape the threshold, amplitude, and duration of immune activation, thereby functioning as network gain modulators within the broader inflammatory system [7, 12]. This regulatory layer is particularly important because the same inflammatory pathway may produce distinct outcomes depending on mitochondrial function, oxidative stress burden, nutrient availability, and cellular energy status.

ROS represent a central component of this regulation. Under conditions of metabolic stress, dysfunctional mitochondria become a major source of excessive ROS production. Although ROS directly contribute to macromolecular damage and mitochondrial injury, they also act as signaling mediators that amplify inflammatory pathways [50]. In particular, ROS can enhance NF‐κB‐dependent transcriptional programs and facilitate NLRP3 inflammasome activation, thereby linking oxidative stress to both inflammatory priming and downstream amplification [38]. Through these dual roles, ROS function not only as markers of cellular injury but also as active determinants of inflammatory escalation in cardiovascular tissues.

Another important regulatory axis involves nicotinamide adenine dinucleotide (NAD^+^)‐dependent deacetylases, especially sirtuins, which coordinate mitochondrial biogenesis, antioxidant defense, and inflammatory gene expression. Adequate NAD^+^ availability supports sirtuin activity and helps maintain mitochondrial homeostasis, whereas NAD^+^ depletion or impaired sirtuin function can promote oxidative stress, metabolic inflexibility, and inflammatory activation [51]. In settings such as metabolic disorders and HF, disruption of the NAD^+^–sirtuin axis may therefore shift the cellular environment toward persistent inflammation and impaired stress adaptation.

Energy‐sensing pathways centered on AMP‐activated protein kinase (AMPK) and mechanistic target of rapamycin (mTOR) provide an additional layer of immunometabolic control. AMPK generally promotes catabolic adaptation, autophagy, and MQC, thereby restraining excessive inflammatory activation. By contrast, mTOR signaling favors anabolic activity and can support proinflammatory immune programs under nutrient‐replete or stress‐associated conditions [52]. The balance between AMPK and mTOR therefore influences whether cells maintain a homeostatic state or shift toward sustained inflammatory activation. Importantly, these metabolic nodes also interact with nucleic acid‐sensing pathways, indicating that cellular metabolic status can determine the activation threshold and persistence of mitochondrial danger signaling.

Together, ROS, the NAD^+^–sirtuin axis, and AMPK/mTOR signaling form a metabolic and redox gating layer that regulates inflammatory network behavior in CVDs. By shaping danger signal generation, transcriptional responsiveness, inflammasome activation, and nucleic acid‐sensing persistence, immunometabolic regulation critically influences whether cardiovascular inflammation remains adaptive and controllable or progresses toward pathological amplification and remodeling.

### 3.5 Integration and Crosstalk Among Major Inflammatory Pathways

Although TLR signaling, NLRP3 inflammasome activation, cGAS–STING signaling, and immunometabolic regulation are discussed above as distinct modules, they rarely operate independently in CVDs [26, 53, 54]. Instead, these pathways are linked by shared upstream danger signals, overlapping transcriptional outputs, and reciprocal feedback between mitochondrial stress and inflammatory activation. Mitochondria‐derived signals are particularly important in this network because mtROS, mtDNA, oxidized lipids, and other DAMPs can simultaneously provide input for inflammatory priming, inflammasome activation, and nucleic acid‐sensing pathways [20].

Within this integrated network, TLR–NF‐κB signaling mainly establishes the primed inflammatory state, NLRP3 converts cellular stress into inflammatory amplification and pyroptotic execution, and cGAS–STING links aberrant cytosolic DNA exposure to IFN‐I and NF‐κB‐related outputs. These pathways can reinforce one another rather than function as isolated linear cascades. For example, persistent inflammatory activation can aggravate mitochondrial dysfunction and DNA damage, thereby increasing the availability of mitochondria‐derived ligands and further sustaining downstream immune activation [55].

Immunometabolic and redox regulation further determines the sensitivity and persistence of this inflammatory network. ROS, NAD^+^–sirtuin signaling, AMPK/mTOR balance, and MQC influence both danger signal generation and the activation threshold of inflammatory pathways [56]. Thus, the pathological consequence of cardiovascular inflammation is not determined simply by the activation of a single pathway, but by the coordinated state of danger signal input, inflammatory priming, signal amplification, nucleic acid sensing, and metabolic gating.

To further summarize the functional stratification of core inflammatory signaling modules in CVDs, Table 1 compiles representative evidence for TLR‐associated inflammatory initiation, NLRP3 inflammasome‐mediated amplification, cGAS–STING‐driven cytosolic DNA sensing, alternative mitochondrial nucleic acid sensing, and immunometabolic regulation across different cardiovascular settings. This table provides an evidence‐based overview of the upstream triggers, principal downstream outputs, and cardiovascular pathological relevance of the major signaling modules discussed in this section.

**TABLE 1:** Core inflammatory signaling axes and representative evidence in cardiovascular disease.

Disease	Experimental model	Inflammatory signaling	Representative trigger	Downstream output	Cardiovascular outcome	Refs.
**TLR‐related inflammatory initiation and priming**						
MI	**In vivo**: C57BL/6J mice, permanent ligation of the left coronary artery, *n* = 6/group **In vitro**: Primary cardiac fibroblasts and TLR9 reporter cells	TLR9–MyD88–NF‐κB axis	mtDNA and endogenous DNA released after MI	Unc93b1, DHX9, TLR9, MyD88 and NF‐κB ↑	Sterile inflammation and adverse post‐MI remodeling	[57]
MI/RI	**In vivo**: SD rats, left coronary artery occlusion 30 min and reperfusion	TLR9–CXCR2–neutrophil inflammatory axis	Short‐term pharmacological TLR9 inhibition by ODN 2088 at reperfusion	Neutrophil infiltration ↑, CXCR2 expression ↑, MPO, CitH4, and troponin T ↑	LV wall thinning and adverse remodeling	[58]
AS	**In vivo**: ApoE^−/−^ and Prdx5^−/−^, ApoE^−/−^ mice, normal chow diet for 60 weeks, *n* = 8/group **In vitro**: HUVECs stimulated with ox‐LDL	TLR4–MyD88–NF‐κB–P38	Extracellular PRDX5 release, ox‐LDL stimulation	Adhesion molecules and inflammatory cytokines ↑, monocyte/macrophage recruitment ↑	Endothelial inflammation and dysfunction aggravate atherosclerotic plaque formation	[59]
OCM	**In vivo**: C57BL/6 mice and TLR2 knockout mice, HFD 24 weeks, *n* = 6/group **In vitro**: H9c2 cells and primary neonatal rat cardiomyocytes, PA stimulation	TLR2–MyD88–NF‐κB signaling	HFD; palmitate; TLR2–MyD88 complex formation	IL‐1β, IL‐6 and TNF‐α ↑; macrophage infiltration ↑, fibrosis and hypertrophy markers ↑	Myocardial inflammation, fibrosis, hypertrophy, and cardiac dysfunction	[60]
**NLRP3 inflammasome and inflammatory amplification**						
CH	**In vivo**: Nlrp3^−/−^, P2rx7^−/−^ and Slc17a9 conditional knockout C57BL/6 mice, TAC‐induced pressure overload **In vitro**: Neonatal rat cardiomyocytes and fibroblasts, human cardiac microvascular endothelial cells	ATP‐P2 × 7‐NLRP3 inflammasome	Pressure overload‐induced extracellular ATP release	caspase‐1 activation, IL‐1β ↑, inflammatory gene expression ↑	Cardiac inflammation, hypertrophy, remodeling	[61]
AS	**In vivo**: Ldlr^−/−^ chimeric mice with 10% Tet2‐deficient bone marrow plus 90% WT bone marrow, fed WTD **In vitro**: Murine Tet2‐deficient BMDMs; human TET2^−/−^ hESC‐derived macrophages, with acLDL/ox‐LDL loading	JNK1/BRCC3/NLRP3 inflammasome	TET2 deficiency, cholesterol loading	IL‐1β ↑, caspase‐1 ↑, GSDMD cleavage ↑, NETosis ↑	Plaque burden ↑, plaque stability ↓	[62]
CH	**In vivo**: SD rats, TAC‐induced pressure overload, *n* = 6/group	Mitophagy dysfunction‐associated NLRP3 inflammasome activation	Pressure overload; impaired mitophagy	NLRP3, caspase‐1 and IL‐18 ↑, inflammatory cell infiltration ↑, ANP and MYH7 ↑	Cardiac hypertrophy	[63]
**cGAS–STING and cytosolic DNA sensing**						
CTX	**In vivo**: Acute and chronic doxorubicin treated WT and Zbp1^−/−^ mice (*n* = 12/group), Ifnar^−/−^ and Sting^−/−^ mice (*n* = 6/group) **In vitro**: MEFs, ARPE‐19, hiPSC‐CMs, and neonatal murine CMs	ZBP1–cGAS complex recruits RIPK1/RIPK3 and sustains STAT1 phosphorylation	mtDNA instability, Z‐DNA accumulation, cytosolic mtDNA stress	IFN‐I/ISGs ↑, STAT1 phosphorylation ↑, OXPHOS ↓	Cardiac dysfunction, fibrosis, vacuolization, cardiomyopathy	[64]
CTX	**In vivo**: Doxorubicin‐induced cardiotoxicity in cGAS^−/−^, Sting^−/−^, Irf3^−/−^ and STING‐ECKO mice **In vitro**: Doxorubicin‐treated HCMECs	cGAS–STING–TBK1–IRF3–CD38 axis	Doxorubicin‐induced nuclear and mitochondrial DNA damage, with cytosolic dsDNA release activating cGAS–STING	Inflammatory activation, CD38 ↑, NAD ↓, mitochondrial dysfunction, endothelial injury	Cardiac dysfunction and microvascular impairment	[65]
MI	**In vivo**: Permanent ligation in MT, Sting^gt/gt^, Cgas^−/−^, Mavs^−/−^, Ifnar^−/−^ and TRIF^Lps2/Lps2^ mice **In vitro**: Bone marrow‐derived macrophages stimulated with cardiomyocyte‐derived DNA	cGAS–STING–IRF3–Type I IFN/IFNAR axis	Ischemic cardiomyocyte death, Self‐derived‐dsDNA release	Ifnb1 ↑, Cxcl10 ↑, ISGs ↑; inflammatory cell infiltration ↑	Ventricular dysfunction ↑, rupture ↑, mortality ↑	[66]
MI	***Human cohort***: Clinical retrospective study, 270 patients with CHD	cGAS‐cGAMP‐STING, with TBK1–IRF3 and NF‐κB involvement	Aberrant plasma cGAMP level	Inflammatory cytokine signaling and MI susceptibility	Higher MI risk; U‐shaped association between cGAMP and MI	[67]
MI/RI	**In vivo**: C57BL/6 mice with HFD/STZ‐induced diabetes subjected to LAD ligation‐induced MI/RI, *n* = 6/group **In vitro**: H9c2 cells exposed to HG/PA and H/R	cGAS–STING‐TBK1–IRF3	MFN2 ↓, impaired mitochondrial fusion, cytosolic mtDNA release	p‐TBK1 ↑, p‐IRF3 ↑, NLRP3 ↑, TNF‐α ↑, IL‐1β ↑	Myocardial injury ↑, infarct size ↑, oxidative damage ↑, LVEF/LVFS ↓	[68]
MI/RI	**In vivo**: C57BL/6J mice, LAD ligation/reperfusion, *n* = 6/group **In vitro**: H/R cardiomyocytes and cardiac fibroblasts	cGAS–STING signaling	Ambra1^+^ cardiomyocyte‐derived sEVs carrying mtDNA	Fibroblast activation; fibrosis markers ↑; Inflammatory cytokines ↑	Cardiac fibrosis and dysfunction	[69]
HF	**In vivo**: TAC‐induced HF in C57BL/6J mice	cGAS–STING signaling	Cytosolic DNA accumulation	Inflammatory cell infiltration, TNF‐α ↑, IL‐1β ↑, IL‐18 ↑	Hypertrophy, fibrosis, apoptosis, cardiac dysfunction	[70]
AS	**In vivo**: ApoE^−/−^ and Ldlr^−/−^ C57BL/J mice, HFD for 12 weeks, *n* = 10/group **In vitro**: HUVECs treated with PA	IQGAP1–cGAS–STING–NLRP3	Lipotoxicity, oxidative stress, mtDNA release	GSDMD ↑, IL‐1β, IL‐18, pyroptosis ↑	Endothelial dysfunction and plaque progression	[71]
AS	**In vivo**: ApoE^−/−^ chimeric mice with ALDH2‐deficient bone marrow and HFD, *n* = 6/group **In vitro**: human PBMC‐derived macrophages, WT/ALDH2‐KO BMDMs, RAW264.7 cells	cGAS–STING‐TBK1/IRF3 signaling	4‐HNE accumulation, ALDH2 deficiency	Inflammatory cytokines ↑, M1‐like polarization ↑, M2‐like polarization ↓	Atherosclerotic plaque burden ↑, lesion progression aggravated	[72]
DCM	**In vivo**: C57BL/6J mice, HFD and STZ‐induced diabetes, myocardium‐specific STING knockdown by AAV9, *n* = 10/group **In vitro**: H9c2 cells and neonatal mouse cardiomyocytes treated with PA	mtDNA–cGAS–STING–TBK1–IRF3–NLRP3/caspase‐1/GSDMD pyroptotic signaling	Lipotoxicity, mtDNA leakage, mtROS accumulation	cGAMP ↑, p‐TBK1 ↑, p‐IRF3 ↑; NLRP3 activation; IL‐1β, IL‐18 and TNF‐α ↑, pyroptosis ↑	Cardiac hypertrophy, myocardial injury, and cardiac dysfunction in DCM	[73]
DCM	**In vivo**: C57BL/6J mice with STZ‐induced T1D; db/db mice as T2D model; cardiac AAV9‐mediated Metrnl overexpression or shRNA knockdown, *n* = 6/group **In vitro**: Primary neonatal rat cardiomyocytes exposed to HG, with Metrnl supplementation or knockdown	cGAS–STING activation; LKB1/AMPK/ULK1‐dependent autophagy	Hyperglycemia, diabetes‐induced glucotoxic stress	cGAS ↑, p‐STING ↑, autophagy ↓, ROS ↑, fibrosis ↑, apoptosis ↑, hypertrophy ↑	Cardiac injury and dysfunction	[74]
**Alternative mitochondrial nucleic acid sensing and regulatory modules**						
MI/RI	**In vivo**: WT and MAVS KO mice, LAD ligation 30 min and reperfusion; cardiac specific MAVS knockdown mice **In vitro**: HL‐1 cardiomyocytes with H/R; HEK293T cells transfected with RNA from infarcted myocardium	RIG‐I–MAVS–TAK1–TRAF6–JNK	ROS ↑, Endogenous dsRNA	RIG‐I and MAVS K63 ubiquitination ↑, TAK1 and TRAF6 activation ↑; JNK activation and apoptosis ↑	Larger infarction, worse remodeling and cardiac dysfunction	[75]
MI/RI	**Ex vivo**: Langendorff perfused WT and NLRX1 knockout mouse hearts, baseline 20 min, global ischemia 35 min and reperfusion 90 min; Pig hearts with 90 min ischemia and 3 h reperfusion	NLRX1–mTOR–RISK–mPTP axis	Ischemia and reperfusion stress with post‐IR NLRX1 downregulation	RISK signaling ↓, mPTP opening blocked, mitochondrial Ca^2+^ loading ↑, AMPK ↑	Ischemia and reperfusion injury aggravated; infarct size ↑, functional recovery ↓	[76]
**Immunometabolic and oxidative stress modulators**						
AS	**In vivo**: EC‐specific Ucp2 knockout mice, AAV‐Pcsk9 and WTD‐induced atherosclerosis, partial carotid ligation model **In vitro**: HAECs/HUVECs exposed to OSS or USS	KLF2–UCP2–AMPK–FoxO1 axis; NF‐κB and JNK activation	Disturbed flow and ox‐LDL‐related inflammatory stimulation	UCP2 ↓, AMPK ↓, FoxO1 ↑, adhesion molecules and cytokines ↑, monocyte adhesion ↑	Endothelial inflammation and accelerated atherogenesis	[77]
DCM	**In vivo**: Doxorubicin‐treated WT, SIRT3‐overexpression, and SIRT3‐deficient mice **In vitro**: PRNCs and hiPSC‐derived cardiomyocytes treated with Doxorubicin	SIRT3 decline‐associated mitochondrial oxidative stress and acetylation dysregulation	Doxorubicin exposure; SIRT3 downregulation	SOD2 and IDH2, ROS and 4‐HNE ↑, apoptosis and fibrosis ↑,	LV remodeling, EF ↓, systolic/diastolic dysfunction, DCM progression	[78]
AS	**In vivo**: Macrophage‐specific Rictor KO on ApoE^−/−^ mice, WTD for 8 weeks **In vitro**: Rictor KO macrophages with LPS + ATP or LPS + CC	mTORC2–Akt–FoxO1–IL–1β	Rictor deletion; LPS; ATP; cholesterol crystals	IL‐1β signaling activation, necrotic core expansion; apoptosis ↑	Atherosclerotic lesion area ↑, plaque complexity ↑, macrophage cell death ↑	[79]

*Note*: ↑, elevation/upregulation/activation; ↓, reduction/downregulation/inhibition.

Abbreviations: AAV9, adeno‐associated virus 9; acLDL, acetylated low‐density lipoprotein; ALDH2, aldehyde dehydrogenase 2; ANP, atrial natriuretic peptide; AS, atherosclerosis; ASC, apoptosis‐associated speck‐like protein containing a caspase recruitment domain; BRCC3, BRCA1/BRCA2‐containing complex subunit 3; CH, cardiac hypertrophy; CHD, coronary heart disease; CitH4, citrullinated histone H4; CTX, cardiotoxicity; CXCR2, C‐X‐C motif chemokine receptor 2; DCM, diabetic cardiomyopathy; DHX9, DExH‐box helicase 9; EC, endothelial cell; ER, endoplasmic reticulum; H/R, hypoxia/reoxygenation; HF, heart failure; HFD, high‐fat diet; HG, high glucose; ICAM‐1, intercellular adhesion molecule‐1; IFNAR, interferon‐α/β receptor; IQGAP1, IQ motif‐containing GTPase‐activating protein 1; ISGs, interferon‐stimulated genes; JNK, c‐Jun N‐terminal kinase; KLF2, Krüppel‐like factor 2; LAD, left anterior descending coronary artery; LKB1, liver kinase B1; LPS, lipopolysaccharide; MAVS, mitochondrial antiviral‐signaling protein; MI, myocardial infarction; MI/RI, myocardial ischemia/reperfusion injury; MPO, myeloperoxidase; mPTP, mitochondrial permeability transition pore; mTORC2, mechanistic target of rapamycin complex 2; MYH7, myosin heavy chain 7; NAD, nicotinamide adenine dinucleotide; OCM, obesity‐related cardiomyopathy; ODN, oligodeoxynucleotide; OSS, oscillatory shear stress; OXPHOS, oxidative phosphorylation; P2(7, purinergic receptor P2(7; PA, palmitic acid; PCSK9, proprotein convertase subtilisin/kexin Type 9; PRDX5, peroxiredoxin 5; RISK, reperfusion injury salvage kinase; sEVs, small extracellular vesicles; SIRT3, sirtuin 3; STAT1, signal transducer and activator of transcription 1; STZ, streptozotocin; T1D, Type 1 diabetes; T2D, Type 2 diabetes; TAC, transverse aortic constriction; TET2, tet methylcytosine dioxygenase 2; TRAF6, TNF receptor‐associated factor 6; UCP2, uncoupling protein 2; ULK1, unc‐51‐like kinase 1; Unc93b1, unc‐93 homolog B1; USS, unidirectional shear stress; WTD, Western‐type diet; ZBP1, Z‐DNA‐binding protein 1.

## 4 Mitochondrial Quality Control and Danger Signal Release

Mitochondria are not only central organelles for cellular energy metabolism but also important sources of endogenous danger signals in CVDs [80, 81, 82]. Under pathological stimuli such as ischemia–reperfusion, pressure overload, metabolic stress, and toxic injury, mitochondria may undergo membrane potential depolarization, respiratory chain dysfunction, mtDNA damage, excessive ROS production, and structural disruption [83, 84, 85]. To prevent the persistence of damaged mitochondria, cells rely on MQC systems, including mitochondrial biogenesis, dynamics, mitophagy, proteostasis, and antioxidant defense [86, 87, 88, 89]. Among these mechanisms, mitophagy is particularly important because it selectively removes damaged mitochondria before their components become aberrantly exposed to the cytosol or extracellular space. When MQC becomes insufficient or maladaptive, mitochondria‐derived DAMPs, including mtDNA, mtROS, ATP, CL, and oxidized lipids, may accumulate and provide upstream inputs for sterile inflammatory activation. Therefore, MQC and mitochondrial DAMP release represent a critical interface linking metabolic stress to cardiovascular inflammation.

### 4.1 Mitophagy Pathways and Mitochondrial Quality Control

**FIGURE 2:**
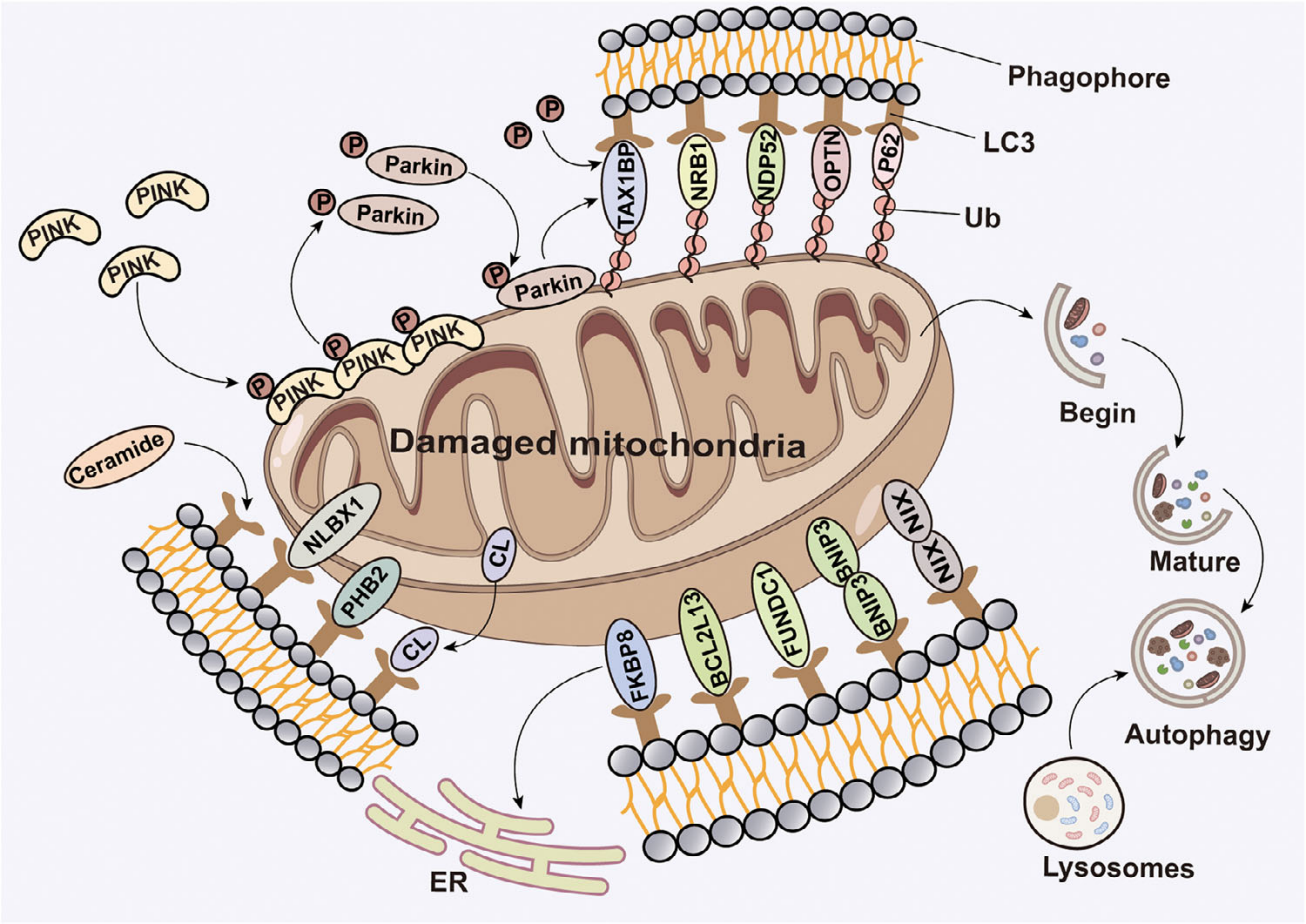
Multifaceted mechanisms of mitophagy activation. Damaged mitochondria are selectively recognized and eliminated through multiple pathways. PINK1/Parkin‐dependent pathway: Upon OMM depolarization, PINK1 accumulates and recruits Parkin, which ubiquitinates mitochondrial proteins. Ubiquitin‐binding adaptors (e.g., p62, NBR1, OPTN, NDP52, TAX1BP1) interact with LC3 to initiate phagophore formation. Receptor‐mediated pathways: OMM proteins such as BNIP3, NIX, FUNDC1, FKBP8, and BCL2L13 harbor LC3‐interacting regions and directly recruit damaged mitochondria to autophagosomes. Lipid and protein signals: Cardiolipin externalization to the OMM facilitates LC3 binding; IMM/mitochondrial matrix proteins such as PHB2 and NLRX1 also participate in recognition. Ceramide signaling further promote mitophagy initiation. Autophagosome–lysosome fusion: Recognized mitochondria are ultimately degraded via autophagosome maturation and lysosomal fusion.

Mitophagy is a central component of MQC that selectively removes damaged or dysfunctional mitochondria through the autophagy–lysosome system. Following mitochondrial injury, damaged mitochondria are recognized, sequestered by autophagic membranes, and delivered to lysosomes for degradation. Through this process, mitophagy maintains mitochondrial homeostasis, preserves bioenergetic stability, and limits the accumulation of mitochondria‐derived danger signals [90, 91, 92]. Mechanistically, mitophagy can be initiated through several distinct but interconnected routes, including the canonical PTEN‐induced putative kinase 1 (PINK1)/cytosolic E3 ubiquitin ligase Parkin (Parkin)‐dependent pathway, receptor‐mediated mitophagy, and lipid‐mediated mitophagy. These pathways converge on microtubule‐associated protein 1 light chain 3 (LC3)‐ or GABA type A receptor‐associated protein (GABARAP)‐associated autophagosome formation and collectively determine whether damaged mitochondria are efficiently cleared or persist as sources of inflammatory DAMPs [93]. The major mitophagy pathways and their functional convergence are summarized in Figure 2.

The PINK1/Parkin pathway is the best‐characterized ubiquitin‐dependent mechanism of mitophagy. Under physiological conditions, PINK1 is imported into healthy mitochondria in a membrane potential‐dependent manner and rapidly degraded after mitochondrial processing. When mitochondria become depolarized or injured, PINK1 accumulates on the outer mitochondrial membrane and recruits Parkin. Activated Parkin ubiquitinates multiple outer mitochondrial membrane proteins, generating ubiquitin signals that are recognized by autophagy adaptors such as sequestosome 1/p62, optineurin (OPTN), nuclear dot protein 52 (NDP52), Tax1‐binding protein 1 (TAX1BP1), and neighbor of BRCA1 gene 1 (NBR1). These adaptors bridge ubiquitinated mitochondria to LC3‐positive autophagic membranes, thereby promoting mitochondrial sequestration and lysosomal degradation [94, 95, 96, 97]. In cardiovascular stress conditions, timely activation of PINK1/Parkin‐mediated mitophagy helps remove damaged mitochondria before excessive ROS generation or mtDNA leakage occurs.

Receptor‐mediated mitophagy provides a ubiquitin‐independent route for mitochondrial clearance. In this pathway, mitochondrial proteins containing LC3‐interacting regions (LIRs) directly recruit LC3 or GABARAP family proteins to damaged mitochondria, thereby coupling mitochondrial recognition to autophagosome formation without requiring Parkin‐mediated ubiquitination [98, 99]. This mechanism is particularly important under hypoxic, ischemic, and metabolic stress conditions, where cells may require rapid and context‐specific mitochondrial clearance. Representative receptors include BCL2/adenovirus E1B 19 kDa protein‐interacting protein 3 (BNIP3), BCL2/adenovirus E1B 19 kDa protein‐interacting protein 3‐like (NIX/BNIP3L), FUN14 domain containing 1 (FUNDC1), and BCL2 like 13 (BCL2L13), each of which links specific stress signals to mitophagy activation.

BNIP3 and NIX are outer mitochondrial membrane proteins that promote mitophagy through their LIR‐dependent interaction with LC3/GABARAP proteins [100, 101, 102, 103]. Under hypoxic or ischemic conditions, BNIP3 expression can be induced by hypoxia‐related signaling, thereby facilitating the clearance of damaged mitochondria and limiting ROS accumulation [104, 105]. NIX functions in a similar manner and contributes to mitochondrial turnover in stress adaptation and cellular remodeling [106, 107, 108]. FUNDC1 is another well‐characterized mitophagy receptor with particular relevance to cardiovascular stress. Its interaction with LC3 is dynamically regulated by phosphorylation and dephosphorylation events, allowing FUNDC1‐mediated mitophagy to respond rapidly to hypoxia and ischemia [109, 110, 111, 112]. BCL2L13, a mammalian functional homolog of yeast Atg32, can also directly bind LC3 and promote mitochondrial clearance, even in contexts where Parkin‐dependent signaling is limited [113, 114, 115].

Beyond these representative receptors and regulatory factors, additional molecules such as FK506‐binding protein 8 (FKBP8), prohibitin 2 (PHB2), and NOD‐like receptor X1 (NLRX1) further expand the regulatory spectrum of receptor‐mediated mitophagy [116, 117, 118, 119, 120]. Although these proteins differ in subcellular localization and modes of activation, collectively they indicate that damaged mitochondria can be recognized and cleared through multiple partially overlapping surveillance systems [119, 121, 122]. From the perspective of inflammatory regulation, receptor‐mediated mitophagy is particularly important because it determines whether mitochondria exposed under hypoxic, oxidative, or metabolic stress can be removed in a timely manner or instead persist and release immunogenic components such as oxidized lipids and mtDNA.

Lipid‐mediated mitophagy further expands the recognition mechanisms of damaged mitochondria. CL, normally enriched in the inner mitochondrial membrane, can translocate to the outer mitochondrial membrane after mitochondrial injury and function as an “eat‐me” signal by interacting with LC3 [123, 124, 125, 126]. In addition, ceramide has been reported to participate in lipid‐regulated mitochondrial recognition and mitophagy under selected stress conditions [127, 128]. Compared with protein receptor‐mediated mechanisms, lipid‐mediated mitophagy highlights the importance of mitochondrial membrane remodeling in quality control. Together, these pathways indicate that mitophagy is not governed by a single molecular route, but by a multilayered surveillance system that senses mitochondrial depolarization, outer membrane protein changes, and lipid redistribution.

From the perspective of inflammatory regulation, the functional significance of these mitophagy pathways lies in their ability to limit the persistence of damaged mitochondria and reduce the release of immunogenic mitochondrial components. When mitophagy is efficient, damaged mitochondria can be cleared before mtDNA, mtROS, CL, or oxidized lipids accumulate to inflammatory levels. By contrast, impaired or maladaptive mitophagy allows dysfunctional mitochondria to persist, thereby increasing the likelihood of mitochondrial DAMP release and downstream inflammatory activation.

### 4.2 Mechanisms of Mitochondrial DAMP Release

When MQC fails to effectively restrain mitochondrial injury, mitochondrial components may escape from their normal compartments and function as DAMPs [20]. Because mitochondria retain several bacterial‐like molecular features, including circular DNA, N‐formyl peptides, CL‐rich membranes, and specific metabolic molecules, their abnormal exposure in the cytosol or extracellular space can be interpreted by the immune system as signals of cellular damage or danger [22, 129]. Among these mitochondria‐derived DAMPs, mtDNA, mtROS, ATP, CL, and oxidized mitochondrial lipids are particularly relevant to sterile cardiovascular inflammation. mtDNA release represents one of the most important links between mitochondrial dysfunction and innate immune activation. Under physiological conditions, mtDNA is confined within the mitochondrial matrix and protected by mitochondrial membranes and nucleoid‐associated proteins. This spatial sequestration prevents mtDNA from being recognized by cytosolic or endosomal immune sensors [20, 130]. However, under pathological conditions such as oxidative stress, mitochondrial depolarization, ischemia–reperfusion injury, or impaired mitophagy, mitochondrial membrane integrity may be disrupted, allowing mtDNA to escape into the cytosol or extracellular environment.

Several mechanisms may contribute to mtDNA release. Severe mitochondrial injury can increase mitochondrial outer membrane permeability, and pore formation mediated by BCL2‐associated X protein (BAX) and BCL2 antagonist/killer 1 (BAK) may allow mitochondrial components to leak into the cytosol. Voltage‐dependent anion channel (VDAC) oligomerization on the outer mitochondrial membrane can also facilitate the release of mtDNA fragments, whereas mitochondrial permeability transition pore (mPTP) opening and mitochondrial swelling may further disrupt mitochondrial membrane architecture and promote mitochondrial content leakage. In addition, ROS‐induced oxidation and fragmentation of mtDNA can increase its immunogenicity and make it more susceptible to release and recognition [131, 132, 133]. These mechanisms indicate that mtDNA release is not a single event, but a consequence of coordinated disturbances in mitochondrial membrane stability, oxidative damage, and quality‐control failure.

mtROS constitute another major category of mitochondrial danger signals. Under physiological conditions, limited ROS production participates in redox signaling and cellular adaptation. During mitochondrial dysfunction, however, impaired electron transport chain activity, membrane potential collapse, and defective antioxidant defense can lead to excessive mtROS accumulation [134]. Excess mtROS not only damages proteins, lipids, and nucleic acids, but also promotes mitochondrial membrane destabilization, mtDNA oxidation, and further DAMP release [132]. In this sense, mtROS act both as direct danger signals and as upstream amplifiers that increase the probability of additional mitochondrial DAMP exposure.

ATP released from injured or dying cells also contributes to mitochondrial danger signaling. Although ATP normally serves as an intracellular energy currency, extracellular ATP can function as a danger signal when released during membrane damage, necrosis, pyroptosis, or severe metabolic stress. In cardiovascular injury, extracellular ATP accumulation may reflect cellular disruption and energetic collapse, and can further participate in inflammatory amplification through purinergic signaling [135, 136]. Thus, ATP release provides another route through which metabolic failure is translated into immune activation.

Mitochondrial membrane lipids also contribute to DAMP output. CL, normally located mainly in the inner mitochondrial membrane, may become externalized or released during mitochondrial injury [137]. Once exposed outside its normal location, CL can serve as a mitochondrial damage signal and participate in inflammatory recognition. Oxidized mitochondrial lipids may further aggravate this process by reflecting both membrane injury and oxidative stress. Therefore, changes in mitochondrial lipid localization and oxidation state are not merely markers of organelle damage, but also part of the mitochondrial DAMP repertoire.

Overall, mitochondrial DAMP release results from the convergence of membrane permeabilization, oxidative injury, defective mitophagy, metabolic collapse, and disrupted mitochondrial lipid organization. These processes determine whether mitochondrial stress remains contained within the organelle or becomes converted into immunologically active danger signal output. Therefore, understanding the mechanisms of mitochondrial DAMP release is essential for defining how MQC failure creates the upstream conditions for sterile inflammatory activation in CVDs.

### 4.3 Threshold Control of Mitochondrial Danger Signal Output

Although mitochondrial injury can generate multiple DAMPs, inflammatory activation does not occur simply because mitochondria are stressed. Under physiological or mildly stressed conditions, MQC systems, antioxidant defenses, and intracellular degradation pathways can limit the accumulation and exposure of damaged mitochondrial components. In this state, mitophagy removes dysfunctional mitochondria, mitochondrial dynamics segregate damaged mitochondrial fragments, and antioxidant systems restrict excessive ROS accumulation [138]. These mechanisms collectively maintain mitochondrial danger signals below the level required for robust immune recognition.

This process can be conceptualized as a mitochondrial DAMP threshold. When mitochondrial injury remains within the buffering capacity of MQC, mitochondria‐derived signals are either repaired, degraded, or spatially contained before they trigger sustained inflammatory sensing. However, when mitochondrial damage becomes excessive, prolonged, or poorly cleared, mtDNA, mtROS, ATP, CL, and oxidized mitochondrial lipids may accumulate beyond the capacity of local control systems [139, 140]. Once this threshold is exceeded, mitochondrial stress is converted from a primarily metabolic disturbance into an immunologically active danger signal output.

Mitophagy is a central determinant of this threshold. Efficient mitophagy raises the threshold for inflammatory activation by reducing the pool of damaged mitochondria that can release mtDNA, generate excessive mtROS, or expose abnormal membrane lipids [141]. Conversely, impaired mitophagy lowers this threshold by allowing dysfunctional mitochondria to persist and continuously produce danger signals. Importantly, excessive or dysregulated mitophagy may also become maladaptive when mitochondrial clearance exceeds metabolic renewal, leading to mitochondrial depletion, impaired ATP production, and further cellular stress [142]. Therefore, threshold control depends not only on the presence or absence of mitophagy, but also on whether mitochondrial clearance remains appropriately matched to injury burden and metabolic demand.

The DAMP threshold is also shaped by disease stage and cellular context. In acute ischemia–reperfusion injury, large amounts of mitochondrial danger signals may be released within a short period, rapidly overwhelming the buffering capacity of MQC and antioxidant systems [142]. In chronic vascular or metabolic diseases, by contrast, mitochondrial DAMP output may remain lower in intensity but persist for a prolonged period, gradually maintaining inflammatory activation. Cardiomyocytes, endothelial cells, macrophages, and fibroblasts may also differ in their mitochondrial reserve, antioxidant capacity, and sensitivity to DAMP exposure, further contributing to cell type‐specific inflammatory outcomes [143].

To further support this threshold‐based interpretation, Table 2 summarizes representative evidence linking mitophagy alterations to mitochondrial danger signal accumulation and cardiovascular injury across different disease settings. These studies indicate that mitophagy abnormalities may manifest as insufficient mitophagy, impaired mitophagic flux, compensatory activation, or excessive mitochondrial clearance. Despite these differences, their shared pathological consequence is often the persistence of damaged mitochondria and the accumulation of mitochondrial danger signals, including mtROS, mtDNA leakage, loss of mitochondrial membrane potential (MMP), CL exposure, oxidized lipids, and structural mitochondrial disruption. Therefore, the pathological significance of mitophagy alteration is not determined simply by whether mitophagy is increased or decreased, but by whether mitochondrial clearance remains appropriately matched to injury burden and metabolic demand.

**TABLE 2:** Mitophagy alterations and associated mitochondrial danger signals underlying cardiovascular injury.

Disease	Experimental model	Mitophagy alteration	Mitochondrial danger signals	Cardiovascular consequence	Mechanistic implication	Refs.
MI/RI	**In vivo**: SD rats, LAD ligation 30 min and reperfusion 4 h **In vitro**: H9c2 cells, Hypoxia/reoxygenation 12 h/4 h aerobic exercise, choline, or methoctramine intervention	I/R‐induced excessive PINK1/Parkin‐mediated mitophagy	Mitochondrial swelling and cristae disruption indicated mitochondrial injury	Cardiac dysfunction, fibrosis/collagen deposition ↑, CK and LDH release ↑, cardiomyocyte apoptosis ↑	Exercise‐activated M2AChR and inhibited PINK1/Parkin and PERK/eIF2α/ATF4 signaling, thereby reducing ER stress, apoptosis, and myocardial MI/RI injury	[144]
MI/RI	**In vitro**: H/R neonatal rat cardiomyocytes **Ex vivo**: Langendorff perfused rat hearts, *n* = 5/group	I/R‐induced excessive PINK1/Parkin‐mediated mitophagy	Mitochondrial dysfunction, fragmentation, cytochrome c release	Myocardial injury ↑, apoptosis ↑, impaired cardiac function	Notch1 suppresses PTEN‐PINK1–MFN2–Parkin signaling to restrain excessive mitophagy	[145]
MI/RI	**In vivo**: Male SD rats, LAD ligation 30 min and reperfusion 120 min, *n* = 6/group **In vitro**: H9c2 cells subjected to hypoxia 4 h and reoxygenation 2 h	BNIP3‐mediated mitophagy ↑	Mitochondrial dysfunction and ROS‐related danger signaling	Myocardial injury ↑, infarct size ↑, cardiomyocyte apoptosis ↑	Inhibition of HIF‐1α‐BNIP3 increases cardiomyocyte apoptosis, indicating a cardioprotective role of HIF‐1α‐BNIP3‐mediated mitophagy during MI/RI	[146]
MI/RI	**In vivo**: C57BL/6J mice, intratracheal PM exposure for 24 h, followed by LAD ligation for 30 min and reperfusion for 3 h **In vitro**: PM treated H9c2 cardiomyocytes with hypoxia 6 h/reoxygenation 12 h	Excessive BNIP3‐mediated mitophagy	ROS ↑, MMP ↓, ATP ↓, mitochondrial fission ↑	Cardiac dysfunction, infarction, apoptosis ↑	PM aggravates myocardial I/R injury by promoting ROS‐driven mitochondrial fission and BNIP3‐mediated maladaptive mitophagy, leading to apoptosis	[147]
MI/RI	**In vivo**: SD rats, STZ‐induced diabetic rats with LAD occlusion 30 min and reperfusion 2 h, sham *n* = 6/group, I/R *n* = 12/group **In vitro**: Hypoxia/reoxygenation and high glucose treatment of neonatal rat cardiomyocytes from newborn SD rats	Impaired, decreased, and rhythm‐disordered mitophagy	Mitochondrial ROS ↑, MMP ↓	Myocardial injury aggravated, infarct size ↑, cardiac dysfunction ↑	HDAC3 upregulation disrupts the Rev‐erbα/BMAL1 pathway, suppresses mitophagy, and aggravates diabetic MI/R injury	[148]
MI/RI	**Ex vivo**: SD rats, Langendorff perfusion, 30 min ischemia/120 min reperfusion, *n* = 10/group	PINK1/Parkin‐mediated mitophagy ↓	Mitochondrial respiratory dysfunction, oxidative stress	Cardiac dysfunction, infarction, CK‐MB/cTnI release ↑	Mitophagy activation preserves mitochondrial function and contributes to cardioprotection	[149]
MI/RI	**In vivo**: C57BL/6J mice and SD rats, LAD ligation 30 min and reperfusion 24 h, *n* = 5/group **In vitro**: Hypoxia/reoxygenation treatment of neonatal rat cardiomyocytes	Excessive BNIP3L and PINK1/Parkin‐mediated mitophagy	Mitochondrial structural damage, cristae loss, ATP ↓, LDH ↑	Infarct size ↑, impaired cardiac function ↑, apoptosis↑	CSE nanocarriers protected against I/R by inhibiting CHOP/GRP78/eIF2α‐associated ER stress and excessive mitophagy	[150]
MI	**In vivo**: Male C57BL/6J mice, 8 weeks old, *n* = 6/group; MI induced by permanent LAD ligation; resistance exercise intervention	PINK1/Parkin‐mediated mitophagy ↓	Oxidative stress	Cardiac function ↑, myocardial fibrosis ↓	Exercise‐activated Irisin/FNDC5‐PINK1/Parkin signaling, enhanced mitophagy, and improved post‐MI cardiac remodeling	[151]
MI	**In vivo**: C57BL/6 mice, 8–10 weeks old, *n* = 7/group, LAD ligation for 7 days; NMCMs with 9 h anoxia	Mitophagy flux impaired	Damaged mitochondria accumulation, ROS ↑, MMP ↓	Cardiac dysfunction ↑, apoptosis ↑, fibrosis ↑, mitochondrial injury ↑	NDP52 recruits TBK1/RAB7 to promote RAB7 phosphorylation and autophagosome–lysosome fusion, thereby restoring mitophagy flux	[152]
MI	**In vivo**: Male C57BL/6 mice, 6–8 weeks; LAD ligation MI model; BaP 10 mg/kg/week for 4 weeks, i.p.; *n* = 6/group **In vitro**: OGD‐induced H9c2 cardiomyocytes with BaP	PINK1/Parkin‐mediated mitophagy ↓	mPTP opening ↑; ROS ↑	Infarct size ↑; cardiac injury markers CK‐MB, cTnI, LDH ↑; myocardial injury aggravated	BaP activates AhR signaling, suppresses PINK1/Parkin‐mediated mitophagy, promotes mPTP opening and ROS accumulation, leading to NLRP3 inflammasome activation and pyroptosis, thereby aggravating MI injury	[153]
MI	**In vitro**: Neonatal C57BL/6 mouse cardiomyocytes; 24 h hypoxia	Mitophagy flux impaired	Damaged mitochondria accumulation, ROS ↑, ATP ↓, OCR ↓, complex I/III activity ↓, mitochondrial fission ↑, fusion/biogenesis ↓	Cardiomyocyte injury ↑; inflammation ↑; apoptosis ↑; contractile dysfunction ↑	Opa1 promotes mitophagy and mitochondrial biogenesis, inhibits excessive fission, reduces oxidative stress, and preserves mitochondrial function under hypoxia	[154]
HF	**In vivo**: ICR male mice, LAD ligation/CAL for 2 weeks, *n* = 8/group **In vitro**: H9c2 cells, OGD for 12 h	PINK1/Parkin‐mediated mitophagy ↓	ROS ↑, MMP ↓; mitochondrial fragmentation ↑	Cardiac dysfunction, hypertrophy, infarct and fibrosis ↑, myocardial injury ↑	Omentin1 alleviated MI‐induced HF by activating SIRT3/FOXO3a, restoring PINK1/Parkin‐mediated mitophagy, and improving mitochondrial homeostasis	[155]
HF	**In vivo**: C57BL/6J mice, TAC‐induced pressure‐overload HF; *n* = 6/group **In vitro**: ISO‐treated neonatal mouse cardiomyocytes	PINK1/Parkin‐mediated mitophagy ↓	ROS ↑, ATP ↓	Left ventricular remodeling and fibrosis ↑; systolic dysfunction ↑	MRT protects the failing heart by activating HIF‐1α/Parkin‐mediated mitophagy	[156]
HF	**In vivo**: C57BL/6 mice, LAD ligation to induce MI/HF, hypoxia acclimation at 11% O_2_, 8 h/day for 28 days, *n* = 5/group **In vitro**: H9c2 cells with OGD	FUNDC1‐mediated mitophagy ↓	Mitochondrial respiratory dysfunction and structural damage; ROS ↑	Cardiac dysfunction and remodeling after MI, including EF ↓, FS ↓, BNP ↑, fibrosis ↑	HA alleviates post‐AMI HF by enhancing FUNDC1‐mediated mitophagy and limiting apoptosis	[157]
AS	**In vivo**: C57BL/6J mice, HFD‐fed ApoE^−/−^ mice, *n* = 11/group, Ldlr^−/−^ mice, *n* = 6/group **In vitro**: ox‐LDL‐treated J774A.1 macrophages; LPS and ATP‐stimulated WT/ApoE^−/−^/Ldlr^−/−^ macrophages	Impaired mitophagy	ROS ↑, mtDNA↑; MMP ↓	Vascular inflammation ↑, macrophage pyroptosis ↑, foam cell formation ↑, plaque progression ↑	NLRP3 activation worsens mitochondrial injury and suppresses Parkin‐mediated mitophagy, promoting atherosclerosis	[158]
AS	**In vitro**: HUVECs were treated with ox‐LDL	Impaired mitophagy	ROS ↑, MMP ↓, mPTP opening ↑, ATP ↓, Ca^2+^ overload ↑,	Endothelial apoptosis ↑	PTEN upregulation inhibits AMPK–CREB–Mfn2‐mediated mitophagy, thereby exacerbating mitochondrial dysfunction and endothelial injury	[159]
HCM	**In vivo**: Male SD rats, X‐ray‐induced cardiac hypertrophy, *n* = 6/group **In vitro**: X‐ray treatment of H9c2 cells	Excessive BNIP3 and PINK1/Parkin‐mediated mitophagy	ROS ↑, mitochondrial damage ↑, mitochondrial mass ↓, mito‐lysosome colocalization ↑	Cardiac hypertrophy with dysfunction	SUMO2‐mediated SH3GLB1 SUMOylation activates PINK1/Parkin mitophagy, promoting ROS accumulation and cardiac hypertrophy	[160]
SCM	**In vivo**: WT and DUSP1TG mice, LPS *i.p*., 20 mg/kg **In vitro**: HL‐1 cardiomyocytes treated with LPS 10 µg/mL for 24 h	FUNDC1‐mediated mitophagy ↓	ROS ↑, MMP ↓; mitochondrial respiration ↓, ATP ↓	Cardiac systolic/diastolic dysfunction, apoptosis ↑, inflammatory injury ↑	DUSP1 restores FUNDC1‐mediated mitophagy to protect against septic cardiomyopathy	[161]
DLCM	**In vivo**: Global Bmal1 knockout mice, *n* = 4/group **In vitro**: BMAL1 knockout hESC‐cardiomyocytes	BNIP3‐mediated mitophagy ↓	Hyperfused mitochondria ↑, cristae disruption ↑, mitochondrial dysfunction and OXPHOS ↓, ATP ↓	Cardiomyocyte apoptosis ↑, Ventricular dilation and wall thinning, EF ↓, FS ↓, impaired contractility	BMAL1 deficiency impairs BNIP3‐dependent mitophagy and mitochondrial quality control, thereby promoting DCM	[162]
DCM	**In vivo**: C57BL/6 db/db mice **In vitro**: Neonatal Wistar rat cardiomyocytes	PINK1/Parkin‐mediated mitophagy ↓	ROS ↑, MMP ↓, mitochondrial fragmentation ↑, cytosolic Parkin ubiquitination ↑, USP8 sulfhydration ↓	Cardiac dysfunction, mitochondrial injury, apoptosis ↑	H_2_S restores USP8‐mediated Parkin deubiquitination and mitochondrial translocation, thereby rescuing mitophagy and attenuating DCM	[163]
SICD	**In vivo**: SD rats were injected with 10 mg/kg LPS, *n* = 6/group **In vitro**: Neonatal rat cardiomyocytes, LPS 10 µg/mL for 24 h	PINK1/Parkin‐mediated mitophagy ↓	ROS ↑, cytochrome c release ↑, MMP and ATP ↓	Myocardial injury and cardiac dysfunction ↑	TMBIM1 recruits Parkin to mitochondria, promotes mitophagy, and reduces cardiac injury	[164]
CR	**In vivo**: WT and FUNDC1^−/−^ mice; LFD or HFD for 10 weeks	FUNDC1‐mediated mitophagy ↓	ROS ↑, lipid peroxidation ↑, oxidative DNA damage ↑, mitochondrial injury ↑	Hypertrophy ↑, fibrosis ↑, cardiac dysfunction ↑	FUNDC1 deficiency worsens HFD‐induced cardiac injury via mitophagy defect‐associated ferroptosis	[165]
CTX	**In vivo**: Male C57BL/6 mice gavaged with sorafenib for 8 weeks, *n* = 6/group **In vitro**: Primary neonatal mouse cardiomyocytes and HL‐1 cells treated with sorafenib	Excessive mitophagy	Mitochondrial Ca^2+^ ↑, ATP ↓, MAM contact ↑	Necroptosis ↑, cardiac dysfunction ↑, hypertrophy ↑	Sorafenib inhibited mTORC1 and TFEB phosphorylation, promoted TFEB nuclear translocation, and enhanced mitophagy‐mediated MFN2 degradation	[166]
CTX	**In vitro**: AC16 human cardiomyocytes treated with OPD’	Excessive PINK1/Parkin‐mediated mitophagy	ROS ↑, MMP ↓, PGC‐1α ↓, Nrf2/HO‐1 ↓	Cardiomyocyte apoptosis ↑	OPD’ induces PINK1/Parkin‐dependent excessive mitophagy, leading to mitochondrial damage and apoptosis	[167]
CTX	**In vivo**: HFD‐induced NASH in SD rats, *n* = 10/group	Impaired mitophagy	UCP2 ↓, SOD ↓, HIF‐1 ↑, VEGF ↓, NF‐κB, IL‐6, and TNF‐α ↑	Myocardial injury ↑, inflammation ↑, CK‐MB, LDH, and cTnI ↑	NASH suppresses PI3K/AKT, impairs mitophagy, and promotes oxidative stress, inflammation, and cardiotoxicity; BM‐MSCs‐EV/BM‐MSCs reverse these changes	[168]

*Note*: ↑, elevation/upregulation/activation; ↓, reduction/downregulation/inhibition.

Abbreviations: ACSL4, acyl‐CoA synthetase long‐chain family member 4; AhR, aryl hydrocarbon receptor; AMI, acute myocardial infarction; ATF4, activating transcription factor 4; BaP, benzo[a]pyrene; BMAL1, brain and muscle ARNT‐like 1; BNP, B‐type natriuretic peptide; CAL, coronary artery ligation; CaMKIIδ, Ca^2^^+^/calmodulin‐dependent protein kinase II delta; CHOP, C/EBP homologous protein; CK, creatine kinase; CK‐MB, creatine kinase‐MB; CR, cardiac remodeling; CREB, cAMP response element‐binding protein; cTnI, cardiac troponin I; DLCM, dilated cardiomyopathy; EF, ejection fraction; eIF2α, eukaryotic initiation factor 2 alpha; FS, fractional shortening; GRP78, glucose‐regulated protein 78; HCM, hypertrophic cardiomyopathy; HDAC3, histone deacetylase 3; HIF‐1α, hypoxia‐inducible factor 1 alpha; I/R, ischemia/reperfusion; ISO, isoproterenol; LDH, lactate dehydrogenase; LFD, low‐fat diet; M2AChR, M2 acetylcholine receptor; MAM, mitochondria‐associated membrane; MMP, mitochondrial membrane potential; MRT, resistance training; mTORC1, mechanistic target of rapamycin complex 1; Notch1, Notch homolog 1; OCR, oxygen consumption rate; OGD, oxygen‐glucose deprivation; OPD’, Ophiopogonin D’; PERK, protein kinase RNA‐like endoplasmic reticulum kinase; PM, particulate matter; PTEN, phosphatase and tensin homolog; RAB7, Ras‐related protein Rab‐7a; Rev‐erbα, nuclear receptor subfamily 1 group D member 1; SCM, septic cardiomyopathy; SH3GLB1, SH3‐domain GRB2‐like endophilin B1; SICD, sepsis‐induced cardiac dysfunction; SUMO2, small ubiquitin‐like modifier 2;TFEB, transcription factor EB; TMBIM1, transmembrane BAX inhibitor motif‐containing 1; USP8, ubiquitin‐specific peptidase 8.

Overall, threshold control of mitochondrial danger signal output provides a key mechanistic link between MQC failure and inflammatory activation in CVDs. When mitochondrial damage is effectively contained, DAMP generation may remain compatible with adaptive stress responses and tissue repair. Once mitochondrial danger signals exceed the buffering capacity of quality‐control and resolution mechanisms, however, they provide sustained upstream input for inflammatory sensing and amplification. This threshold‐dependent transition helps explain why mitochondrial dysfunction can produce either adaptive compensation or pathological inflammation depending on disease stage, cellular context, and the balance between mitochondrial clearance and metabolic demand.

## 5 From Mitochondrial DAMPs to Inflammatory Network Activation

The preceding section clarified how MQC failure permits the accumulation and release of mitochondrial DAMPs. Once these signals exceed the buffering capacity of MQC and local resolution mechanisms, they are no longer confined to mitochondrial stress responses but become available for immune recognition. In this context, mitochondria‐derived signals can activate multiple inflammatory sensing platforms, including cytosolic DNA sensing, endosomal DNA recognition, inflammasome activation, and redox‐sensitive transcriptional programs [169]. Therefore, the central issue is not merely whether mitochondrial damage occurs, but how mitochondria‐derived DAMPs are translated into broader inflammatory network activation. Among these danger signals, mtDNA is particularly important because it links mitochondrial injury to nucleic acid‐sensing pathways. However, mitochondrial DAMPs do not act through a single linear route. Instead, mtDNA, mtROS, oxidized lipids, CL, and other mitochondrial stress signals can jointly engage cGAS–STING, TLR9, NLRP3, NF‐κB‐related inflammatory programs, and additional mitochondrial immune interfaces. At the same time, mitophagy acts as an upstream brake on this process by reducing the pool of damaged mitochondria that can release these signals. When this brake fails, inflammatory activation can feed back to further impair mitochondrial homeostasis, establishing a self‐amplifying circuit between MQC dysfunction and sterile inflammation [16].

Accordingly, this section focuses on how mitochondrial DAMPs are recognized by inflammatory sensing systems and how this recognition is integrated into the broader inflammatory network of CVDs. We first discuss mtDNA sensing through cGAS–STING and TLR9, then examine how mitophagy restrains inflammasome and STING signaling, and finally consider the bidirectional feedback between inflammation and mitophagy as well as its implications for adaptive immune remodeling.

### 5.1 mtDNA Sensing by cGAS–STING and TLR9

mtDNA is one of the most immunologically active mitochondrial DAMPs released during mitochondrial injury. Under physiological conditions, mtDNA is spatially confined within mitochondria, thereby avoiding inappropriate recognition by innate immune sensors. When mitochondrial membrane integrity is disrupted, however, mtDNA may enter the cytosol, extracellular space, or endosomal compartments, where it can activate distinct nucleic acid‐sensing pathways. Among these, cGAS–STING and TLR9 represent two major routes through which mtDNA stress is converted into sterile inflammatory signaling.

The cGAS–STING pathway is a central cytosolic DNA‐sensing mechanism. When mtDNA leaks into the cytosol, cGAS can recognize dsDNA and catalyze the generation of cGAMP, which subsequently activates STING and its downstream TBK1–IRF3 and NF‐κB‐related signaling outputs [41, 170, 171]. In CVDs, mitochondrial dysfunction, oxidative stress, ischemia–reperfusion injury, metabolic disturbance, and cardiomyocyte death can all increase the probability of mtDNA leakage and thereby promote cGAS–STING activation [10, 20]. Through IFN‐I responses and proinflammatory transcriptional programs, this pathway links intracellular mitochondrial damage to broader innate immune activation.

TLR9 provides another important route for mtDNA recognition. Unlike cGAS–STING, which senses cytosolic DNA, TLR9 is an endosomal DNA sensor that recognizes unmethylated cytosine–phosphate–guanine (CpG) motifs. These motifs are abundant in bacterial DNA and are also present in mtDNA because of its bacterial evolutionary origin [129]. Following cellular injury, extracellular or phagocytosed mtDNA can be internalized into endosomal compartments, where it activates TLR9 signaling. In cardiovascular contexts, TLR9 activation has been linked to inflammatory responses after myocardial injury and ischemia–reperfusion damage, contributing to leukocyte recruitment and cytokine production within injured cardiac tissue [172, 173].

These two mtDNA‐sensing routes are complementary rather than mutually exclusive. Cytosolic mtDNA preferentially engages cGAS–STING, whereas extracellular or phagocytosed mtDNA can be routed toward endosomal TLR9. Both pathways can converge on inflammatory transcriptional programs and contribute to sterile inflammatory amplification, but their relative contribution may vary depending on disease stage, cell type, subcellular localization of mtDNA, and the efficiency of mitochondrial clearance. Thus, mtDNA sensing should be understood as a compartment‐dependent process in which the location of mtDNA exposure strongly influences the downstream inflammatory outcome.

In addition to mtDNA sensing, mitochondrial RNA (mtRNA)‐related stress may also participate in innate immune activation. The cytosolic RNA sensor retinoic acid‐inducible gene I (RIG‐I) and its adaptor protein mitochondrial antiviral signaling protein (MAVS) form a signaling axis that detects abnormal RNA species. Under conditions of mitochondrial dysfunction, mtRNA may become aberrantly exposed to the cytosol and potentially engage RIG‐I‐dependent signaling [130]. Conversely, not all mitochondrial immune interfaces promote inflammation. Mitochondrial‐localized regulators such as NLRX1 can modulate MAVS‐associated signaling and restrain excessive immune activation, thereby helping maintain immune homeostasis under stress conditions [116].

Taken together, mitochondrial nucleic acid sensing is not limited to the mitophagy–mtDNA–cGAS–STING axis. Instead, mtDNA and mtRNA can engage a multilayered recognition network composed of cytosolic DNA sensors, endosomal DNA sensors, RNA‐sensing platforms, and mitochondrial‐localized immune regulators. This broader view helps explain why mitochondrial DAMP release can produce different inflammatory outputs across CVD settings, depending on the route of nucleic acid exposure, cellular context, and surrounding metabolic state.

### 5.2 Mitophagy as an Upstream Brake on Inflammasome and STING Signaling

Mitophagy not only preserves mitochondrial quality homeostasis but also acts as an upstream brake on inflammatory activation. Mitochondria are major sources of multiple DAMPs, and when damaged mitochondria are not cleared in a timely manner, signals such as mtDNA, ROS, and oxidized lipids may become aberrantly exposed and detected by multiple PRRs [22]. By selectively eliminating damaged mitochondria, mitophagy reduces the generation and release of mitochondrial danger signals and thereby limits the probability that inflammatory sensing systems will be activated. Conversely, when mitophagy is impaired, dysfunctional mitochondria persist and release mitochondria‐derived signals, thereby activating multiple inflammatory sensing and amplification mechanisms [141].

This upstream regulatory function extends beyond a simple reduction in DAMP production. Mitophagy also influences the spatial accessibility of PRR ligands and the persistence of mitochondrial signaling platforms. When damaged mitochondria remain within cells, mtDNA or mtRNA is more likely to enter the cytosol or endosome‐like compartments through membrane rupture, mitochondria‐derived vesicle release, or phagocytic processing, thereby providing sustained stimulation for nucleic acid‐sensing PRRs. By eliminating these potential sources of danger signals, mitophagy constrains PRR‐accessible substrates before they surpass the threshold for immune recognition. In this sense, mitophagy not only limits danger signal generation but also reshapes the intracellular distribution and duration of inflammatory ligands [16, 92, 174].

The restraining effect of mitophagy is particularly important for NLRP3 inflammasome activation. NLRP3 is highly responsive to the overall cellular stress state, and mitochondrial dysfunction, mtROS accumulation, mitochondrial membrane instability, and cytosolic mtDNA exposure are among the major upstream inputs that support its activation [38, 175, 176]. When mitophagy is efficient, damaged mitochondria are removed before persistent mtROS production and mtDNA leakage occur, thereby reducing the opportunity for mitochondrial DAMPs to participate in inflammasome assembly. In contrast, impaired mitophagy allows dysfunctional mitochondria to accumulate, creating a local stress environment that favors NLRP3 oligomerization, caspase‐1 activation, IL‐1β/IL‐18 maturation, and pyroptotic amplification [174, 177, 178].

Mitophagy may also influence the spatial organization of inflammasome signaling. Under cellular stress conditions, inflammasome components may assemble in subcellular regions enriched with damaged mitochondria, suggesting that dysfunctional mitochondria can provide a localized platform for inflammatory signaling [179, 180]. Efficient mitophagic clearance reduces the persistence of these abnormal mitochondrial microdomains and thereby limits the maintenance of inflammasome‐active intracellular environments [181]. Once NLRP3 activation is established, however, caspase‐1 activation, cytokine release, ion flux disturbance, and pyroptosis can further aggravate mitochondrial damage and increase the release of intracellular danger signals, creating a reciprocal amplification loop between mitochondrial injury and inflammasome activation [38, 182, 183].

Mitophagy also acts as an upstream brake on cGAS–STING signaling by limiting the cytosolic availability of mtDNA. Under preserved mitochondrial homeostasis, damaged mitochondria are cleared before their membrane integrity declines to the point of mtDNA leakage. When mitophagy is impaired, mtDNA can accumulate in the cytosol and activate cGAS, leading to cGAMP production, STING activation, and downstream TBK1–IRF3‐ and NF‐κB‐related inflammatory outputs [141, 184]. In this way, mitophagy determines not only the amount of mtDNA available for DNA sensing, but also the duration and spatial distribution of mtDNA exposure.

The relationship between mitophagy and STING signaling is also bidirectional. Activated STING can recruit autophagy‐related machinery through its LIR and participate in autophagosome formation, thereby promoting the degradation of STING itself and limiting persistent DNA‐sensing activity [185, 186]. At the same time, sustained STING activation can alter oxidative stress and metabolic states, increasing the demand for mitochondrial turnover and indirectly influencing MQC systems [187, 188]. Therefore, mitophagy and cGAS–STING signaling form a coupled regulatory module centered on mitochondrial homeostasis rather than a simple one‐way inhibitory relationship.

Taken together, mitophagy restrains inflammatory activation by reducing mitochondrial DAMP generation, limiting the spatial accessibility of nucleic acid ligands, preventing NLRP3‐supportive mitochondrial stress environments, and restricting sustained cGAS–STING activation. When this upstream brake fails, mtROS accumulation, mtDNA leakage, inflammasome activation, and STING‐dependent inflammatory signaling may become mutually reinforcing. Thus, mitophagy functions as a threshold‐regulating mechanism that determines whether mitochondrial stress remains locally contained or progresses into broader inflammatory network activation.

### 5.3 Bidirectional Crosstalk Between Inflammation and Mitophagy

The relationship between mitophagy and inflammatory signaling is not unidirectional. Although mitophagy can restrain inflammatory activation by limiting mitochondrial DAMP release, persistent inflammation can in turn impair mitochondrial homeostasis, disrupt MQC, and further aggravate mitochondrial dysfunction. In CVDs, mitochondrial injury and inflammatory activation therefore rarely occur as isolated events. Instead, they form a mutually reinforcing regulatory circuit in which damaged mitochondria provide inflammatory inputs, while inflammatory signaling further weakens mitochondrial stability and quality‐control capacity.

Under chronic inflammatory conditions, continuous production of mediators such as TNF‐α, IL‐1β, and other proinflammatory cytokines activates NF‐κB‐, MAPK‐, and JNK‐related signaling pathways, thereby reshaping cellular metabolism and promoting oxidative stress [189, 190]. These inflammatory signals can impair mitochondrial electron transport efficiency, induce membrane depolarization, and sustain ROS production, leading to direct oxidative damage to mitochondrial proteins, lipids, and nucleic acids. Excessive ROS further promotes mtDNA oxidation, weakens mitochondrial membrane integrity, and increases the probability of mtDNA leakage. In this setting, inflammation not only responds to mitochondrial damage but also becomes an active driver of further mitochondrial deterioration [191].

Inflammatory signaling also interferes with several key regulatory nodes of MQC. The peroxisome proliferator‐activated receptor gamma coactivator 1‐α (PGC‐1α)/sirtuin 3 (SIRT3)/mitochondrial transcription factor A (TFAM) axis plays an important role in mitochondrial biogenesis, antioxidant defense, and mtDNA maintenance [192]. Persistent inflammation can suppress PGC‐1α activity and impair SIRT3‐mediated deacetylation, thereby weakening mitochondrial antioxidant capacity and reducing the stability of mtDNA [193, 194]. Once this biogenesis‐antioxidant maintenance axis is compromised, damaged mitochondria are less efficiently replaced, and mitochondrial stress becomes more likely to persist. This creates a permissive environment for sustained mtROS generation and mitochondrial DAMP accumulation.

Mitochondrial dynamics are also affected by inflammatory stress. Persistent activation of inflammatory pathways can promote dynamin‐related protein 1 (DRP1)‐mediated mitochondrial fission while impairing optic atrophy 1 (OPA1)‐ and mitofusin‐dependent fusion maintenance, resulting in a fragmented mitochondrial network with reduced structural and functional stability [195]. Fragmented mitochondria are more prone to membrane potential loss, respiratory dysfunction, and excessive ROS production. Under physiological conditions, mitochondrial fission can help segregate damaged mitochondrial fragments for subsequent mitophagic clearance. However, when inflammatory stress is prolonged, excessive fission combined with impaired mitophagic flux may instead lead to the accumulation of dysfunctional mitochondrial fragments, thereby increasing the burden of DAMP release.

In addition to disrupting mitochondrial biogenesis and dynamics, inflammation can directly impair mitophagy and autophagic flux. Sustained oxidative stress and inflammatory signaling may interfere with PINK1/Parkin‐dependent mitochondrial recognition, autophagosome formation, and autophagosome–lysosome fusion, ultimately reducing the efficiency of damaged mitochondrial clearance [196]. Under these conditions, dysfunctional mitochondria persist within cells and continue to produce mtROS, release oxidized mtDNA, and expose abnormal mitochondrial lipids. Thus, inflammation can convert mitophagy from a protective quality‐control response into an insufficient or maladaptive process, further promoting mitochondrial danger signal accumulation.

As mitochondrial damage accumulates, mitochondria‐derived DAMPs can further reactivate inflammatory sensing pathways. mtROS, oxidized mtDNA, CL, and oxidized mitochondrial lipids can provide sustained inputs for PRR‐related inflammatory priming, NLRP3 inflammasome activation, and cGAS–STING signaling [21, 197, 198]. This creates a feed‐forward loop in which mitochondrial injury promotes inflammatory activation, and inflammatory activation further damages mitochondria. In this process, inflammation gradually shifts from a transient response to tissue injury into a self‐sustaining driver of mitochondrial dysfunction and sterile inflammatory maintenance.

**FIGURE 3:**
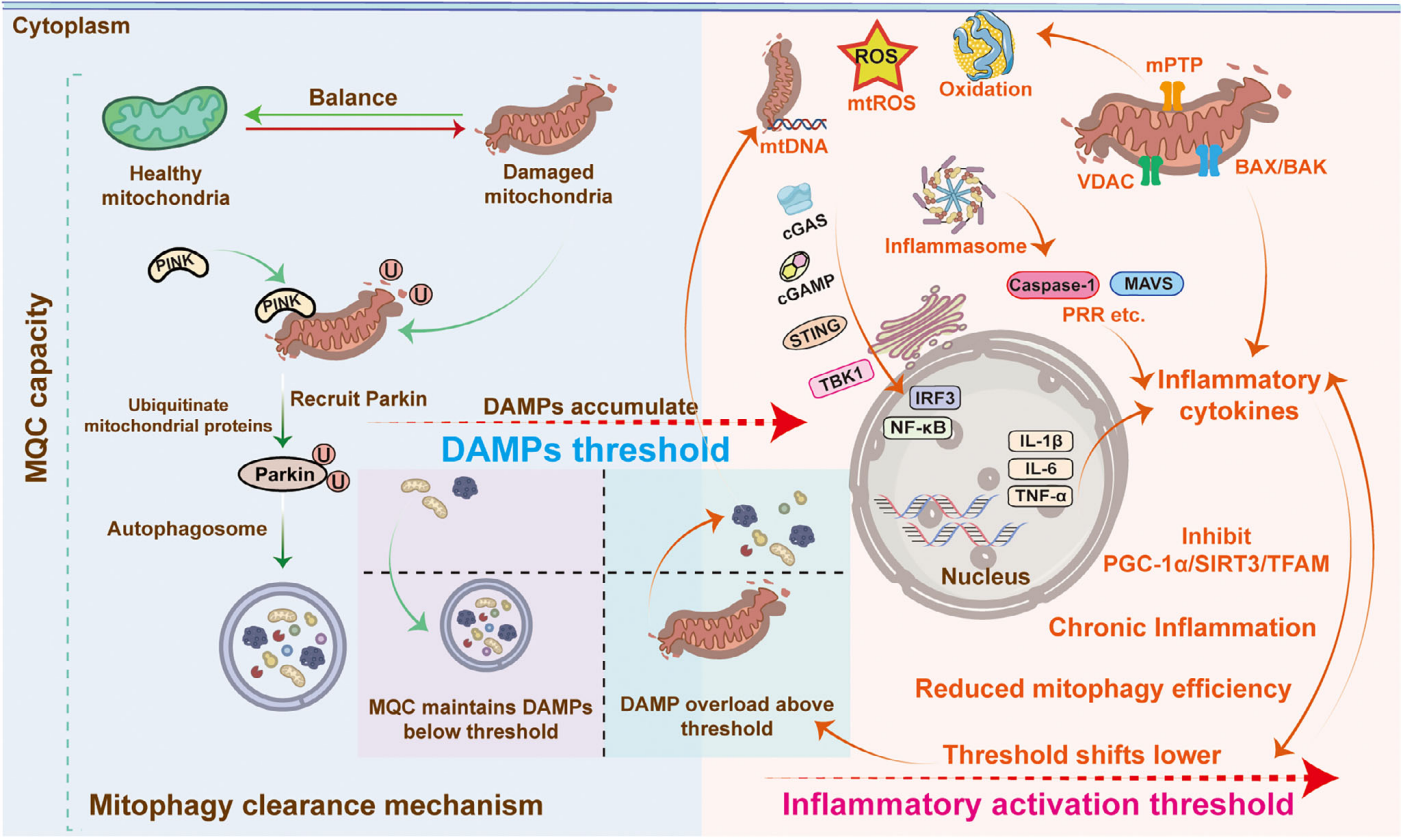
Dual‐threshold model of mitophagy failure and inflammatory amplification in cardiovascular diseases. Under homeostatic conditions, MQC and mitophagy restrict the accumulation of mitochondria‐derived DAMPs and maintain inflammatory activation below threshold. When MQC capacity declines, damaged mitochondria accumulate and release mtDNA, mtROS, and oxidized mitochondrial components, thereby activating PRR‐related signaling, inflammasomes, and the cGAS–STING pathway. The resulting inflammatory cytokine production further disrupts mitochondrial homeostasis, suppresses PGC‐1α/SIRT3/TFAM‐associated MQC, reduces mitophagy efficiency, and lowers the inflammatory activation threshold. This bidirectional feedback establishes a self‐sustaining inflammatory amplification circuit driven by dual‐threshold disequilibrium.

From a systems perspective, this reciprocal process can be understood through two interrelated thresholds. The first is the mitochondrial DAMP threshold, which reflects whether mitochondria‐derived danger signals have exceeded the buffering capacity of MQC. The second is the inflammatory activation threshold, which reflects the minimum level of danger signal input required for inflammatory sensing and amplification pathways to generate a sustained response. Under physiological or mildly stressed conditions, MQC, antioxidant defenses, and degradation pathways maintain mitochondrial danger signals below the level required for robust immune activation. However, when mitochondrial injury persists or quality‐control capacity declines, the basal burden of intracellular danger signals rises, while persistent inflammatory signaling further lowers the threshold for immune activation [174, 199]. As illustrated in Figure 3, impairment of MQC promotes mitochondrial DAMP accumulation, whereas sustained inflammation increases cellular sensitivity to these danger signals, thereby establishing a self‐reinforcing amplification circuit.

This dual‐threshold disequilibrium helps explain why inflammation may persist even when the initial injurious stimulus is no longer dominant. Once mitochondrial danger signal burden remains chronically elevated and inflammatory sensing systems become more easily triggered, relatively low‐level mitochondrial stress may be sufficient to maintain inflammatory output. Meanwhile, inflammatory transcriptional programs driven by NF‐κB, MAPK, and interferon‐related pathways can further reprogram cellular metabolism and redox status, aggravating mitochondrial dysfunction and weakening mitophagic efficiency [200]. In this way, the system progressively loses its capacity to return to homeostasis, and the inflammatory response becomes locked into a chronic maintenance state.

This bidirectional feedback is highly relevant to CVDs. In acute ischemia–reperfusion injury, abrupt mitochondrial damage, ROS burst, calcium overload, and inflammatory cell recruitment may rapidly overwhelm MQC and push the system beyond its buffering capacity. In chronic diseases such as atherosclerosis, HF, and metabolic cardiomyopathy, lower intensity but persistent mitochondrial injury and inflammatory signaling may gradually sustain the feedback loop over time [68, 201, 202]. Although these disease settings differ in temporal pattern and dominant cell type, they share a common feature: persistent disequilibrium between mitochondrial danger signal burden, quality‐control capacity, and inflammatory activation threshold.

Therefore, the pathological consequence of mitophagy‐inflammation crosstalk is not determined by a single molecule or pathway alone. Rather, it depends on whether mitochondrial injury can be contained before inflammatory sensing becomes self‐sustaining. Once the feedback loop between MQC failure and inflammatory activation is established, it can promote endothelial dysfunction, immune‐cell infiltration, cardiomyocyte injury, fibroblast activation, fibrosis, and adverse cardiovascular remodeling [141, 196]. This bidirectional crosstalk provides a mechanistic basis for understanding how mitochondrial dysfunction is converted from a metabolic abnormality into a persistent inflammatory driver in CVDs.

### 5.4 Implications for Adaptive Immune Remodeling

Beyond innate immune inflammation, mitochondrial DAMP‐driven inflammatory networks may also influence adaptive immune remodeling in CVDs. Mitochondrial injury and impaired mitophagy can reshape antigen presentation, interferon‐related signaling, immune‐cell metabolism, and the inflammatory microenvironment, thereby extending mitochondrial stress signals from innate immune sensing to adaptive immune regulation [203, 204, 205]. This connection is particularly important in chronic cardiovascular conditions, where persistent danger signal exposure and incomplete resolution may sustain immune‐cell activation and promote long‐term tissue remodeling.

First, MQC dysfunction may influence adaptive immunity by altering antigen processing and presentation. Mitochondrial injury, excessive ROS production, and oxidized mitochondrial components can modify intracellular proteolytic environments and affect major histocompatibility complex (MHC)‐dependent antigen presentation [203]. In parallel, mitochondria‐derived molecular patterns can be internalized by antigen‐presenting cells, promoting dendritic cell maturation, costimulatory molecule expression, and T cell priming [203, 204, 206]. When mitophagy is efficient, damaged mitochondria and their immunogenic components are cleared in a timely manner, thereby limiting the persistence of antigenic stimulation. In contrast, impaired mitophagy may increase the availability of mitochondrial antigens and danger signals, creating conditions that favor sustained adaptive immune activation.

Second, mtDNA‐driven nucleic acid sensing provides a critical bridge between innate and adaptive immunity. Cytosolic mtDNA can activate cGAS–STING signaling and induce IFN‐I responses, which not only participate in innate antiviral‐like programs but also regulate T cell activation, cluster of differentiation 8 positive (CD8^+^) T cell effector function, and immune memory formation [207]. In CVDs, persistent mitochondrial injury or defective mitophagy may therefore maintain an IFN‐I‐enriched inflammatory environment, allowing mitochondrial stress to influence adaptive immune differentiation and persistence. This mechanism may be especially relevant in chronic inflammatory settings, where low level but sustained nucleic acid sensing can continuously shape local immune tone.

Third, mitochondrial homeostasis directly regulates immune‐cell metabolism. Different T cell subsets rely on distinct metabolic programs: effector T cells generally favor glycolysis, whereas memory T cells and regulatory T cells depend more strongly on mitochondrial oxidative phosphorylation and fatty acid oxidation. By preserving mitochondrial integrity and metabolic flexibility, mitophagy helps maintain appropriate immune‐cell differentiation and function. When MQC is disrupted, excessive ROS production, impaired oxidative phosphorylation, and metabolic imbalance may alter T cell fate, promote proinflammatory effector responses, and weaken immune tolerance [208, 209, 210]. Thus, mitochondrial dysfunction can influence adaptive immunity not only through DAMP release, but also by reshaping the metabolic programs that determine immune‐cell behavior.

From a broader immune network perspective, the impact of the mitophagy‐inflammation crosstalk extends beyond T cell regulation alone. Mitochondrial DAMPs, inflammatory cytokines, and redox imbalance can also affect macrophage polarization, dendritic cell function, B cell responses, and the persistence of chronic inflammatory niches. Through these mechanisms, MQC dysfunction may contribute to sustained immune‐cell infiltration, impaired resolution, and progressive tissue remodeling [203, 204]. In the cardiovascular microenvironment, such adaptive immune remodeling can further interact with fibroblast activation, extracellular matrix deposition, and vascular inflammation, thereby linking mitochondrial stress to long‐term structural injury.

Mitochondrial stress‐induced inflammatory signaling may also connect with complement and coagulation pathways. Inflammatory mediators, oxidized lipids, and damage‐associated signals can activate complement cascades and coagulation systems, while complement fragments and coagulation factors in turn amplify immune‐cell recruitment and inflammatory responses [211]. This complement–coagulation–immune interaction may promote immunothrombosis and aggravate microvascular injury, particularly in ischemic and vascular disease settings. Therefore, mitochondrial danger signaling should be viewed not only as a trigger of innate immune pathways, but also as a broader organizer of immune remodeling across innate immunity, adaptive immunity, immunometabolism, and immunothrombotic responses.

Taken together, the transition from mitochondrial DAMP release to inflammatory network activation has consequences that extend beyond immediate innate immune signaling. By influencing antigen presentation, IFN‐I responses, immune‐cell metabolism, adaptive immune persistence, and immunothrombotic coupling, impaired mitophagy and mitochondrial danger signaling may contribute to chronic immune remodeling in CVDs. This provides a mechanistic basis for understanding how mitochondrial dysfunction can sustain inflammation over long time scales and promote adverse cardiovascular remodeling.

## 6 Stage‐, Cell‐, and Threshold‐Dependent Inflammation in Cardiovascular Diseases

Although the preceding sections have systematically outlined the major inflammatory signaling pathways in CVDs from a modular perspective, these pathways do not operate in isolation within actual pathological settings. Instead, they are embedded within a higher order regulatory perspective shaped by disease stage, cellular context, and signal intensity. This multidimensional regulation determines not only whether inflammatory responses are initiated, but also how they are amplified, sustained, resolved, or converted into pathological remodeling.

Accumulating evidence indicates that inflammatory activation in CVDs is strongly stage dependent rather than linearly progressive [7, 212]. In acute injury, such as myocardial ischemia–reperfusion, inflammation is typically rapid, intense, and closely linked to abrupt danger signal release. In chronic conditions, such as atherosclerosis and HF, inflammatory activation more often persists at a lower intensity but over a longer duration. These temporal differences are further shaped by cell type‐specific inflammatory networks, in which cardiomyocytes, endothelial cells, macrophages, fibroblasts, and other immune cells assume distinct but interconnected roles in inflammatory sensing, amplification, metabolic regulation, and tissue remodeling [213, 214, 215].

Beyond temporal and cellular dimensions, the threshold of inflammatory activation is also critical for determining disease outcome. Inflammatory signaling does not function as a simple binary process, but instead operates across a continuum of intensity, duration, and regulatory control. Differences in signal magnitude and persistence can shift inflammation from adaptive repair toward maladaptive remodeling [216]. Mitochondrial stress, redox imbalance, MQC failure, and immunometabolic dysfunction act as key modulators of this threshold, thereby influencing whether inflammatory responses remain controllable or become self‐sustaining.

**FIGURE 4:**
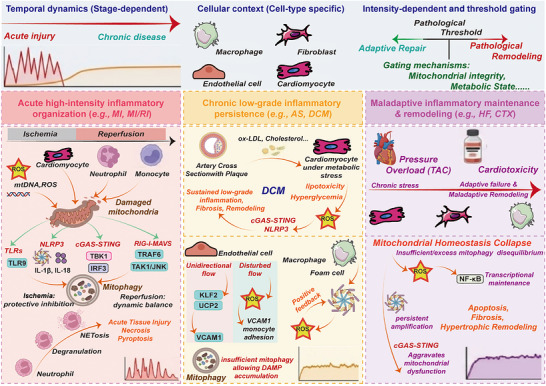
Stage‐, cell‐, and threshold‐dependent framework of inflammatory heterogeneity in cardiovascular diseases. Inflammatory responses in CVDs are jointly shaped by temporal dynamics, cell type‐specific organization, and intensity‐dependent threshold gating. Representative disease settings can be mapped into three major patterns: Acute high‐intensity inflammation in myocardial infarction/myocardial ischemia–reperfusion injury, chronic low‐grade inflammatory persistence in atherosclerosis and diabetic cardiomyopathy, and maladaptive inflammatory maintenance and remodeling in pressure overload, cardiotoxicity, and related cardiomyopathic conditions. Mitochondrial integrity, mitophagy, redox balance, and metabolic state function as key gating mechanisms that determine whether inflammation supports adaptive repair or progresses to pathological remodeling.

Overall, cardiovascular inflammation should be understood as a multidimensional system governed by stage‐dependent dynamics, cell type‐specific organization, and threshold‐based regulation. This stage‐cell‐threshold perspective and its application to representative CVD settings are summarized in Figure 4. The following sections use representative CVDs settings to illustrate how distinct inflammatory patterns arise from different combinations of temporal course, dominant cellular contributors, danger signal burden, and threshold‐gating status.

### 6.1 Temporal Dynamics of Inflammatory Activation

Inflammatory activation in CVDs does not follow a simple linear pattern of continuous intensification. Instead, it undergoes dynamic reprogramming across different stages of disease development, with marked differences in initiating signals, dominant pathways, activation intensity, and functional consequences. This temporal organization largely determines whether inflammation contributes to acute injury control, reparative remodeling, chronic maintenance, or progressive tissue dysfunction.

In acute injury settings, such as myocardial ischemia–reperfusion, inflammatory responses are rapidly initiated and can reach high intensity within a short period [213]. During the early phase, ischemia and reperfusion induce ionic imbalance, ROS burst, mitochondrial depolarization, membrane permeability changes, and the release of mitochondria‐derived danger signals. These events rapidly activate innate immune sensing systems and create a highly amplified inflammatory environment [68, 217]. Subsequent inflammasome activation, inflammatory cell death, cytokine release, and neutrophil recruitment further expand tissue injury. Experimental evidence suggests that early control of inflammasome activation or mitochondrial danger signal release during ischemia–reperfusion can reduce infarct size and improve cardiac function, indicating that the peak intensity of acute inflammation is a critical determinant of tissue damage [218].

In chronic vascular diseases, such as atherosclerosis, inflammatory activation follows a different temporal pattern. Rather than presenting as a transient burst, inflammation is maintained by persistent low‐level stimulation from oxidized lipids, cholesterol crystals, cellular debris, endothelial stress, and macrophage activation [214]. TLR‐related priming, inflammasome activation, and nucleic acid‐sensing pathways may all participate, but their pathological significance lies less in acute peak intensity than in prolonged persistence and failure of resolution [40, 219]. Sustained low‐grade inflammation gradually promotes endothelial dysfunction, foam cell death, plaque progression, cell death, and plaque destabilization, ultimately increasing the risk of acute cardiovascular events [219].

In HF and other remodeling‐associated conditions, inflammation further evolves into a state of chronic maintenance driven by metabolic imbalance and persistent tissue stress. Pressure overload, impaired energy metabolism, mitochondrial dysfunction, and neurohumoral activation continuously expose cardiomyocytes and stromal cells to low‐level danger signals [220]. At the same time, NAD^+^ depletion, reduced sirtuin activity, oxidative stress, and impaired resolution mechanisms weaken the ability of tissues to terminate inflammation effectively [194, 221]. Under these conditions, inflammation may no longer appear as an obvious acute peak, but its prolonged persistence can drive fibrosis, cardiomyocyte dysfunction, and progressive ventricular remodeling.

Importantly, these temporal patterns are not strictly separated. Acute inflammation normally transitions toward resolution and repair after tissue injury. However, when mitochondrial damage persists, MQC becomes insufficient, or metabolic regulation remains disrupted, this transition may fail, allowing an acute inflammatory response to evolve into chronic low‐grade activation [222]. Therefore, the temporal dynamics of inflammation determine not only changes in signal intensity, but also whether inflammatory responses are terminated, resolved, or maintained as drivers of long‐term cardiovascular remodeling.

Overall, inflammatory responses in CVDs should be viewed as stage‐dependent processes that shift between acute amplification, chronic maintenance, and maladaptive remodeling. This temporal perspective provides a necessary basis for understanding why the same inflammatory pathway may have different consequences depending on disease stage and intervention timing.

### 6.2 Cell Type‐Specific Inflammatory Architecture in Cardiovascular Tissues

Inflammatory responses in CVDs are not governed by a single dominant cell type, but emerge from coordinated interactions among multiple cellular populations within cardiovascular tissues [7]. Different cell types do not simply activate identical inflammatory programs. Instead, they occupy distinct functional positions within the inflammatory network, including danger sensing, immune‐cell recruitment, inflammatory amplification, metabolic integration, and structural remodeling [223].

Endothelial cells often serve as an entry and coordination interface for cardiovascular inflammation. In early atherosclerosis, disturbed flow, oxidized lipids, oxidative stress, and inflammatory cytokines induce endothelial activation, leading to increased expression of adhesion molecules and chemokines that promote monocyte adhesion and transendothelial migration [224, 225]. This process does not necessarily require overt inflammatory amplification at the beginning, but sustained endothelial activation can determine the spatial distribution and intensity of downstream immune‐cell recruitment. In this sense, endothelial cells function as gatekeeping nodes that regulate the entry of inflammatory responses into the vascular wall and microvascular environment.

Once monocytes enter cardiovascular tissue and differentiate into macrophages, the inflammatory network shifts toward signal amplification. In atherosclerotic plaques, macrophages internalize oxidized lipids and cholesterol crystals, become foam cells, and serve as major sites of inflammasome activation and cytokine production [219, 226]. In myocardial ischemia–reperfusion injury, recruited macrophages also participate in the propagation of inflammatory signaling and the transition from injury clearance to reparative or fibrotic remodeling [227, 228]. Depending on disease stage and local microenvironment, macrophages may either amplify tissue injury through proinflammatory cytokine release and pyroptosis or contribute to resolution by clearing debris and supporting repair.

Cardiomyocytes represent another key cellular component of the cardiovascular inflammatory network. Unlike immune cells, cardiomyocytes mainly function as metabolic‐inflammatory coupling nodes. Under ischemic stress, pressure overload, toxic injury, or metabolic disturbance, mitochondrial dysfunction in cardiomyocytes can increase ROS generation, promote mtDNA leakage, and activate cell‐intrinsic inflammatory signaling pathways [68, 202]. These inflammatory signals may arise from intracellular metabolic disequilibrium even before extensive immune‐cell infiltration occurs. During HF progression, cardiomyocytes can continuously release low‐level danger signals and paracrine mediators, thereby sustaining local inflammation and influencing immune cells and fibroblasts [229, 230].

Fibroblasts become particularly important during the remodeling phase. In postinfarction repair, pressure overload, and chronic cardiomyopathy, inflammatory mediators activate fibroblasts and promote their differentiation into myofibroblasts. These cells produce extracellular matrix components and directly contribute to fibrosis and structural remodeling [231]. Fibroblasts are not merely passive effector cells; they can also sense inflammatory signals, produce cytokines and chemokines, and interact with immune cells and cardiomyocytes. Therefore, persistent fibroblast activation can convert inflammatory signaling into long‐term architectural changes in cardiovascular tissues.

Importantly, the functional meaning of the same inflammatory pathway may differ across cell types. Nucleic acid‐sensing pathways in macrophages are often linked to inflammatory amplification, whereas in cardiomyocytes they may primarily reflect mitochondrial or nuclear DNA stress [172, 202]. Similarly, oxidative stress in endothelial cells mainly affects barrier function, adhesion molecule expression, and leukocyte recruitment, whereas in cardiomyocytes it more directly influences mitochondrial energetics, contractile function, and cell survival [232, 233]. This cell type‐specific heterogeneity helps explain why the same inflammatory module can produce different pathological outcomes across cardiovascular settings.

Overall, cardiovascular inflammation should be understood as a cell‐organized network rather than a uniform tissue‐wide response. Endothelial cells regulate inflammatory entry, macrophages amplify and shape immune responses, cardiomyocytes couple metabolic stress to inflammatory signaling, and fibroblasts translate persistent inflammation into structural remodeling. This cellular division of labor provides an important basis for understanding the heterogeneity of inflammatory phenotypes in CVDs and for developing stage‐ and cell‐informed therapeutic strategies.

### 6.3 Threshold Gating and Dual Inflammatory Outcomes

Although inflammation in CVDs is commonly regarded as a major driver of tissue injury and pathological remodeling, it is not uniformly detrimental. The biological consequence of an inflammatory response is determined not simply by whether a given pathway is activated, but by the intensity, duration, cellular context, and regulatory state of this activation [215, 234]. Within a controlled range, inflammatory responses contribute to debris clearance, removal of damaged cells, recruitment of reparative immune cells, and initiation of tissue repair. Moderate inflammatory activation may also limit the persistent accumulation of damage‐associated components and facilitate the removal of damaged mitochondria and cellular debris, thereby preventing further spread of danger signals within the local microenvironment. Once inflammatory signaling exceeds its regulatory range or persists beyond the required repair window, however, the same response may become maladaptive and promote oxidative stress, cell death, fibrosis, and functional decline [21, 235, 236, 237, 238, 239]. Sustained inflammatory activation may also disrupt MQC and enhance danger signal release, further reinforcing pathological amplification [240, 241].

This transition from adaptive inflammation to pathological inflammation is controlled by several threshold‐gating mechanisms. First, mitochondrial integrity determines the magnitude of danger signal input. Under homeostatic conditions, mitochondria confine potentially immunogenic molecules such as mtDNA, CL, and oxidized mitochondrial components within appropriate compartments. When mitochondrial injury becomes excessive or poorly controlled, these molecules may become aberrantly exposed to the cytosol or extracellular space, thereby increasing the effective input into inflammatory sensing pathways [129, 130, 140, 242]. In this sense, mitochondria act not only as sources of inflammatory signals but also as input‐gating nodes that determine whether cellular stress is converted into immune activation.

Second, cellular metabolic and redox states regulate the amplification threshold of inflammatory signaling. NAD^+^ availability, sirtuin activity, AMPK/mTOR balance, mitochondrial oxidative phosphorylation, and antioxidant defense collectively influence whether cells can buffer inflammatory stress or enter a state of persistent activation [52, 243, 244, 245]. In particular, AMPK generally supports autophagy, mitochondrial homeostasis, and metabolic adaptation, whereas excessive mTOR activity may favor anabolic stress and proinflammatory maintenance, thereby influencing the amplification threshold of inflammatory signaling. When NAD^+^ is depleted, sirtuin activity is impaired, or ROS accumulates excessively, the capacity to restrain NF‐κB activation, inflammasome assembly, and nucleic acid‐sensing persistence may decline. These changes do not simply increase inflammatory output; they lower the threshold at which inflammatory pathways become sustained.

Third, resolution mechanisms determine whether inflammation can be terminated after its initial function has been fulfilled. Under physiological repair conditions, inflammatory responses are gradually resolved through debris clearance, macrophage phenotype transition, regulatory immune activity, restoration of mitochondrial homeostasis, and proresolving mediators [246]. However, when DAMP generation persists, MQC remains insufficient, or metabolic imbalance weakens repair capacity, inflammatory termination may be delayed or blocked. In this situation, inflammation is no longer a transient response to injury but becomes a self‐maintaining process that contributes to chronic remodeling and tissue dysfunction [140]. Thus, failure of inflammatory termination should not be viewed simply as passive prolongation of inflammation, but as a key control layer that determines whether an acute or reparative response progresses into a chronic pathological state.

Therefore, inflammatory outcomes in CVDs should be understood as threshold‐dependent rather than simply pathway dependent. In this framework, mitochondrial status governs danger signal input, metabolic and redox regulation shapes signal amplification, and resolution mechanisms determine signal duration. Moderate and time‐limited activation can support clearance and repair, whereas excessive, prolonged, or poorly resolved activation can drive pathological amplification. This threshold‐gating concept helps explain why the same inflammatory modules, including TLR signaling, NLRP3 inflammasome activation, cGAS–STING signaling, and mitophagy‐related responses, may exert different or even apparently opposing effects across disease stages and cellular contexts. It also provides the conceptual basis for interpreting the distinct inflammatory patterns discussed in the following disease settings.

### 6.4 Acute High‐Intensity Inflammation in Ischemic Injury

Ischemic injury represents one of the most characteristic acute high‐intensity inflammatory patterns in CVDs, with myocardial infarction and myocardial ischemia–reperfusion injury as representative conditions. Unlike chronic inflammatory settings, in which danger signals accumulate gradually, ischemic injury is characterized by abrupt onset, rapid expansion, and tight coupling among multiple inflammatory modules. Persistent ischemia induces cellular necrosis, mitochondrial damage, ionic imbalance, and metabolic collapse, leading to the rapid release of large amounts of DAMPs within a short time frame. These signals can simultaneously activate PRRs, inflammasomes, nucleic acid‐sensing pathways, and stress kinase cascades [140, 247]. During reperfusion, ROS bursts, calcium overload, mitochondrial permeability changes, and rapid immune‐cell recruitment further drive the inflammatory network beyond critical thresholds and into an amplified pathological state [213, 248]. Therefore, ischemic injury does not represent activation of a single inflammatory pathway, but rather an acute high‐intensity inflammatory configuration generated by the rapid convergence of danger sensing, inflammatory priming, and signal amplification.

From a temporal perspective, the inflammatory response in ischemic injury is strongly stage dependent. During the early ischemic phase, damaged cardiomyocytes and stromal cells release endogenous danger signals, including oxidized lipids, heat shock proteins, mtROS, mtDNA, and other nucleic acid and protein fragments. These signals provide upstream inputs for TLR‐associated inflammatory initiation and establish an NF‐κB‐dependent priming state that increases the responsiveness of downstream amplification modules [21, 37, 129]. At the same time, mitochondrial depolarization, elevated ROS levels, altered membrane permeability, and disrupted ionic homeostasis create favorable conditions for NLRP3 inflammasome assembly and activation, driving the inflammatory network from danger signal sensing to effector amplification [38, 177]. Accordingly, during the acute ischemic phase, TLR–NF‐κB signaling mainly mediates early danger recognition and inflammatory priming, whereas NLRP3 converts these inputs into IL‐1β/IL‐18 release, pyroptotic cell death, and propagation of tissue injury.

At the level of MQC, mitophagy first determines the input burden imposed on this high‐intensity inflammatory network. Moderate enhancement of mitophagy can reduce the upstream source of mitochondrial danger signals before inflammatory amplification becomes established. For example, benzo[a] pyrene (BaP) can inhibit PINK1 accumulation on mitochondrial membranes by activating aryl hydrocarbon receptor (AhR), thereby impairing Parkin and LC3 recruitment. This disruption facilitates abnormal mPTP opening and excessive ROS generation, ultimately amplifying NLRP3 inflammasome activation, pyroptosis, and myocardial injury [153]. Conversely, OPA1 overexpression can enhance mitophagy by promoting mitochondrial fusion and upregulating autophagy related 5 (ATG5), Parkin, and Beclin‐1, whereas exercise training can activate the Irisin/FNDC5‐PINK1/Parkin axis, promote LC3‐II formation, reduce p62 accumulation, enhance OPA1, and suppress DRP1‐mediated excessive fission, thereby improving MQC [151, 154]. These findings indicate that, in the early phase of acute ischemia, the protective role of mitophagy does not lie simply in enhancing autophagic activity, but in reducing danger signal input before the inflammatory network is driven into a high‐activation state.

However, once reperfusion begins, the pathological emphasis shifts from danger signal clearance to prevention of uncontrolled amplification. Reperfusion‐associated ROS burst, calcium overload, and mitochondrial permeability transition can rapidly increase mitochondrial damage and inflammatory output. Under these conditions, excessive or prolonged mitophagy may become maladaptive because it may remove not only irreversibly damaged mitochondria but also mitochondrial populations that retain compensatory capacity, thereby weakening energy supply and aggravating cardiomyocyte vulnerability. Exercise training can attenuate ROS accumulation and apoptosis during reperfusion by activating M2AChR signaling and suppressing excessive PINK1/Parkin‐mediated mitophagy [144]. Notch1 can reduce reperfusion injury by suppressing PTEN and PINK1 and restoring PI3K/AKT activity, while nano‐CSE particles can inhibit aberrant autophagosome formation by downregulating PINK1/Parkin and NIX [145, 150]. In contrast, particulate exposure can induce excessive expression of BNIP3 and LC3B and exacerbate reperfusion injury, whereas exosomes enriched in miR‐221/222 reduce ROS and alleviate tissue damage by suppressing BNIP3, LC3B, and PUMA expression [147]. These observations suggest that the apparently conflicting conclusions that either enhancing or inhibiting mitophagy is beneficial in myocardial ischemia/reperfusion injury (MI/RI) are not truly contradictory, but instead reflect differences in the pathological time window under investigation. The former generally corresponds to injury control during ischemia, whereas the latter more often reflects stages after reperfusion when inflammatory amplification and energetic disequilibrium have already become prominent.

This time dependence is further modified by metabolic background and circadian regulation. In diabetic MI/RI models, excessive HDAC3 activity disrupts Rev‐erbα/BMAL1 oscillation and suppresses the C/EBPβ/BNIP3‐related mitophagy program, leading to rhythm‐disordered mitochondrial clearance, increased mtROS, reduced MMP, and aggravated myocardial injury [148]. These findings suggest that appropriate mitophagic flux in ischemia–reperfusion injury depends not only on the absolute level of mitophagy, but also on whether mitochondrial clearance remains dynamically matched to metabolic state, injury stage, and temporal rhythm [249]. Under metabolic stress conditions, the threshold for inflammatory activation may therefore be lowered, making the myocardium more vulnerable to acute ischemic damage.

Nucleic acid‐sensing pathways further contribute to this acute high‐intensity inflammatory organization. During ischemic injury, mitochondrial damage and cardiomyocyte death can promote mtDNA leakage, thereby activating DNA‐sensing pathways such as TLR9 and cGAS–STING. When mtDNA‐driven cGAS–STING activation is sustained, downstream TBK1–IRF3 and NF‐κB outputs can reinforce cytokine production, immune‐cell recruitment, and tissue remodeling.

In diabetic MI/RI, downregulation of mitofusin 2 (MFN2) impairs mitochondrial fusion and promotes mtDNA leakage, which activates the cGAS–STING–TBK1–IRF3 cascade and upregulates inflammatory mediators such as TNF‐α, IL‐1β, and NLRP3. Restoration of MFN2 or inhibition of STING can both attenuate inflammation and cardiac dysfunction [68]. These findings indicate that cGAS–STING does not function as an isolated output pathway in acute ischemic injury, but functions as part of an integrated inflammatory network that shares upstream mitochondrial danger signal input with TLR and inflammasome pathways.

In addition to DNA sensing, RNA sensing pathways may also be incorporated into the inflammatory network of acute ischemic injury. During MI/RI, damaged cardiomyocytes generate endogenous double‐stranded RNA, which activates the RIG‐I–MAVS pathway. MAVS then aggregates and recruits TRAF6 and TAK1, further engaging MAPK/JNK signaling and promoting cardiomyocyte apoptosis [75]. Both systemic MAVS deficiency and cardiomyocyte‐specific MAVS knockdown have been reported to reduce infarct size, leukocyte infiltration, apoptosis, and cardiac dysfunction in ischemia–reperfusion settings. These findings indicate that nucleic acid sensing in acute ischemic injury is not limited to mtDNA‐centered pathways. Instead, mtDNA‐driven TLR9/cGAS–STING signaling and dsRNA‐driven RIG‐I–MAVS signaling act as complementary routes through which intracellular nucleic acid stress is converted into inflammatory and cell death responses.

At the cellular level, ischemic injury also exhibits a clear functional sequence. Injured cardiomyocytes are not only the initial source of danger signals but may also release mitochondria‐derived components through extracellular vesicles. For example, Ambra1‐positive cardiomyocyte‐derived sEVs enriched in mtDNA can promote fibroblast activation and inflammatory remodeling after ischemic injury [69]. Neutrophils are among the earliest effector cells recruited during reperfusion, and their degranulation, MPO release, and NET‐associated responses mark the transition from intracellular stress to tissue‐level inflammatory propagation [250, 251, 252]. Monocytes and macrophages subsequently participate in debris clearance, cytokine production, and the transition from inflammatory amplification to repair. Fibroblasts become more prominent during later remodeling, where inflammatory signaling is converted into extracellular matrix deposition and structural remodeling [247]. Evidence related to TLR9 further suggests that DNA‐sensing programs can extend beyond acute immune activation and participate in fibroblast‐mediated postinfarction remodeling [57]. Thus, cardiomyocytes, neutrophils, macrophages, and fibroblasts do not participate in parallel in a static manner, but sequentially assume distinct roles in danger signal release, inflammatory amplification, clearance, and structural remodeling.

From the threshold perspective, ischemic injury most clearly exemplifies high‐intensity input and rapid threshold crossing. Moderate inflammatory activation is necessary for clearance of necrotic cells, removal of damaged mitochondria, and initiation of repair. However, when mitochondrial DAMPs, nucleic acid stress, ROS bursts, calcium overload, and immune‐cell recruitment rise simultaneously within a short period, the combined burden can exceed the buffering capacity of MQC, antioxidant defense, and local resolution systems. Once this threshold is crossed, TLR–NF‐κB priming, NLRP3 inflammasome amplification, TLR9/cGAS–STING‐mediated DNA sensing, and RIG‐I–MAVS‐mediated RNA sensing may collectively drive pyroptosis, apoptosis, excessive immune infiltration, fibrotic activation, and adverse remodeling [7, 26, 129]. Mitochondrial gating factors such as NLRX1 may further influence this threshold by regulating RISK/mTOR signaling, mPTP dynamics, mitochondrial calcium loading, and myocardial tolerance to acute ischemia–reperfusion stress [76]. Therefore, the outcome of ischemic injury is determined not only by activation of individual inflammatory pathways, but by whether the inflammatory network as a whole is pushed beyond a controllable threshold.

Overall, acute ischemic injury represents a high‐intensity inflammatory organizational mode characterized by abrupt danger signal release, rapid coupling of inflammatory modules, time‐dependent mitophagy responses, nucleic acid‐sensing amplification, and sequential cellular propagation. In this setting, mitophagy shapes the upstream burden of mitochondrial DAMPs, TLR–NF‐κB signaling establishes inflammatory priming, NLRP3 drives inflammatory amplification and pyroptotic execution, and nucleic acid‐sensing pathways extend mitochondrial damage into broader immune activation. This disease setting therefore provides a representative example of how inflammation can shift rapidly from adaptive clearance and repair toward pathological amplification when danger signal input exceeds the threshold capacity of cardiovascular tissues.

### 6.5 Chronic Low‐Grade Inflammation in Vascular and Metabolic Disease

Unlike ischemic injury, which is characterized by acute high‐intensity inflammatory amplification within a short time window, vascular and metabolic CVDs more commonly exhibit low‐intensity but persistent inflammatory maintenance. Atherosclerosis and diabetic cardiomyopathy are representative examples of this pattern. In these settings, the inflammatory network is not driven by the abrupt release of large amounts of DAMPs, but is instead sustained by long‐term inputs derived from oxidized lipids, cholesterol crystals, hyperglycemia, lipotoxicity, endothelial stress and persistent mitochondrial dysfunction. The pathological hallmark is therefore not an exceptionally high inflammatory peak, but the chronic persistence of danger signal burden above the homeostatic range. As a result, danger sensing, inflammatory priming, inflammasome activation, nucleic acid recognition, and immunometabolic dysregulation may coexist at relatively low but sustained levels, ultimately driving vascular remodeling, myocardial fibrosis, and progressive functional decline.

In atherosclerosis, this chronic inflammatory organization first appears as a persistently primed vascular microenvironment. Oxidized low‐density lipoprotein, cholesterol crystals, extracellular matrix fragments, and cellular debris continuously stimulate endothelial cells and immune cells, thereby maintaining TLR‐associated signaling and NF‐κB‐dependent transcriptional programs. Unlike the burst‐like pattern observed in ischemia–reperfusion injury, inflammatory activation in atherosclerosis usually does not have a sharply defined peak. Instead, endothelial activation, monocyte recruitment, macrophage retention, foam cell formation, and cytokine production are maintained over long periods, creating a state of chronic vascular inflammation. NLRP3 inflammasome activation and nucleic acid‐sensing pathways also participate in this process, but their pathological significance lies more in sustained amplification and failure of resolution than in acute inflammatory intensity.

This chronic priming state is shaped not only by lipid‐derived stimuli but also by hemodynamic and endothelial redox conditions. Disturbed flow can suppress endothelial protective programs and promote inflammatory activation. For example, endothelial uncoupling protein 2 (UCP2) is upregulated by unidirectional shear stress and KLF2, but suppressed by disturbed flow and inflammatory stimulation. Loss of UCP2 increases endothelial mitochondrial ROS, induces VCAM1, ICAM1, CCL2, and IL6 expression, enhances monocyte adhesion, and accelerates plaque formation under disturbed flow conditions [77]. In parallel, extracellular PRDX5 can function as a danger signal by activating TLR4/MyD88/NF‐κB and p38 signaling, thereby promoting endothelial inflammation, monocyte adhesion, macrophage accumulation, and plaque progression [59]. These findings indicate that chronic vascular inflammation is maintained by the convergence of abnormal flow, endothelial redox imbalance, lipid stress, and DAMP‐related signaling.

Macrophages serve as major amplification nodes within this chronic vascular network. In plaques, macrophages internalize modified lipids and cholesterol crystals, become foam cells, and provide a cellular platform for inflammasome activation, cytokine release, and pyroptotic amplification. Importantly, chronic inflammasome activity is shaped not only by persistent priming but also by immunometabolic and epigenetic regulation. Loss of mechanistic target of rapamycin complex 2 (mTORC2) signaling in macrophages can relieve inhibition of forkhead box O1 (FoxO1), enhance IL‐1β‐related inflammatory programs, and increase plaque complexity, necrotic core expansion, and macrophage cell death [79]. Similarly, in the context of tet methylcytosine dioxygenase 2 (TET2) clonal hematopoiesis, cholesterol loading and TET2 deficiency can cooperate to enhance JNK1 activation and promote BRCC3‐mediated deubiquitination of NLRP3, thereby aggravating inflammasome activation, NETosis, and atherosclerotic plaque progression [62]. These findings indicate that chronic low‐grade inflammation in atherosclerosis is maintained by integrated interactions among lipid stress, macrophage metabolism, epigenetic alterations, inflammasome regulation, and cell death programs.

In this vascular context, the primary role of mitophagy is to reduce the background burden of mitochondrial danger signals rather than to control a short‐lived inflammatory peak. When mitophagy is insufficient, damaged mitochondria persist in endothelial cells and macrophages, leading to sustained mtROS production, MMP loss, mPTP opening, calcium overload, and increased exposure of mitochondrial DAMPs. In ox‐LDL‐induced endothelial injury, PTEN upregulation suppresses AMPK–CREB–MFN2‐mediated mitophagy, thereby aggravating mitochondrial dysfunction and endothelial apoptosis. Conversely, inhibition of PTEN restores autophagy‐related marker expression, promotes mitochondria–lysosome fusion, and alleviates endothelial injury [159]. These findings indicate that, in atherosclerosis, mitophagy is protective mainly because it maintains long‐term mitochondrial turnover capacity and prevents continuous accumulation of mitochondrial danger signals that fuel endothelial activation and inflammatory amplification.

Nucleic acid‐sensing pathways further consolidate chronic vascular inflammation. In atherosclerosis, metabolic stress, lipotoxicity, mitochondrial injury, and mtDNA leakage can sustain cGAS–STING‐related inflammatory outputs. Activation of SUCNR1 can promote ER Ca^2^^+^ leakage and mitochondrial Ca^2^^+^ overload, thereby facilitating mtDNA release and downstream inflammatory activation [253]. Upregulation of IQGAP1 can increase ROS generation and mitochondrial depolarization, promote mtDNA release, and drive the cGAS–STING–NLRP3 axis and pyroptotic signaling [71]. In macrophages, ALDH2 deficiency and 4‐hydroxynonenal (4‐HNE) accumulation can stabilize cGAS protein and enhance cGAS–STING–TBK1/IRF3 signaling, thereby promoting proinflammatory macrophage polarization and atherosclerotic lesion progression [72]. Thus, in atherosclerosis, cGAS–STING does not function as an isolated DNA‐sensing pathway, but is embedded in a broader maintenance network composed of endothelial stress, mitochondrial injury, inflammasome activation, macrophage polarization, and impaired inflammatory resolution.

A similar low‐grade inflammatory logic is present in diabetic cardiomyopathy, although the dominant cellular source of danger signals shifts from the vascular wall to metabolically stressed cardiomyocytes. Under hyperglycemia and lipotoxicity, cardiomyocytes remain exposed to persistent mitochondrial injury, oxidative stress, impaired substrate metabolism, and metabolic inflexibility. These abnormalities continuously generate mtROS, promote mtDNA leakage, and sustain inflammatory signaling. Unlike atherosclerosis, where endothelial cells and macrophages form the core inflammatory unit, diabetic cardiomyopathy is characterized by the conversion of intracellular metabolic disequilibrium within cardiomyocytes into chronic inflammatory output. In this setting, TLR‐related priming, NLRP3 inflammasome activation, cGAS–STING signaling, apoptosis, and pyroptosis‐related programs may act together to drive myocardial injury, fibrosis, and contractile dysfunction.

In diabetic cardiomyopathy, mitophagy contributes to inflammatory threshold control by reducing damaged mitochondrial burden and restraining persistent danger signal output. H_2_S enhances USP8‐mediated Parkin deubiquitination, thereby facilitating Parkin recruitment and mitophagic activation. It also restores mitochondrial dynamics by suppressing DRP1/mitochondrial fission 1 protein (Fis1) and upregulating MFN2, ultimately alleviating mitochondrial injury and inflammatory amplification [163]. Metrnl activates the LKB1–AMPK–unc‐51‐like kinase 1 (ULK)1 axis and enhances autophagic flux. Through this process, it promotes STING dephosphorylation and TNF receptor‐associated factor 2 (TRAF2)‐mediated degradation, thereby reducing high glucose‐induced myocardial injury and persistent inflammatory output [74]. These findings suggest that, in chronic metabolic myocardial disease, mitophagy does not simply remove damaged mitochondria; it also raises the threshold for STING‐ and inflammasome‐related inflammatory activation by limiting the duration and magnitude of mitochondrial danger signal exposure.

Persistent DNA stress may further sustain inflammation in diabetic cardiomyopathy. Impaired double‐strand DNA break repair caused by BRG1 downregulation indicates that chronic inflammation in diabetic cardiomyopathy may arise not only from mitochondrial nucleic acid leakage but also from disruption of nuclear DNA homeostasis [254]. This expands the concept of nucleic acid‐driven inflammation beyond mtDNA alone and suggests that metabolic disease can maintain inflammatory signaling through combined mitochondrial and nuclear DNA stress. Therefore, the persistence of inflammation in diabetic cardiomyopathy is determined not by a single pathway, but by the combined state of MQC, DNA integrity, oxidative stress, and immunometabolic regulation.

The broader relevance of mitochondrial metabolic dysfunction is also supported by chronic nonischemic cardiomyopathy models. In doxorubicin‐associated dilated cardiomyopathy, SIRT3 downregulation, mitochondrial protein hyperacetylation, and sustained oxidative stress promote ventricular wall thinning, fibrosis, and functional decline. Enhancement of mitochondria‐localized SIRT3 reduces ROS accumulation, apoptosis, and adverse remodeling [78]. Although this model is not the central focus of vascular or metabolic disease, it supports a broader principle: persistent mitochondrial metabolic dysfunction can maintain low‐grade injury and remodeling programs even in the absence of an acute inflammatory burst.

From a cellular perspective, chronic vascular inflammation and metabolic myocardial inflammation share common network principles despite involving different dominant cell types. In atherosclerosis, endothelial cells regulate inflammatory entry and leukocyte recruitment, whereas macrophages serve as major amplifiers of lipid‐driven inflammation, inflammasome activation, pyroptosis, and danger signal persistence. In diabetic cardiomyopathy, cardiomyocytes become the principal source of metabolic stress, mitochondrial injury, and inflammatory output [225, 255, 256, 257]. The former is characterized by sustained interactions between structural vascular cells and immune cells, whereas the latter reflects the continuous conversion of intracellular metabolic imbalance within cardiomyocytes into inflammatory signaling. Despite these differences, both conditions are maintained by persistent danger signal input, insufficient MQC, impaired immunometabolic adaptation, and failure of inflammatory resolution.

At the threshold level, vascular and metabolic diseases exemplify a chronic inflammatory pattern characterized by low‐intensity persistent input, reduced activation threshold, and failure of resolution. The key pathological issue is not whether inflammatory signals reach an acute peak, but why relatively modest danger signal inputs can persist long enough to maintain TLR‐related priming, NLRP3 inflammasome activation, cGAS–STING signaling, and immunometabolic dysregulation. Insufficient mitophagy, redox imbalance, NAD^+^ depletion, disturbed AMPK/mTOR signaling, and impaired resolution mechanisms collectively elevate the background level of danger signals while weakening the system's capacity to terminate inflammatory responses. As a result, chronic vascular and metabolic diseases may progress through sustained low‐grade inflammatory maintenance rather than through overt inflammatory bursts.

Overall, atherosclerosis and diabetic cardiomyopathy represent a chronic low‐grade inflammatory organizational mode within CVDs. In this mode, the primary role of mitophagy is to reduce the background burden of mitochondrial danger signals and maintain the inflammatory activation threshold, while TLR–NF‐κB, NLRP3, cGAS–STING, and immunometabolic nodes determine why the inflammatory network remains active over prolonged periods. Thus, the central issue in vascular and metabolic disease is not transient activation of a single pathway, but the long‐term inability of the inflammatory network to return to homeostasis.

### 6.6 Maladaptive Inflammatory Maintenance in Stress Overload and Cardiomyopathy

Distinct from the acute high‐amplitude amplification observed in ischemic injury and the low‐grade chronic maintenance observed in vascular and metabolic diseases, prolonged pressure overload, toxic stress, and multiple forms of cardiomyopathy represent another mode of inflammatory network organization. In these settings, danger signals are not always generated as abrupt bursts, but more often arise from persistent inputs driven by chronic mechanical load, DNA damage, lipotoxicity, proteostatic disturbance, and organelle dysfunction. As sustained stress continues, MQC, immunometabolic adaptation, and inflammatory resolution mechanisms may become simultaneously disrupted, shifting inflammation from stress‐responsive regulation toward pathological maintenance and structural remodeling. Thus, the central issue is not whether a transient inflammatory peak occurs, but why the system progressively loses its capacity to buffer danger signals, constrain inflammatory amplification, and restore resolution.

In pressure overload‐induced HF, this maladaptive maintenance pattern is first reflected in a mismatch between mitophagy and metabolic adaptation. Chronic mechanical stress places cardiomyocytes under sustained high energetic demand and mitochondrial burden. When quality‐control responses are activated in a timely and proportionate manner, danger signal input can be reduced and the transition toward inflammatory maintenance may be delayed. For example, suppression of FUNDC1‐mediated mitophagy during HF progression can lead to ATP depletion, impaired oxidative phosphorylation, and ROS accumulation, whereas hypoxic adaptation can improve mitochondrial function and delay HF development by restoring FUNDC1‐, LC3‐II‐, AMPK‐, and PINK1‐related flux [157]. Similarly, moderate‐intensity resistance training enhances PINK1/Parkin‐dependent mitophagy through activation of the hypoxia‐inducible factor 1 alpha (HIF‐1α)/Parkin axis, thereby reducing oxidative stress and promoting ATP production [156]. Omentin1 upregulates MFN2 and OPA1 through SIRT3/FOXO3a while suppressing DRP1 phosphorylation, thereby enhancing damaged mitochondrial clearance and promoting mitochondrial fusion [155]. Together, these findings indicate that, in the early or compensatory phase of pressure overload, mitophagy is protective not only because it maintains mitochondrial abundance and energy supply, but also because it limits the sustained accumulation of ROS, mtDNA, and other mitochondrial danger signals.

Once stress persists and metabolic adaptation gradually decompensates, however, the dominant mechanism shifts from damage control to pathological maintenance. At this stage, mitochondrial danger signals are no longer merely upstream triggers, but become part of chronic self‐reinforcing loops with multiple inflammatory axes. Sustained ROS accumulation can enhance NF‐κB‐dependent transcriptional programs, maintaining cytokine, chemokine, and adhesion molecule expression. At the same time, ROS‐rich mitochondrial stress creates a permissive environment for NLRP3 inflammasome activation and prolonged release of IL‐1β, IL‐18, and pyroptosis‐related inflammatory outputs [38, 238]. Persistent exposure of nuclear DNA and mtDNA can further drive cGAS–STING‐related nucleic acid sensing. In TAC models, cytosolic leakage of nuclear DNA and mtDNA activates cGAS–STING signaling and promotes inflammation, apoptosis, fibrosis, and pathological hypertrophy. These findings suggest that, in pressure overload‐induced HF, cGAS–STING is not an isolated pathogenic axis, but part of a chronic inflammatory maintenance network shaped by NF‐κB‐related transcriptional persistence, NLRP3‐associated amplification, ROS accumulation, and mitochondrial gating failure.

Cardiotoxicity further illustrates how inflammatory maintenance can arise from bidirectional disequilibrium of MQC. In some toxic settings, the dominant problem is insufficient mitophagy. For example, in NASH‐related cardiotoxicity, key mitophagy‐related molecules such as PINK1/Parkin, BNIP3L, and FUNDC1 are markedly downregulated, making damaged mitochondria difficult to clear and thereby promoting oxidative stress, inflammation, and impaired energy metabolism. Bone marrow‐derived mesenchymal stem cells and their extracellular vesicles can restore the relevant mitophagic flux and improve metabolic and inflammatory status [168]. In other toxic settings, the problem is excessive or uncontrolled mitophagy. Ophiopogonin D’ markedly enhances PINK1/Parkin expression and increases the LC3‐II/LC3‐I ratio, accompanied by reduced MMP, aggravated oxidative stress, and increased apoptosis. Under these conditions, modulation of excessive flux with mdivi‐1 alleviates injury [167]. These observations indicate that the pathological role of mitophagy in cardiotoxicity is not determined simply by whether mitophagy is increased or decreased, but by whether mitochondrial clearance remains matched to injury burden and metabolic demand.

At the level of inflammatory network organization, cardiotoxicity is also a typical setting in which DNA damage, mitochondrial dysfunction, and inflammatory signaling operate in parallel. Anthracycline‐associated injury can generate conformationally altered mtDNA that is cooperatively sensed by Z‐DNA‐binding protein 1 (ZBP1) and cGAS, thereby amplifying cGAS–STING–IFN‐I signaling [64]. In cardiac endothelial cells, doxorubicin‐induced injury also activates the cGAS–STING–TBK1–IRF3–CD38 axis, leading to NAD^+^ depletion, mitochondrial dysfunction, endothelial injury, and apoptosis [65]. Cisplatin can promote cardiomyocyte injury by activating cGAS–STING‐associated inflammatory signaling and upregulating TNF‐α through activator protein‐1 (AP‐1) [258]. Therefore, the pathological significance of cGAS–STING in cardiotoxicity lies not only in DNA sensing itself, but in its embedding within a broader maintenance background characterized by persistent DNA damage input, NF‐κB/TNF‐α transcriptional amplification, NAD^+^ depletion, mitochondrial dysfunction, and insufficient inflammatory termination.

Multiple forms of cardiomyopathy further demonstrate the heterogeneity of this maladaptive maintenance mode. In dilated and septic cardiomyopathy, insufficient mitophagy is often prominent. Loss of BMAL1 impairs BNIP3‐dependent mitophagy and promotes dilated remodeling by enhancing abnormal mitochondrial fusion and mitochondrial dysfunction [162]. In LPS‐induced septic cardiomyopathy, downregulation of FUNDC1 and PINK1 suppresses autophagic flux and promotes ROS accumulation and cardiomyocyte death, whereas dual‐specificity phosphatase 1 (DUSP1) enhances damaged mitochondrial clearance and attenuates inflammatory injury by upregulating FUNDC1 and PINK1/Parkin through AMPK [161]. These findings indicate that insufficient mitophagy can first elevate danger signal input, while ROS and cellular stress further provide sustained amplification conditions for NF‐κB and inflammasome signaling.

By contrast, in hypertrophic cardiomyopathy or hypertrophy‐related stress settings, excessive quality‐control activation may also become maladaptive. Ionizing radiation markedly upregulates PINK1/Parkin and LC3, while SUMO2‐mediated SH3GLB1 SUMOylation strengthens its interaction with mitochondrial membrane proteins, thereby promoting excessive mitophagy, ROS generation, mitochondrial dysfunction, and cardiac hypertrophy [160]. In this context, the pathological core is not simply enhancement of mitophagy, but the loss of coordination between the quality‐control activity and metabolic reserve. When mitochondrial clearance becomes excessive or poorly matched to energetic demand, both mitochondrial population stability and inflammatory homeostasis may be disrupted.

At the cellular level, maladaptive inflammatory maintenance in these conditions reflects long‐term interactions among cardiomyocytes, immune cells, and fibroblasts. Cardiomyocytes are the principal recipients of chronic mechanical load, toxic injury, and metabolic stress, and they serve as major source of ROS, mtDNA, and metabolism‐related danger signals. Immune cells sustain the inflammatory environment through cytokine production, inflammasome activity, and pyroptosis‐related programs [26, 259]. Fibroblasts convert persistent inflammatory signaling into extracellular matrix deposition and structural remodeling [247]. Unlike the rapidly mobilized inflammatory network in acute ischemic injury, the key feature here is a pathological steady state generated by prolonged cellular crosstalk. Even without the peak intensity observed in acute injury, chronic inflammatory persistence can drive profound structural and functional deterioration.

From the threshold perspective, prolonged pressure overload, toxic stress, and cardiomyopathic remodeling reflect a pattern characterized by sustained input, adaptive failure, and impaired resolution [31, 257, 260]. The key issue is not whether danger signals can rapidly exceed a high activation threshold, but whether the system progressively loses its capacity to buffer danger signal‐driven activation and bring it to resolution. Insufficient or dysregulated mitophagy continuously increases the basal burden of ROS, mtDNA, and oxidized lipids. In parallel, NAD^+^ depletion, AMPK/mTOR imbalance, and weakened antioxidant defenses compromise negative feedback and resolution mechanisms [16, 240, 243]. As a result, TLR–NF‐κB, NLRP3, and cGAS–STING may not necessarily reach maximal activation at any single moment, yet they may coexist over long periods at low‐to‐moderate intensity and collectively maintain pathological remodeling.

Overall, pressure overload‐induced HF, cardiotoxicity, and multiple forms of cardiomyopathy define a maladaptive inflammatory maintenance mode within CVDs. In this mode, the central problem is no longer short‐term high‐intensity amplification or simple low‐grade persistence, but the combined failure of MQC, immunometabolic adaptation, and inflammatory resolution under sustained stress. The relationship between mitophagy and inflammation should therefore not be interpreted as simple enhancement or inhibition of a single pathway. Rather, once mitochondrial homeostasis can no longer be maintained, danger signal input, inflammatory amplification, and inflammatory resolution failure become mutually reinforcing, driving multiple inflammatory modules into a chronic maintenance state that is difficult to reverse.

Taken together, the stage‐cell‐threshold framework provides an integrated explanation for the heterogeneity of cardiovascular inflammation across different disease settings. Acute ischemic injury is dominated by abrupt danger signal release, rapid coupling of inflammatory modules, and high‐intensity threshold crossing. Vascular and metabolic diseases more commonly exhibit persistent low‐grade danger signal input, chronic inflammatory maintenance, and impaired resolution. Pressure overload, toxic injury, and cardiomyopathy further represent a maladaptive maintenance mode in which MQC failure, immunometabolic maladaptation, and defective inflammatory termination become mutually reinforcing. These patterns indicate that inflammatory outcomes in CVDs are not determined by the activation of a single pathway alone, but by the coordinated state of disease stage, dominant cellular contributors, mitochondrial danger signal burden, and threshold‐gating capacity.

Within this framework, mitophagy should not be interpreted as uniformly protective or uniformly harmful. Rather, it functions as an upstream gating system that regulates the accumulation of mitochondrial DAMPs and determines whether inflammatory sensing remains within an adaptive range or progresses toward pathological amplification. TLR–NF‐κB signaling, NLRP3 inflammasome activation, cGAS–STING‐mediated nucleic acid sensing, and immunometabolic regulation each contribute to this network at different functional levels. Therefore, the apparent differences in inflammatory outcomes across CVDs may reflect distinct positions within the same multidimensional regulatory framework. This perspective also provides a rationale for therapeutic strategies that move beyond uniform suppression of single inflammatory pathways and instead consider disease stage, key cellular targets, danger signal burden, and threshold status.

## 7 Network‐Guided Therapeutic Strategies and Translational Perspectives

The foregoing analysis makes it clear that cardiovascular inflammation is not driven by a single pathological pathway, but arises from an interconnected network involving danger signal input, inflammatory priming, signal amplification, nucleic acid sensing, immunometabolic regulation, and resolution failure. Accordingly, therapeutic strategies targeting cardiovascular inflammation should move beyond uniform suppression of individual inflammatory mediators and instead consider how different network layers are organized across disease stages, cellular contexts, and threshold states. In particular, the same inflammatory module may exert protective, neutral, or detrimental effects depending on timing, disease background, dominant cell type, and the burden of mitochondrial danger signals. Therefore, effective intervention should aim not only to inhibit inflammatory outputs, but also to reduce pathological danger signal input, restrain maladaptive amplification, restore threshold control, and promote inflammatory resolution.

**FIGURE 5:**
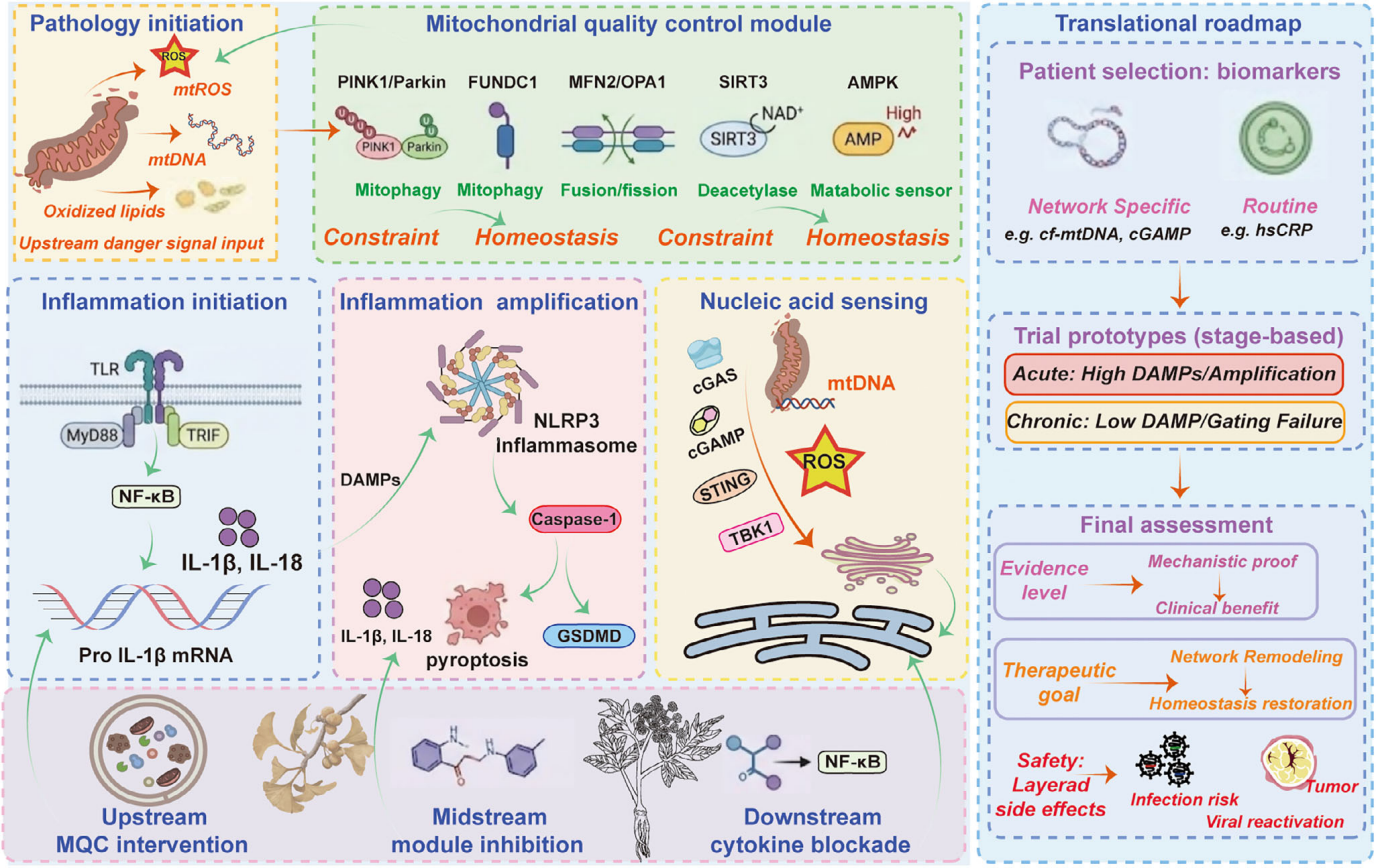
Network‐guided therapeutic framework and translational roadmap for cardiovascular inflammation. Therapeutic intervention in cardiovascular inflammation can be stratified across multiple network layers, including upstream danger signal control through MQC, intermediate suppression of inflammatory priming, inflammasome amplification, and nucleic acid sensing, and higher order restoration of immunometabolic gating. The translational pathway further requires biomarker‐based patient selection, stage‐informed trial prototypes, integrated efficacy assessment, and mechanism‐informed safety monitoring. This framework highlights that effective therapy should focus on layered and context‐dependent regulation of inflammatory networks rather than indiscriminate inhibition of single pathways.

From this network‐guided perspective, therapeutic targets can be broadly organized into several functional layers. Upstream strategies focus on limiting mitochondrial DAMP generation through restoration of MQC, mitophagy, mitochondrial dynamics, antioxidant defense, and metabolic adaptation. Intermediate strategies target inflammatory priming, inflammasome‐mediated amplification, and aberrant nucleic acid sensing, including TLR–NF‐κB, NLRP3, and cGAS–STING‐related pathways. Higher order strategies aim to restore immunometabolic gating and threshold stability through modulation of ROS, NAD^+^–sirtuin signaling, AMPK/mTOR balance, and resolution mechanisms. As illustrated in Figure 5, this network‐guided therapeutic framework can be organized across multiple interventional layers, including upstream danger signal control through MQC, intermediate targeting of inflammatory priming, inflammasome amplification, and nucleic acid sensing, higher order restoration of immunometabolic gating, and translational evaluation based on patient selection, efficacy assessment, and safety monitoring.

However, although numerous preclinical studies support the potential of targeting mitochondrial danger signaling and inflammatory networks, the translational interpretation of these findings requires caution. Many current studies rely on small animal models, short observation windows, surrogate endpoints, preventive dosing designs, and limited reporting of randomization or blinding [261, 262]. In addition, some natural products and multicomponent formulations are administered at relatively high doses, raising questions about extract standardization, exposure, human‐equivalent dosing, therapeutic index, and clinical feasibility [263]. Therefore, preclinical evidence in this field should be interpreted primarily as mechanistic and proof‐of‐concept support rather than as direct evidence of clinical applicability.

### 7.1 Limiting Upstream Danger Signal Input via Mitochondrial Quality Control

From the hierarchical organization of the inflammatory network, MQC represents one of the most upstream and strategically important therapeutic entry points [264]. Mitochondria are not only central organelles for energy metabolism, but also major sources of endogenous danger signals. When mitophagy, mitochondrial dynamics, biogenesis or antioxidant defense becomes insufficient, mitochondrial‐derived signals such as mtROS, mtDNA, oxidized lipids, and CL may accumulate and become aberrantly exposed. These signals provide shared upstream inputs for several inflammatory modules, including TLR–NF‐κB signaling, NLRP3 inflammasome activation, and cGAS–STING‐mediated nucleic acid sensing. Therefore, reducing mitochondrial danger signal burden at its source may provide a more fundamental strategy than suppressing downstream inflammatory outputs alone.

At this upstream level, restoring appropriately regulated mitophagy is one of the most representative approaches. In models of myocardial infarction, atherosclerosis, diabetic cardiomyopathy, and HF, modulation of quality‐control programs involving PINK1/Parkin, FUNDC1, MFN2/OPA1, or TFEB transient receptor potential mucolipin 1/(TRPML1) has been associated with reduced accumulation of damaged mitochondria, lower ROS production, improved MMP, improved energy metabolism, and attenuated inflammatory activation [265, 266]. The therapeutic value of these interventions does not lie simply in enhancing autophagy itself, but in restoring the recognition, trafficking, and lysosomal degradation of dysfunctional mitochondria before mitochondrial imbalance progresses into sustained inflammatory amplification [88, 264]. In this sense, MQC‐targeted intervention can influence multiple inflammatory modules simultaneously because it acts on a shared upstream source of danger signals.

However, MQC‐based therapy should not be understood as unidirectional enhancement of mitophagy. In certain pathological contexts, excessive or prolonged mitophagy may become maladaptive. This is particularly relevant in chronic pressure overload, selected forms of drug‐induced cardiotoxicity, and specific cardiomyopathies, where excessive mitochondrial clearance may deplete compensatory mitochondrial populations, reduce energy supply, and weaken adaptive stress responses. Therefore, therapeutic regulation of mitophagy should aim to restore a dynamic balance among injury burden, mitochondrial clearance, mitochondrial renewal, and metabolic demand [92, 267, 268]. The goal is not to maximize mitophagic activity, but to reduce pathological danger signal input while preserving sufficient mitochondrial reserve for cellular adaptation.

Beyond mitophagy itself, several immunometabolic and mitochondrial regulatory nodes also contribute to upstream danger signal control. SIRT3, AMPK, FOXO1/FOXO3a, and MFN2/OPA1 influence mitochondrial homeostasis by regulating antioxidant capacity, fusion–fission balance, autophagic flux, and metabolic flexibility. By stabilizing these processes, MQC‐directed strategies may reduce mtROS generation, limit mtDNA leakage, preserve mitochondrial membrane integrity, and increase the threshold required for inflammatory activation. Thus, the significance of MQC in a network‐guided therapeutic framework extends beyond mitochondrial protection alone. It serves as a critical interface through which restoration of metabolic homeostasis can reduce upstream inflammatory input and reshape the broader inflammatory network.

### 7.2 Targeting Inflammatory Priming, Amplification, and Nucleic Acid Sensing

Although limiting upstream danger signal input provides an important starting point for network‐guided intervention, restoration of MQC alone may be insufficient once inflammatory amplification loops have already been established. This is particularly relevant in acute injury, chronic low‐grade inflammation, and maladaptive remodeling, where the inflammatory network is sustained by several functional layers acting in parallel. Therefore, effective intervention also requires stratified modulation of inflammatory priming, signal amplification, and aberrant nucleic acid sensing. The key is not to block innate immune responses indiscriminately, but to identify the dominant pathogenic layer within a given disease context and apply more precisely matched regulatory strategies.

The inflammatory priming layer is mainly organized around danger‐sensing systems and downstream transcriptional programs. Among these, the TLR–MyD88/TRIF–NF‐κB axis represents a central module through which endogenous DAMPs induce cytokines, chemokines, adhesion molecules, NLRP3, and pro‐IL‐1β/pro‐IL‐18 expression [26]. Therapeutically, targeting this layer does not mean completely abolishing innate immune recognition, which remains necessary for host defense, debris clearance, and tissue repair. Rather, its purpose is to reduce excessive system sensitivity to persistent danger signals and prevent the inflammatory network from being easily pushed into a highly amplified state. This strategy may be especially relevant during the initiation phase of ischemia–reperfusion injury and in chronic vascular inflammation, where sustained TLR–NF‐κB activity contributes to cytokine production, leukocyte recruitment, endothelial activation, and inflammatory priming.

The amplification layer is mainly represented by inflammasome‐related execution programs, especially the NLRP3 inflammasome. Once priming has occurred, mitochondrial dysfunction, ROS accumulation, ion flux disturbance, lysosomal injury, and mtDNA exposure can provide activation signals that drive inflammasome assembly, caspase‐1 activation, IL‐1β/IL‐18 maturation, and pyroptotic cell death. Because this layer converts upstream stress signals into cytokine release and inflammatory cell death, it represents a more downstream but highly influential point of therapeutic intervention. In pathological settings such as acute ischemic injury, atherosclerotic plaque progression, pressure overload, and HF, excessive NLRP3 activation can promote tissue injury, fibrotic remodeling, endothelial dysfunction, and plaque instability. Therefore, therapeutic modulation of this layer should aim to interrupt maladaptive inflammatory execution while preserving sufficient immune activity required for clearance and repair.

Nucleic acid sensing represents another critical intervention layer, especially when mitochondrial or nuclear DNA stress becomes persistent. The cGAS–STING pathway links aberrant cytosolic DNA exposure to IFN‐I responses, NF‐κB‐related transcriptional programs, and inflammatory amplification [269]. Unlike TLR‐associated priming and NLRP3‐mediated execution, the distinctive feature of this layer is its sensitivity to disrupted DNA compartmentalization and nucleic acid homeostasis. In acute injury, transient nucleic acid sensing may contribute to recognition and clearance of damaged tissue. By contrast, in chronic metabolic stress, cardiotoxicity, pressure overload, and remodeling‐related diseases, persistent DNA leakage and sustained STING activation may reinforce inflammation, metabolic deterioration, endothelial injury, fibrosis, and adverse remodeling. Therefore, targeting this layer should not be understood simply as broad STING inhibition, but as context‐dependent correction of pathological nucleic acid‐sensing output.

From a network perspective, the therapeutic value of modulating cGAS–STING lies in limiting the persistent conversion of aberrantly exposed nucleic acids into proinflammatory and interferon‐related programs, while also reducing its crosstalk with NLRP3, NF‐κB, and immunometabolic dysregulation. At the same time, alternative nucleic acid‐sensing platforms such as TLR9, MAVS, and NLRX1 indicate that nucleic acid recognition is not organized through a single pathway, but through a multiplatform and context‐dependent system [270]. Therapeutic intervention at this level should therefore aim to correct pathological DNA‐ or RNA‐sensing persistence while preserving the physiological sensing capacity required for immune surveillance and tissue repair.

Overall, targeting inflammatory priming, signal amplification, and nucleic acid sensing offers a layered strategy for reshaping established inflammatory networks in CVDs. Suppression of excessive priming lowers the sensitivity of the system to danger signals, while control of inflammasome‐related amplification limits inflammatory execution and tissue destruction. Modulating nucleic acid sensing further prevents aberrant DNA or RNA exposure from being converted into persistent inflammatory output. Nevertheless, these approaches are unlikely to produce durable benefit unless they are coupled with upstream reduction of danger signal input and downstream restoration of inflammatory resolution. Effective intervention should therefore move beyond isolated inhibition of a single inflammatory module and instead coordinate the regulation of danger signal burden, inflammatory amplification, and nucleic acid‐sensing output.

### 7.3 Restoring Immunometabolic Gating and Threshold Control

Although limiting danger signal input and modulating inflammatory priming, amplification, and nucleic acid sensing provide important foundations for therapeutic intervention, the final outcome of cardiovascular inflammation is not determined solely by whether these modules are activated. It also depends on whether the system retains sufficient capacity to maintain threshold stability, buffer inflammatory stress, and prevent signal spillover [271]. In this sense, immunometabolic nodes and redox regulatory systems are not merely background conditions for inflammation, but critical gating layers that determine whether inflammatory activation remains within an adaptive range or progresses toward pathological maintenance. Therefore, restoration of immunometabolic gating and threshold control should be regarded as a higher order objective in network‐guided therapeutic strategies.

ROS represent one of the most important regulators of this gating layer. As both a source of danger signals and a driver of signal amplification, ROS can aggravate biomolecular damage, promote mitochondrial dysfunction, enhance NF‐κB‐dependent inflammatory transcription, and facilitate NLRP3 inflammasome activation [38]. Therefore, controlling ROS should not be understood as conventional antioxidant intervention. More importantly, it can be viewed as a strategy to lower the gain of the inflammatory network, reduce the probability of self‐amplifying inflammatory loops, and restore the buffering capacity of cardiovascular cells under stress.

The NAD^+^–sirtuin axis provides another key level of threshold regulation. Declining NAD^+^ availability and impaired sirtuin function weaken mitochondrial antioxidant capacity, reduce metabolic adaptability, and make it more difficult for tissues to engage effective inflammatory resolution mechanisms. As a result, the inflammatory network may persist chronically even at relatively low levels of danger signal input [243]. From this perspective, enhancing NAD^+^ availability and restoring the function of deacetylases such as SIRT3 should not be regarded as indirect modulation of a single inflammatory pathway. Rather, these approaches may help rebuild the metabolic and redox conditions required for inflammatory threshold control.

The energy‐sensing system centered on AMPK and mTOR further determines whether inflammatory responses are constrained and buffered or instead remain persistently amplified [272]. AMPK activation is generally associated with enhanced autophagy, improved mitochondrial homeostasis, and suppression of excessive inflammation, whereas excessive mTOR activity is more likely to support proinflammatory transcriptional programs and metabolic disequilibrium. Importantly, AMPK/mTOR signaling does not simply determine whether a specific inflammatory module is switched on or off. Instead, it shapes whether cells facing persistent danger signals maintain homeostatic adaptation or enter a state of chronic inflammatory maintenance.

From a therapeutic perspective, restoring immunometabolic gating can act simultaneously on three levels: danger signal input, inflammatory amplification, and inflammatory termination. By lowering ROS burden, restoring mitochondrial metabolic adaptation, and improving autophagic flux, upstream danger signal input can be reduced. By limiting sustained activation of NF‐κB, NLRP3, and cGAS–STING, the amplification capacity of the inflammatory network can be restrained. By strengthening metabolic buffering and resolution capacity, the system may be less likely to remain locked in a pathological maintenance state. For this reason, restoring immunometabolic gating is more consistent with the threshold dependent and dynamically regulated nature of cardiovascular inflammation than complete suppression of any single inflammatory pathway.

### 7.4 Stage‐, Cell‐, and Context‐Informed Intervention Strategies

Within the cardiovascular inflammatory network, the dominant pathogenic layer varies across disease stages, cellular contexts, and pathological backgrounds [7, 31, 247]. Therefore, effective therapeutic strategies cannot be defined by a uniform anti‐inflammatory principle alone. Based on the stage‐cell‐threshold perspective discussed above, intervention should first consider the temporal state of disease, then the dominant pathological context, and finally the major cellular contributors that sustain the inflammatory network. In other words, treatment should not follow a one‐size‐fits‐all model, but should identify whether the disease is primarily driven by acute danger signal release, chronic low‐grade inflammatory maintenance, or maladaptive remodeling under sustained stress.

In acute ischemic injury, the inflammatory network usually presents as a rapid amplification pattern driven by high‐intensity danger signal release within a short time window. Accordingly, the main therapeutic objective is to rapidly reduce danger signal input and restrain excessive inflammatory amplification [222, 247]. In this context, restoring MQC, reducing mtROS and mtDNA leakage, suppressing excessive inflammasome activation, and limiting aberrant nucleic acid‐sensing output may all carry therapeutic value. At the same time, the necessary capacity for damage recognition, debris clearance, and repair initiation should be preserved. This means that intervention in acute ischemic injury should focus on narrowing excessive inflammatory peaks rather than abolishing inflammation completely.

In chronic vascular and metabolic diseases, the therapeutic logic is different. Here, the major problem is not an abrupt inflammatory burst, but persistent low‐grade danger signal input, reduced inflammatory threshold, and failure of resolution [13, 259]. Therefore, intervention should aim to lower the background burden of mitochondrial and metabolic stress while restoring the ability of tissues to terminate inflammatory responses. Strategies that improve endothelial mitochondrial function, reduce ox‐LDL‐ or hyperglycemia‐induced mtROS production, restore mitophagic flux, and stabilize immunometabolic gating may be particularly relevant. In these settings, long‐term control of danger signal burden may be more important than short‐term suppression of a single inflammatory effector.

In pressure overload, toxic injury, and cardiomyopathic remodeling, inflammation often persists because MQC failure, immunometabolic maladaptation, and defective resolution reinforce one another over time [31]. Therapeutic intervention in this setting should therefore focus on restoring adaptive capacity rather than simply suppressing inflammatory mediators. Depending on the disease stage, this may involve enhancing insufficient mitophagy, restraining excessive mitochondrial clearance, restoring NAD^+^–sirtuin activity, improving AMPK/mTOR balance, or reducing chronic cGAS–STING and NLRP3 activation. The key is to re‐establish coordination among mitochondrial turnover, metabolic reserve, and inflammatory termination so that the system can exit a pathological maintenance state.

Cell type specificity should also be interpreted together with disease stage and pathological context. In acute ischemic injury, cardiomyocytes are often the initial source of mitochondrial DAMPs; therefore, early intervention may prioritize preservation of mitochondrial homeostasis, limitation of mtDNA leakage, and protection of contractile and metabolic function [143]. During reperfusion and early inflammatory amplification, endothelial cells and recruited neutrophils or macrophages become more important therapeutic targets, and strategies aimed at preserving endothelial barrier function, reducing adhesion molecule expression, limiting leukocyte recruitment, and restraining inflammasome activation may be more relevant. In chronic vascular and metabolic diseases, endothelial cells and macrophages are usually central to sustained low‐grade inflammation [273, 274]; thus, intervention may focus on reducing oxidative stress, improving lipid handling, limiting foam cell formation, suppressing pyroptosis, and correcting inflammatory polarization. In the remodeling phase of pressure overload, cardiotoxicity, or cardiomyopathy, fibroblasts and stressed cardiomyocytes become increasingly important because persistent inflammatory signaling is progressively translated into extracellular matrix deposition, fibrosis, and contractile dysfunction [275]. Therefore, the same molecular target may require different therapeutic interpretation depending not only on the dominant cell type, but also on whether the disease is in an acute injury phase, chronic inflammatory maintenance phase, or maladaptive remodeling phase.

This context‐informed view also helps interpret apparently conflicting preclinical findings. For example, mitophagy enhancement may be protective when damaged mitochondria are the main source of danger signals, but excessive mitophagy may be harmful when mitochondrial reserve is already compromised. Similarly, cGAS–STING inhibition may be beneficial when persistent DNA sensing drives chronic inflammation, but indiscriminate suppression of nucleic acid sensing could interfere with damage recognition and immune surveillance. Therefore, the therapeutic value of a given target should be evaluated according to disease stage, cell type, signal intensity, and threshold status, rather than by assuming that the same pathway has a fixed function across all CVDs.

Overall, stage‐, cell‐, and context‐informed intervention provides a practical extension of the network‐guided therapeutic framework. It shifts the focus from universal anti‐inflammatory suppression to precision regulation of the dominant pathological layer in each disease setting. Such an approach may improve the therapeutic window of inflammatory modulation by reducing maladaptive amplification while preserving necessary inflammatory functions required for clearance, repair, and resolution.

### 7.5 Preclinical Evidence: Strengths, Gaps, and Model Limitations

Preclinical pharmacological studies have provided substantial support for network‐guided inflammatory intervention in CVDs. As summarized in Table 3, interventions across multiple disease models, including MI, MI/RI, AS, DCM, and cardiotoxicity, have been associated with improvements in cardiac function, reductions in proinflammatory cytokines and ROS levels, attenuation of fibrosis, mitigation of mitochondrial injury, and modulation of key inflammatory pathways. These findings suggest that therapeutic strategies targeting MQC, inflammatory priming, inflammasome activation, nucleic acid sensing, and immunometabolic regulation exhibit a high degree of mechanistic convergence. Therefore, current preclinical evidence is valuable because it shows that major nodes of the cardiovascular inflammatory network are pharmacologically modifiable.

**TABLE 3:** Representative preclinical pharmacological interventions targeting inflammatory network modules in cardiovascular disease.

Intervention	Experimental model	Doses/regimen	Inflammatory module/role	Key mechanism	Main outcome	Refs.
QLQX	**In vivo**: LAD ligation‐induced MI in male FVB/NJ mice **In vitro**: Hypoxia‐treated H9c2 cells	**In vivo**: 0.25 g/kg/day, i.g., 4 weeks **In vitro**: 0.5 mg/mL, 12 h	Mitophagy/danger signal control	Activated PINK1/Parkin‐mediated mitophagy, with ROS ↓, MMP ↑, and Bax/Bcl‐2 imbalance ↓	Mitochondrial damage ↓; Survival ↑, apoptosis ↓, LVEF and LVFS ↑, INF/AAR value ↓, mitochondrial damage ↓	[276]
Ginsenoside Rb1	**In vivo**: AMI induced by LAD ligation in ICR male mice **In vitro**: OGD‐injured H9c2 cells	**In vivo**: 6 mg/kg, i.p., after 20 min of CAL **In vitro**: 10 µM during hypoxia for 6 h	Mitophagy/danger signal control	Promoted AMPKα‐dependent mitophagy, with FUNDC1, PINK1, Parkin, LC3II/LC3I ↑ and p62 ↓; Improved mitochondrial morphology and function	Infarct size ↓, CRP, cTn‐I, and TNF‐α ↓, myocardial fibrosis ↓, mitochondrial damage ↓, cell viability ↑, LDH release ↓, MMP ↑	[277]
TYTZD	**In vivo**: Myocardial ischemia–reperfusion no‐reflow rat model induced by LAD ligation for 45 min and 24 h reperfusion in SD rats	**In vivo**: 3.6, 7.2, and 14.4 g/kg/day, i.g., for 7 days before modeling	cGAS–STING /nucleic acid sensing and inflammatory output	Suppressed cGAS–STING pathway activation and downstream NF‐κB expression, with dsDNA ↓ and serum IL‐6/IL‐1β/TNF‐α ↓, thereby attenuating inflammatory response	No‐reflow area ↓, LVEF and LVFS ↑, microcirculatory peak time ↓, CK, CK‐MB, LDH, cTnI, and TNNT2 ↓, myocardial injury ↓, inflammatory infiltration ↓	[278]
Scutellarin	**In vivo**: MI/RI induced by LAD ligation for 30 min followed by 24 h reperfusion in male C57BL/6 mice **In vitro**: H/R‐injured H9c2 cells	**In vivo**: 20 mg/kg, i.p., for 7 days before I/R **In vitro**: 25, 50, and 100 µM for 48 h	cGAS–STING /nucleic acid sensing	Inhibited cGAS–STING‐associated apoptotic signaling, with cleaved Caspase‐3 ↓ and Bcl‐2/Bax ratio ↑	LVEF ↑, LVFS ↑, infarct size ↓, apoptosis ↓, cell viability ↑, myocardial injury ↓	[279]
YMG	**In vivo**: HFD and vitamin D3‐induced atherosclerosis in male SD rats **In vitro**: Ang II‐injured HUVECs	**In vivo**: 4.45, 8.9, and 17.8 g/kg/day, i.g., for 21 weeks **In vitro**: 10% medicated serum for 24 h after 700 nM Ang II for 24 h	Mitophagy/danger signal control	Activated PINK1–Mfn2–Parkin‐mediated mitophagy, with miR‐125a‐5p‐related signaling modulation	Atherosclerotic plaque area ↓, endothelial injury ↓, mitophagy inhibition ↓, LDL‐C, TC and TG ↓, hs‐CRP ↓, ET ↓, HUVEC viability ↑	[280]
Icariin	**In vivo**: HFD‐fed ApoE^−/−^ male mice with atherosclerosis **In vitro**: ox‐LDL‐injured HUVECs	**In vivo**: 40 or 80 mg/kg/day, i.g., from Week 4 for 8 weeks **In vitro**: 12.5, 25, and 50 µg/mL pretreatment for 2 h, followed by 20 µg/mL ox‐LDL for 24 h	Mitophagy/danger signal control	Promoted TFEB/TRPML1‐associated autophagy‐mitophagy, with ROS and Fe^2^^+^ ↓, MMP ↑	Plaque area ↓, collagen fiber area ↓, TC ↓, TG ↓, IL‐6 ↓, TNF‐α ↓, cell viability ↑, ferroptosis ↓, mitochondrial dysfunction ↓	[281]
Urolithin A	**In vivo**: STZ‐induced diabetic C57BL/6J mice with insulin‐induced severe hypoglycemia	**In vivo**: 5 mg/kg, i.p., at 24 and 1 h before hypoglycemic intervention	Mitophagy/danger signal control	Restored PINK1/Parkin‐dependent mitophagy, with mito‐ROS ↓, TMRE and ATP ↑	LVEF ↑, LVFS ↑, serum TnI ↓, BNP ↓, myocardial injury ↓, inflammatory infiltration ↓, mitochondrial damage ↓	[282]
Nicorandil	**In vivo**: Long‐term diabetic cardiomyopathy in db/db mice **In vitro**: HCMECs were treated with 25 mM glucose and 500 µM FFAs for 72 h	**In vivo**: 10 mg/kg/day, i.g., 24 weeks **In vitro**: 100 µM, 72 h	Mitophagy/danger signal control	Promoted AMPKα1–Parkin‐dependent mitophagy and restrained ACSL4‐associated ferroptotic signaling	LVEF, LVFS and E/A ratio ↑, BNP ↓, cardiac hypertrophy ↓, fibrosis ↓, microvascular perfusion ↑, endothelial leakage ↓, ferroptosis ↓, cell viability ↑, cardiac microvascular dysfunction ↓	[283]
GLXB	**In vivo**: HFD‐induced atherosclerosis in ApoE^−/−^ mice	**In vivo**: 3, 6 and 12 g/kg/day, i.g., for 4 weeks	cGAS–STING /nucleic acid sensing	Inhibited LOX‐1‐mediated cGAS–STING activation and NCOA4‐associated ferritinophagy, with GPX4 ↑, SLC7A11 ↑, TFR1 ↓, ALOX15 ↓	Plaque area ↓, intima‐media thickness ↓, TNF‐α, IL‐1β, MDA and LPO ↓; GSH and SOD ↑, mitochondrial damage ↓	[284]
Baicalin	**In vivo**: STZ and HFD‐induced diabetic cardiomyopathy in SD rats	**In vivo**: 120 mg/kg/day, i.g., for 3 weeks	cGAS–STING /nucleic acid sensing	Inhibited cGAS–STING signaling, with cGAS, STING, CRP, IL‐18 and TNF‐α ↓; Effect was attenuated by cGAS overexpression	FBG ↓, EF and IVRT ↑, apoptosis ↓, CK‐MB, LDH and cTnI ↓, myocardial inflammation ↓	[285]
Tetrandrine	**In vivo**: HFD‐fed ApoE^−/−^ mice with atherosclerosis **In vitro**: cGAMP or ox‐LDL treatment of macrophages	**In vivo**: 20 mg/kg/day, i.g., for 8 weeks **In vitro**: 5, 10, and 20 µM pretreatment for 1 h	cGAS–STING /nucleic acid sensing	Inhibited STING/TBK1/NF‐κB signaling, with p65 nuclear translocation ↓ and proinflammatory gene expression ↓	Plaque area ↓, macrophage, monocyte and neutrophil infiltration ↓; TNF‐α, IL‐6 and IL‐1β ↓, fibrosis ↓, foam cell formation ↓	[286]
Irisin	**In vivo**: HFD and STZ‐induced diabetic cardiomyopathy in C57BL/6J mice **In vitro**: H9c2 cells treated with high glucose and high fat	**In vivo**: 0.5 µg/g/day, i.v., for successive 4 weeks **In vitro**: Irisin pretreatment for 4 h	cGAS–STING /nucleic acid sensing	Upregulated MITOL and suppressed cGAS–STING‐associated GSDMD‐mediated pyroptosis, with NLRP3, caspase‐1, IL‐1β, and GSDMD ↓	LVEF and LVFS ↑, LVIDd and LVIDs ↓, fibrosis, hypertrophy, apoptosis and pyroptosis ↓, MMP ↑	[287]
Canagliflozin	**In vivo**: ISO‐induced cardiac remodeling in adult male C57BL/6 mice	**In vivo**: 20 mg/kg/day, s.c., for 14 days	Mitophagy/danger signal control	Inhibited excessive AMPK/PINK1/Parkin‐mediated mitophagy, with p‐AMPK, PINK1, Parkin, p‐Parkin, LC3B‐II, and Beclin‐1 ↓, p62 ↑,	LVEF and LVFS ↑, HW/BW ↓, cardiomyocyte hypertrophy ↓, fibrosis ↓, apoptosis ↓, ROS ↓, mitochondrial damage ↓, cardiac remodeling ↓	[288]
SFYX	**In vivo**: Heart failure postmyocardial infarction induced by LAD ligation in SD rats **In vitro**: Hypoxia‐treated H9c2 cells	**In vivo**: 1.76, 5.28, and 8.8 g/kg/day, i.g., for 4 weeks; **In vitro**: drug‐containing serum for 24, 48, and 72 h	Mitophagy/danger signal control	Activated SIRT3/FOXO1‐mediated mitophagy, with Ac‐FOXO1 ↓, BNIP3, ATG5, ATG7 and LC3B‐II ↑, p62 ↓, ROS ↓, ATP/ADP ↑, NAD^+^/NADH ↑	LVEF and LVFS ↑, LVEDD ↓, infarct size ↓, BNP, ANP, ALD, fibrosis and apoptosis ↓, MMP ↑	[289]
HDD	**In vivo**: TAC‐induced heart failure in SD rats **In vitro**: 1 µM Ang II‐treated H9c2 cardiomyocytes	**In vivo**: 4.69 and 9.38 g/kg/day, i.g., for 8 weeks **In vitro**: 200 µg/mL for 24 h	Mitophagy/danger signal control	Activated AMPKα2–PINK1/Parkin mediated mitophagy, partly through reducing PAT‐derived EV miR‐27a‐3p, with p62 and ROS ↓	MMP, LVEF and LVFS ↑, LVIDd and LVIDs ↓; BNP, HW/TL, hypertrophy, apoptosis and fibrosis ↓; TNF‐α, IL‐1β, IL‐6 and IL‐18 ↓	[290]
MYSD	**In vivo**: HFD and L‐NAME‐induced HFpEF in C57BL/6 mice	**In vivo**: 28.3580 and 56.7150 g/kg/day, i.g., for 8 weeks	cGAS–STING /nucleic acid sensing	Inhibited cGAS–STING signaling, with cGAS ↓, STING ↓, and fibrosis‐associated TIMP‐1 ↓, MMP‐9 ↓	Swimming time ↑; E/A and BNP ↓, collagen area ↓, cardiomyocyte cross‐sectional area ↓, myocardial fibrosis ↓, myocardial hypertrophy ↓	[291]
XKG	**In vivo**: Doxorubicin hydrochloride‐induced chronic heart failure in SD rats	**In vivo**: 5.4 g/kg/day, i.g., for 4 weeks	cGAS–STING /nucleic acid sensing	Inhibited cGAS–STING‐associated inflammatory signaling, with TFAM ↑, cGAS, STING, and IL‐6 ↓	LVEF and LVFS ↑, LVIDd and LVIDs ↓; IL‐1β and IL‐6 ↓, inflammatory infiltration ↓, myocardial edema ↓, mitochondrial damage ↓	[292]
QDWX	**In vivo**: Doxorubicin‐induced cardiotoxicity in male C57BL/6J mice	**In vivo**: 1.3, 2.6, and 5.2 g/kg/day, i.g., for 4 weeks after doxorubicin modeling	cGAS–STING /nucleic acid sensing and inflammatory output	Inhibited cGAS–STING /NF‐κB signaling, with p‐STING/STING, p‐TBK1, p‐IRF3, p‐NF‐κB, IL‐6, IL‐1β, and TNF‐α **↓**	LVEF and LVFS ↑, LDH and TnI ↓; perivascular fibrosis ↓, inflammatory infiltration ↓, myocardial injury ↓	[293]
Geniposide	**In vivo**: MI/RI induced by LAD occlusion for 30 min followed by 120 min reperfusion in SD rats	**In vivo**: 50, 100, and 150 mg/kg/day, i.p., for 7 days before MI/RI	TLR–NF‐κB/inflammatory priming	Inhibited TLR4/MyD88/NF‐κB signaling, with TLR4, MyD88 and p‐p65 ↓; accompanied by oxidative stress attenuation	LVEF and LVFS ↑, infarct size ↓, CK‐MB, LDH, MDA, and MPO ↓; SOD ↑, apoptosis and myocardial injury ↓	[294]
Mogroside IIIE	**In vivo**: ISO‐induced myocardial fibrosis in male C57BL/6 mice **In vitro**: Ang II‐stimulated neonatal mouse cardiac fibroblasts	**In vivo**: 1 and 10 mg/kg/day, i.g., for 14 days **In vitro**: 100 µM for 48 h after Ang II stimulation	TLR–NF‐κB/inflammatory priming	Inhibited TLR4/MyD88/NF‐κB signaling, with TLR4, MyD88 and p‐NF‐κB ↓; p‐IκBα ↑, fibrosis‐related TGF‐β1, α‐SMA, and Col I/III ↓	LVIDd ↓, E/A, LVEF and LVFS ↑; inflammatory infiltration and fibrosis ↓; IL‐1β, IL‐6, TNF‐α and fibroblast proliferation ↓	[295]
Diannexin	**In vivo**: MI/RI induced by LAD occlusion for 30 min followed by reperfusion in male C57BL/6 mice **In vitro**: H/R‐treated HL‐1 cardiomyocytes and RAW264.7 macrophages	**In vivo**: 150 and 300 mg/kg, i.v., before reperfusion; assessed 5 days after MI/RI **In vitro**: 30 nM/L under H/R conditions	TLR–NF‐κB/inflammatory priming	Inhibited TLR4/NF‐κB‐mediated NLRP3 inflammasome activation, with TLR4, p‐NF‐κB, NLRP3, and ASC ↓, and reduced M1 polarization while promoting M2 polarization	LVEF and LVFS ↑, LVEDD and LVESD ↓, apoptosis, ROS, LDH, MDA, SOD, and fibrosis ↓; collagen deposition ↓, TNF‐α, IL‐6, IL‐1β, and IL‐18 ↓, IL‐10 ↑	[296]
Tilianin	**In vivo**: MI/RI induced by LAD ligation in male SD rats **In vitro**: OGD treated H9c2 cells	**In vivo**: 3, 10, and 30 mg/kg, i.p., at reperfusion onset **In vitro**: 10, 30, and 50 µg/mL during OGD	NLRP3/amplification and pyroptotic execution	Inhibited TLR4/NF‐κB‐mediated NLRP3 inflammasome activation and disrupted NEK7/NLRP3 interaction, with TLR4, p‐NF‐κB, NLRP3, ASC, NEK7, cleaved caspase‐1, cleaved GSDMD, IL‐1β, and IL‐18 ↓	H9c2 viability ↑, LVEF and LVFS ↑; LVESD, AST, cTnT, LDH, IFN/AAR, apoptosis and inflammatory infiltration ↓	[297]
Emodin	**In vivo**: HFD‐treated ApoE^−/−^ mice with atherosclerosis **In vitro**: PMA‐induced THP‐1 macrophages	**In vivo**: 10 and 20 mg/kg/day, i.g., for 6 weeks during HFD feeding **In vitro**: 5 and 10 µM pretreatment for 2 h	NLRP3/amplification and pyroptotic execution	Inhibited TLR4/MyD88/NF‐κB signaling and suppressed NLRP3/GSDMD‐associated inflammatory activation, with NLRP3, caspase‐1, GSDMD, IL‐1β, and IL‐18 ↓	Plaque area, fibrotic area, IL‐1β, IL‐18, inflammatory cytokines, macrophage inflammation and lipid accumulation ↓	[298]
Methyl‐β‐cyclodextrin	**In vivo**: HFD‐fed ApoE^−/−^ mice with atherosclerosis **In vitro**: ox‐LDL‐treated rat VSMCs	**In vivo**: 2.0 g/kg, s.c., twice weekly for 12 weeks during HFD **In vitro**: 5 mM pretreatment for 2 h	NLRP3/amplification and pyroptotic execution	Inhibited TLR4/NF‐κB/NLRP3 signaling and reduced GSDMD‐mediated pyroptosis, with TLR4, p‐NF‐κB, NLRP3, and GSDMD ↓	Plaque area ↓, CD68+ cell infiltration ↓, TG, TC and LDL‐C ↓, HDL‐c ↑, blood glucose ↓, IL‐1β ↓, IL‐18 ↓, PI‐positive cells ↓	[299]
Rutin	**In vivo**: MI/RI injury induced by LAD ligation for 30 min followed by 120 min reperfusion in C57BL/6J mice **In vitro**: H/R‐treated primary mouse cardiomyocytes	**In vivo**: 10 mg/kg, i.p., 1 h before ischemia **In vitro**: 20 µM under H/R conditions	NLRP3/amplification and pyroptotic execution	Inhibited NF‐κB/NLRP3/caspase‐1/GSDMD‐mediated pyroptosis, with ROS and inflammatory cytokines ↓	Infarct area and apoptosis ↓, CK‐MB, LDH, cTnT/I, IL‐1β, TNF‐α, and IL‐18 ↓, cell viability ↑, myocardial injury ↓	[300]
Aloe‐emodin derivative	**In vivo**: HFD and STZ‐induced diabetic cardiomyopathy in SD rats **In vitro**: High glucose‐treated H9c2 cells	**In vivo**: 50 and 100 mg/kg/day, i.g., for 4 weeks **In vitro**: 10 and 20 µM for 24 h after high glucose exposure	NLRP3/amplification and pyroptotic execution	Inhibited NLRP3 inflammasome‐mediated pyroptosis, with NLRP3, GSDMD, caspase‐1, IL‐1β, and IL‐18 ↓	Fasting glucose ↓, LVEF and LVFS ↑; fibrosis, cardiac hypertrophy and mitochondrial damage ↓, cell viability ↑, pyroptosis ↓	[301]
Nicotinamide riboside	**In vivo**: TAC‐induced cardiac hypertrophy in C57BL/6J mice	**In vivo**: 400 mg/kg/day, i.g., for 8 weeks	Immune‐metabolic gating/threshold control	Restored NAD^+^–Sirtuin3–MnSOD signaling and inhibited NLRP3 inflammasome activation, with NAD^+^ ↑, Sirtuin3 activity ↑, ac‐MnSOD ↓, NLRP3 ↓, cleaved GSDMD ↓	LVEF and LVFS ↑; HW/BW, LW/BW, ANP, BNP and LDH ↓; LVEDD and LVESD ↓, LV wall thickness ↓, IL‐1β, TNF‐α, DHE fluorescence, and MDA ↓; SOD ↑	[302]
Berberine	**In vivo**: db/db C57BL/6J mice with diabetic cardiomyopathy **In vitro**: High glucose‐treated H9c2 cells	**In vivo**: 136.5 mg/kg/day for 8 weeks **In vitro**: 20 µM under 33 mM glucose for 24 h	Immune‐metabolic gating/threshold control	Inhibited mTOR/mtROS‐driven NLRP3 inflammasome activation, with p‐mTOR, mtROS, NLRP3, caspase‐1, and GSDMD ↓	Fasting glucose ↓; LVEF and LVFS ↑; LVIDd and LVIDs ↓; collagen deposition ↓, IL‐1β, IL‐18, pyroptosis, and LDH release ↓	[303]
Empagliflozin	**In vivo**: HFD‐fed ApoE^−/−^ mice with atherosclerosis **In vitro**: ox‐LDL treated RAW264.7 macrophages	**In vivo**: 1.3 and 3.2 mg/kg/day, i.g., for 8 weeks **In vitro**: 1 and 5 µM with ox‐LDL for 24 h	Immune‐metabolic gating/inflammatory priming	Activated AMPK signaling and suppressed NF‐κB‐associated macrophage inflammation, with p‐AMPK ↑, NF‐κB, IL‐1β, IL‐6, TNF‐α, and MCP‐1 ↓	Plaque area, macrophage infiltration, serum IL‐1β, serum IL‐6, body weight, ox‐LDL uptake, and foam cell formation ↓	[304]
2‐APQC	**In vivo**: ISO‐induced heart failure in male SD rats **In vitro**: ISO‐treated H9c2 cells	**In vivo**: 10, 20, and 30 mg/kg/day, i.g., for 4 weeks **In vitro**: 10, 20, or 30 µM for 24 h	Immune‐metabolic gating/threshold control	Activated SIRT3‐dependent mitochondrial homeostasis, with PYCR1 and p‐AMPK/Parkin ↑; p‐AKT/mTOR, p‐Smad3, ROS, and Ca^2^^+^ overload ↓	LVEF and LVFS ↑; LVESD ↓, BNP, HWI, hypertrophy, fibrosis, LDH, AST, CK‐MB, α‐HBDH, and necrosis ↓	[305]

*Note*: ↑, elevation/upregulation/activation; ↓, reduction/downregulation/inhibition.

Abbreviations: Ac‐FOXO1, acetylated forkhead box O1; ADP, adenosine diphosphate; ALD, aldosterone; ALOX15, arachidonate 15‐lipoxygenase; Ang II, angiotensin II; Bax, BCL2‐associated X protein; Bcl‐2, B‐cell lymphoma 2; Col I/III, collagen I/III; cTnT, cardiac troponin T; DHE, dihydroethidium; ET, endothelin; FBG, fasting blood glucose; FFAs, free fatty acids; GLXB, Gualou–Xiebai; GPX4, glutathione peroxidase 4; GSH, glutathione; HDD, Huangqi–Danshen decoction; HFpEF, heart failure with preserved ejection fraction; HW/BW, heart weight/body weight; HW/TL, heart weight/tibia length; HWI, heart weight index; ICR, Institute of Cancer Research; INF/AAR, infarct area/area at risk; IVRT, isovolumic relaxation time; LOX‐1, lectin‐like oxidized low‐density lipoprotein receptor‐1; LVEDD, left ventricular end‐diastolic diameter; LVESD, left ventricular end‐systolic diameter; LVFS, left ventricular fractional shortening; LVIDd, left ventricular internal diameter in diastole; LVIDs, left ventricular internal diameter in systole; LW/BW, lung weight/body weight; MDA, malondialdehyde; MITOL, mitochondrial ubiquitin ligase; MMP‐9, matrix metalloproteinase‐9; MnSOD, manganese superoxide dismutase; MYSD, Modified Yiwei Shengyang decoction; NCOA4, nuclear receptor coactivator 4; NEK7, NIMA‐related kinase 7; PI, propidium iodide; p‐IκBα, phosphorylated inhibitor of κBα; PMA, phorbol 12‐myristate 13‐acetate; p‐Smad3, phosphorylated Smad3; PYCR1, pyrroline‐5‐carboxylate reductase 1; QDWX, Qingdu Wenxin Fang; QLQX, QiliQiangxin; SFYX, Shenfuyixin Granules; SLC7A11, solute carrier family 7 member 11; SOD, superoxide dismutase; TFR1, transferrin receptor 1; TGF‐β1, transforming growth factor‐β1; THP‐1, human monocytic leukemia cell line THP‐1; TIMP‐1, tissue inhibitor of metalloproteinases‐1; TMRE, tetramethylrhodamine ethyl ester; TNNT2, troponin T2, cardiac type; TYTZD, Tongyang Tongzhi decoction; XKG, Xinkang Granules; YMG, Yi Mai Granule; α‐HBDH, alpha‐hydroxybutyrate dehydrogenase; α‐SMA, alpha‐smooth muscle actin.

However, the overall preclinical evidence base remains heterogeneous. Many studies rely on small animal models, short observation windows, preventive administration, and surrogate molecular or histological endpoints rather than long‐term functional outcomes [276, 302]. Commonly used endpoints include LVEF, LVFS, infarct size, inflammatory cytokine levels, Western blot markers, ROS levels, fibrosis staining, and mitochondrial morphology. These endpoints are useful for mechanistic interpretation, but they do not fully capture durable clinical benefit. Long‐term survival, sustained ventricular remodeling, recurrent hospitalization risk, and persistent functional improvement are rarely evaluated. In this regard, the QLQX study is relatively informative because it reported improved survival in addition to functional and molecular outcomes, whereas most other studies remain focused on short‐term mechanistic endpoints.

Study design quality also varies considerably. Some studies provide relatively clear sample sizes, such as *n* = 20 per group in the QLQX study and *n* = 6 per group in many TAC‐based mechanistic studies, but randomization, blinding, sample size calculation, and allocation concealment are often incompletely reported [276, 302]. In addition, many interventions are administered before or at the time of model induction, which is useful for identifying biological mechanisms but does not fully reflect clinically relevant treatment after disease onset [279]. Head‐to‐head comparisons, multilaboratory replication, pharmacokinetic evaluation, dose–response relationships, and long‐term safety assessments are also limited [306, 307]. These limitations do not invalidate the mechanistic value of the findings, but they constrain the degree to which current results can be directly extrapolated to clinical settings.

Not all preclinical interventions have the same level of mechanistic or translational plausibility. Some strategies show relatively stronger support because they are based on well‐defined molecules, coherent pathway linkage, functional readouts, or clinically relevant pharmacological backgrounds. Among MQC‐related strategies, Ginsenoside Rb1 has been reported to improve mitophagy, suppress inflammation, and reduce apoptosis through the AMPKα‐FUNDC1/PINK1/Parkin axis in MI models [277]. Nicorandil links PINK1/Parkin‐associated MQC with reduced NLRP3 activation and improved microvascular function in chronic DCM models [283]. Canagliflozin is particularly notable because it is already used clinically and appears to improve cardiac remodeling by restraining excessive AMPK/PINK1/Parkin‐mediated mitophagy rather than simply enhancing mitophagy indiscriminately [288].

For nucleic acid‐sensing interventions, Scutellarin directly targets cGAS–STING‐associated apoptotic signaling in MI/RI models, although its evidence is still based mainly on pretreatment designs [279]. Tetrandrine connects STING/TBK1/NF‐κB signaling with inflammatory output in AS models that combine ApoE^−/−^ mice and ox‐LDL‐based cellular experiments [286]. Baicalin and Irisin further link cGAS–STING signaling with NLRP3, gasdermin D (GSDMD), or metabolic regulation, suggesting that nucleic acid sensing should be evaluated within a broader inflammatory network rather than as an isolated pathway [285, 287]. At the level of immunometabolic gating, Nicotinamide riboside, Empagliflozin, and 2‐APQC provide examples of interventions with relatively coherent network logic. Nicotinamide riboside acts through the NAD^+^–Sirtuin3–MnSOD–NLRP3 axis [302], Empagliflozin activates AMPK and suppresses NF‐κB‐associated macrophage inflammation in AS models [304], and 2‐APQC integrates SIRT3, AMPK/Parkin, and AKT/mTOR regulation within a unified framework [305].

Nevertheless, relatively stronger mechanistic plausibility should not be interpreted as clinical readiness. Rather, it means that these interventions currently have clearer molecular definitions, more coherent pathway connections, or more informative functional readouts than studies based mainly on high‐dose administration, limited endpoints, or preventive designs. Even for interventions with clearer mechanistic support, the evidence remains largely preclinical and requires further validation through therapeutic dosing designs, independent replication, pharmacokinetic characterization, and long‐term safety assessment [306, 307].

A major translational concern is dose feasibility. Many positive findings are based on relatively high or even extremely high dosing in rodent models, while human‐equivalent doses, exposure to active constituents, therapeutic index, and safety windows are not sufficiently defined [308, 309, 310]. This issue is especially relevant for natural products and multicomponent formulations. For example, TYTZD has been administered at 3.6–14.4 g/kg/day, and YMG at 4.45–17.8 g/kg/day for 21 weeks [278, 280]. Such gram‐per‐kilogram dosing does not mean that the findings lack mechanistic value, but without active constituent quantification, extract standardization, human‐equivalent dose conversion, exposure analysis, and safety evaluation, these results remain difficult to translate into clinically realistic pharmacological evidence.

Replication and comparability are additional limitations. Even interventions with clearer molecular definitions, such as Ginsenoside Rb1, Scutellarin, Tetrandrine, or Nicotinamide riboside, are often supported by single studies or a limited number of reports [277, 279, 286, 302]. Multilaboratory replication, head‐to‐head comparison, and systematic evaluation of therapeutic windows remain uncommon. Moreover, almost all studies summarized in this field report positive outcomes, such as reduced inflammation, improved cardiac function, or attenuated tissue injury, whereas neutral or negative findings are rarely available. This raises the possibility of publication bias and limits the ability to estimate true effect robustness [306, 307].

Model limitations also need to be considered. The cardiovascular inflammatory network is strongly stage dependent, cell dependent, and threshold dependent. Therefore, a target that appears beneficial in one disease stage or cell type may be ineffective or even harmful in another. For example, mitophagy enhancement may be protective when damaged mitochondria are the dominant source of danger signals, but excessive mitophagy may aggravate energetic vulnerability when mitochondrial reserve is already compromised [92, 267, 268]. Similarly, STING inhibition may be beneficial when persistent DNA sensing drives chronic inflammation, but broad suppression of nucleic acid sensing could interfere with damage recognition and immune surveillance [270]. Thus, many preclinical studies demonstrate that a pathogenic layer is modifiable, but they do not yet fully answer when, in which patients, and at what intensity intervention should be applied.

Future preclinical studies should therefore strengthen the translational interpretability of network‐guided inflammatory intervention. First, experimental design should include clearer reporting of sample size, randomization, blinding, allocation concealment, and statistical methods [276, 302]. Second, endpoints should extend beyond short‐term biochemical and histological readouts to include long‐term cardiac function, ventricular remodeling, vascular outcomes, survival, and safety. Third, natural products and multicomponent formulations should undergo stronger standardization, active constituent characterization, dose conversion analysis, pharmacokinetic evaluation, and therapeutic index assessment [278, 308, 309, 310]. Fourth, candidate interventions should be tested across different disease stages, cellular contexts, and levels of danger signal burden rather than in a single model at a single time point [263]. Only through these improvements can preclinical evidence evolve from a rich catalog of mechanistic observations into a more reliable foundation for clinical translation.

Overall, current preclinical evidence supports the feasibility of modulating cardiovascular inflammatory networks, but it should be interpreted as mechanistic and proof‐of‐concept evidence rather than mature clinical evidence. Its main value lies in identifying modifiable network nodes, clarifying pathway interactions, and guiding the design of more precise stage‐, cell‐, and context‐informed interventions. At the present stage, the most balanced conclusion is that these interventions provide a rational experimental basis for future translational development, but their clinical applicability will require stronger validation through standardized dosing, robust study design, long‐term outcomes, and careful safety evaluation.

### 7.6 Clinical Evidence and Ongoing Trials

Clinical evidence has established that inflammation is not merely an accompanying phenotype in CVDs, but a modifiable pathogenic layer that can influence cardiovascular outcomes. As summarized in Table 4, current clinical trials have mainly focused on relatively downstream inflammatory‐output layers, including the IL‐1/IL‐6 axis, broad anti‐inflammatory strategies, inflammasome‐related intervention, leukocyte recruitment, and plaque inflammation imaging. By contrast, clinical evidence targeting upstream mitochondrial danger signal input, MQC, cGAS–STING‐related nucleic acid sensing, and threshold‐gating mechanisms remains comparatively limited. Therefore, the current clinical landscape supports the principle that inflammatory pathways can be therapeutically modified in CVDs, but it has not yet fully achieved precise regulation of the upstream organizational logic of the inflammatory network.

**TABLE 4:** Representative clinical trials of inflammatory network‐targeted interventions in cardiovascular disease.

Drug	Target	Phase	Status	Patient cohort	Primary endpoint	Main findings	NCT number
Canakinumab	IL‐1β/IL‐6 axis	III	Completed	Prior MI with hsCRP ≥ 2 mg/L	Three‐point MACE	Reduced recurrent cardiovascular events without lowering LDL‐C	NCT01327846
Colchicine	Broad anti‐inflammatory	III	Completed	Recent MI	Cardiovascular death, resuscitated cardiac arrest, MI, stroke, or urgent hospitalization for angina requiring revascularization	Reduced ischemic cardiovascular events after recent MI	NCT02551094
Colchicine	Broad anti‐inflammatory	III	Completed	MI within 72 h after PCI	Cardiovascular death, recurrent MI, stroke, or unplanned ischemia‐driven revascularization	Neutral: colchicine started early after MI did not reduce the primary composite endpoint	NCT03048825
Methotrexate	Broad anti‐inflammatory	III	Completed	Stable CAD with prior MI and T2DM or metabolic syndrome	Nonfatal MI, nonfatal stroke, or cardiovascular death	Neutral: no reduction in IL‐1β, IL‐6, CRP, or recurrent cardiovascular events	NCT01594333
Anakinra	IL‐1R blockade	III	Completed	STEMI	Change in LV end‐systolic volume index safety/feasibility	Pilot study: safe and favorably affected LV remodeling after AMI	NCT00789724
Anakinra	IL‐1R blockade	II	Completed	STEMI	CRP AUC from admission to Day 14	Reduced CRP AUC exploratory/prespecified analyses suggested possible reduction in HF events/HF hospitalizations	NCT01950299
Dapansutrile	NLRP3	Ib	Completed	Stable HFrEF, NYHA II–III	Safety, tolerability, and pharmacodynamics over 14 days	Safe and well tolerated; exploratory improvement in LVEF and exercise time at the highest dose cohort	NCT03534297
Tocilizumab	IL‐6R/IL‐6 axis	II	Completed	NSTEMI	hsCRP AUC during hospitalization	Attenuated inflammatory response and reduced PCI‐related hsTnT release	NCT01491074
Tocilizumab	IL‐6R/IL‐6 axis	II	Completed	Acute STEMI	Myocardial salvage index	Increased myocardial salvage in acute STEMI	NCT03004703
Rilonacept	IL‐1 trap	III	Completed	Recurrent pericarditis	Pericarditis recurrence	Rapid symptom resolution and markedly lower recurrence risk than placebo	NCT03737110
Inclacumab	P‐selectin/leukocyte recruitment	II	Completed	NSTEMI undergoing PCI	Periprocedural myocardial damage after PCI	Reduced myocardial damage after PCI, with greater benefit when infused < 3 h before PCI	NCT01327183
Losmapimod	MAPK	III	Completed	Acute MI/ACS	Major ischemic cardiovascular events	Negative outcome: did not reduce major ischemic cardiovascular events	NCT02145468
Ziltivekimab	IL‐6 ligand/IL‐6 axis	II	Completed	CKD Stage 3–5 with systemic inflammation and high cardiovascular risk	Reduction in hsCRP/inflammatory biomarkers	Dose‐dependent hsCRP reduction (77%–92%) and lower thrombosis‐related biomarkers; provided the biologic rationale for Phase 3 outcome trials	NCT03926117
Ziltivekimab	IL‐6 ligand/IL‐6 axis	III	Active, not recruiting	Patients with ASCVD, CKD, and systemic inflammation	Time to first three‐point MACE	Ongoing Phase 3 outcome trial; no interim efficacy results publicly posted	NCT05021835
Ziltivekimab	IL‐6 ligand/IL‐6 axis	III	Recruiting	HFmrEF/HFpEF with inflammation	Time to first cardiovascular death, HF hospitalization, or urgent HF visit	Recruiting Phase 3 trial; no interim efficacy readout identified in this search	NCT05636176
Ziltivekimab	IL‐6 ligand/IL‐6 axis	III	Recruiting	Recent STEMI/NSTEMI with high‐risk features	Time to first three‐point MACE	Recruiting Phase 3 trial; no interim efficacy readout identified in this search	NCT06118281
Anakinra	IL‐1R blockade	II	Recruiting	Acute STEMI after successful reperfusion	Peak VO_2_ at 42 ± 7 days	Recruiting mechanistic/early translational trial; no interim efficacy results publicly posted	NCT05177822
DFV890	NLRP3‐related signaling	IIa	Completed	Coronary heart disease with elevated hsCRP	Change in serum IL‐6 and IL‐18 through Day 92	Recently completed early‐phase biomarker trial; no results posted	NCT06031844
Orticumab	ox‐LDL‐related plaque inflammation	IIb	Recruiting	Prior MI with elevated coronary inflammation by FAI score	Change in mean FAI score at Week 24	Recruiting imaging/mechanistic trial; no interim efficacy readout identified in this search	NCT06927739

*Source*: The clinical trial information summarized in Table 4 was obtained from ClinicalTrials.gov (https://clinicaltrials.gov).

Abbreviations: ASCVD, atherosclerotic cardiovascular disease; AUC, area under the curve; CAD, coronary artery disease; CHD, coronary heart disease; CKD, chronic kidney disease; CRP, C‐reactive protein; FAI, fat attenuation index; HFmrEF, heart failure with mildly reduced ejection fraction; HFpEF, heart failure with preserved ejection fraction; HFrEF, heart failure with reduced ejection fraction; hsCRP, high‐sensitivity C‐reactive protein; hsTnT, high‐sensitivity cardiac troponin T; LV, left ventricular; LVEF, left ventricular ejection fraction; MACE, major adverse cardiovascular events; NSTEMI, non‐ST‐segment elevation myocardial infarction; NYHA, New York Heart Association; PCI, percutaneous coronary intervention; STEMI, ST‐segment elevation myocardial infarction; T2DM, Type 2 diabetes mellitus; VO2, oxygen uptake.

The most convincing evidence currently comes from interventions targeting downstream cytokine amplification and inflammatory output. Canakinumab reduced recurrent cardiovascular events in patients with prior MI and elevated high‐sensitivity C‐reactive protein (hsCRP), independent of low‐density lipoprotein cholesterol (LDL‐C) lowering, thereby providing direct clinical support for the concept of residual inflammatory risk [311]. Consistent with this principle, anakinra reduced C‐reactive protein (CRP) burden in ST‐segment elevation myocardial infarction (STEMI) studies and exploratory analyses suggested a possible reduction in HF‐related events [312]. Tocilizumab also attenuated inflammatory responses, reduced percutaneous coronary intervention (PCI)‐related myocardial injury, and increased myocardial salvage in non‐ST‐segment elevation myocardial infarction (NSTEMI) and acute STEMI settings [313]. In parallel, ziltivekimab markedly reduced hsCRP in high‐risk patients with chronic kidney disease (CKD) and systemic inflammation and has advanced into Phase III outcome trials [314]. These studies indicate that the first clinically validated targets in cardiovascular inflammation are located mainly at the downstream cytokine‐output layer rather than at the most upstream level of danger signal generation.

At the same time, current clinical evidence also shows that broader anti‐inflammatory suppression does not necessarily translate into greater cardiovascular benefit. Methotrexate failed to reduce IL‐1β, IL‐6, CRP, or recurrent cardiovascular events in patients with stable coronary artery disease, prior MI, and metabolic abnormalities [315]. Colchicine reduced ischemic cardiovascular events in patients with recent MI in one large trial, whereas another trial initiated very early after MI did not show improvement in the primary composite endpoint [316, 317]. Losmapimod likewise failed to reduce major ischemic events in acute MI or acute coronary syndrome (ACS) [318]. These findings suggest that cardiovascular inflammation is not a homogeneous process that can be consistently improved by nonspecific anti‐inflammatory intervention or by treatment delivered within a single fixed time window. Instead, therapeutic efficacy appears to depend strongly on disease stage, inflammatory layer, patient context, and timing of intervention.

Compared with cytokine‐output and broad anti‐inflammatory strategies, interventions targeting intermediate or upstream layers of the inflammatory network remain at an earlier stage of clinical validation. Dapansutrile, an NLRP3‐related intervention, has mainly demonstrated short‐term safety and tolerability in stable heart failure with reduced ejection fraction (HFrEF), with exploratory functional improvement in LVEF and exercise time observed only in the highest dose cohort [319]. Other approaches, including NLRP3‐related biomarker studies, P‐selectin/leukocyte recruitment targeting with inclacumab, and ox‐LDL‐related plaque inflammation imaging with orticumab, indicate that clinical trials are gradually extending beyond terminal cytokine blockade toward inflammasome activity, leukocyte recruitment, plaque inflammation, and other intermediate network layers [320]. However, these approaches still largely remain in the stage of mechanistic or biomarker validation rather than definitive outcome confirmation.

This clinical landscape has important implications for the network‐guided perspective discussed in this review. The closer an intervention is to downstream inflammatory output, the more extensive the current clinical validation; the closer it is to upstream danger signal input, MQC, nucleic acid sensing, and threshold gating, the more preliminary the evidence remains. This imbalance does not mean that upstream or network‐gating strategies lack therapeutic potential. Rather, it reflects the greater complexity of translating these targets into clinical trials, because their effects are likely to depend on disease stage, cell type, mitochondrial damage burden, metabolic status, and safety constraints. For example, broad inhibition of IL‐1 or IL‐6 can be evaluated through established inflammatory biomarkers and cardiovascular endpoints, whereas modulation of MQC, cGAS–STING, NLRP3, or immunometabolic gating requires more precise patient selection and mechanism‐informed endpoints.

Safety considerations also become increasingly important as clinical trials move toward upstream and network‐regulatory targets. Existing cytokine‐targeted therapies have shown that anti‐inflammatory benefit may coexist with infection or immune‐suppression risks [311]. For future interventions targeting NLRP3, cGAS–STING, broader nucleic acid‐sensing pathways, mitophagy, or MQC, safety assessment will need to extend beyond general adverse events and incorporate mechanism‐informed monitoring.

Overall, current clinical evidence supports the principle that inflammatory pathways can be therapeutically modified in CVDs, but it also highlights the limits of nonspecific or poorly stratified anti‐inflammatory strategies. The strongest outcome data currently support downstream inflammatory‐output modulation, whereas interventions targeting inflammasomes, nucleic acid sensing, MQC, and threshold‐gating mechanisms still require more rigorous clinical validation. Future translational progress will depend on aligning therapeutic targets with disease stage, dominant inflammatory layer, patient phenotype, biomarker profile, and safety requirements. In this sense, clinical trial development should increasingly shift from broad inflammatory suppression toward precision regulation of cardiovascular inflammatory networks.

### 7.7 Unanswered Questions and Future Mechanistic Priorities

Although preclinical studies and selected clinical trials have shown that the cardiovascular inflammatory network is therapeutically tractable, several key questions remain unresolved. The central challenge is no longer simply to identify additional inflammatory molecules, but to determine how a dynamic network characterized by stage dependence, cell specificity, and threshold‐dependent behavior can be translated into an operational clinical framework. In this context, the most important questions are not limited to which inflammatory molecule should be inhibited. They also include when intervention should be applied, which network layer should be prioritized, which patient population is most likely to benefit, and how target engagement and biological response can be reliably evaluated in vivo.

First, the temporal dependence of inflammatory signaling remains insufficiently defined. The same module, such as mitophagy, NLRP3, or cGAS–STING, may have distinct or even opposing roles across acute injury, chronic inflammatory maintenance, and maladaptive remodeling stages. In the acute phase, moderate inflammatory activation may contribute to damage recognition, removal of necrotic tissue, and initiation of repair. In chronic stages, the same signals may instead sustain inflammatory amplification, fibrosis, and remodeling maintenance. Without precise identification of disease stage, premature or excessive inhibition may suppress necessary adaptive responses, whereas delayed intervention may fail to reverse an established pathological network [222, 271, 321]. Therefore, future studies should define stage‐specific therapeutic windows rather than treating inflammatory pathways as fixed pathogenic targets.

Second, cell type‐specific mechanisms remain a major barrier to translation. Cardiomyocytes, endothelial cells, macrophages, fibroblasts, neutrophils, and circulating immune cells occupy different positions within the inflammatory network. Cardiomyocytes may act as early sources of mitochondrial DAMPs during ischemic or toxic injury; endothelial cells regulate barrier function, adhesion molecule expression, and leukocyte recruitment; macrophages amplify inflammation through cytokine release, inflammasome activation, and metabolic reprogramming; and fibroblasts convert persistent inflammatory signaling into extracellular matrix deposition and fibrosis. Because most current interventions are administered systemically, the same target may produce different effects across cellular compartments. Future mechanistic studies should therefore clarify which cell type drives the pathogenic network in each disease stage and should develop strategies capable of improving tissue or cell selectivity [7, 247].

Third, clinically applicable biomarkers remain underdeveloped. A major gap between mechanistic studies and clinical trials is the lack of a biomarker system that can dynamically reflect inflammatory threshold states. For clinical execution, early‐phase studies should prioritize markers already integrated into cardiovascular trials, including hsCRP, IL‐6, N‐terminal pro‐B‐type natriuretic peptide (NT‐proBNP), high‐sensitivity cardiac troponin (hs‐cTn), echocardiographic indices such as LVEF, global longitudinal strain (GLS), and diastolic function, and cardiac magnetic resonance (CMR)‐based readouts of infarct size, ventricular remodeling, and fibrosis burden [214, 322, 323]. These markers are clinically practical but remain relatively downstream. For mechanistic exploration, more proximal biomarkers may be needed, including circulating cell‐free mtDNA, plasma cGAMP, and phosphorylation states of STING pathway components in peripheral blood mononuclear cells (PBMCs), such as p‐STING, p‐TBK1, and p‐IRF3 [67, 324, 325]. Future trials should combine clinically executable markers with mechanism‐oriented markers rather than relying on a single inflammatory indicator. While mechanistically attractive, these proximal markers are currently more suitable for centralized pharmacodynamic assessment or subgroup analysis than as universal inclusion criteria, because their use in patient selection or treatment allocation requires sufficiently validated analytical performance. Advanced approaches such as mitochondrial functional imaging, single‐cell immunomics, and complex multiomics readouts are therefore more appropriate as exploratory substudies at present, rather than core execution metrics in most early‐phase trials [326, 327].

Fourth, endpoint design should avoid oversimplification and should better match the network layer being targeted. Reliance on the reduction of a single inflammatory mediator as the primary efficacy measure is insufficient to capture the full impact of network‐guided intervention. A more appropriate approach in early‐phase trials is to adopt composite endpoints integrating mechanistic, imaging, physiological, and clinical domains. Recent studies in cardiometabolic populations suggest that elevated cell‐free mtDNA is associated with stronger systemic inflammation, greater congestion, and poorer exercise response, indicating its potential as a circulating readout of mitochondrial stress [328, 329]. Meanwhile, CMR remains uniquely valuable in postinfarction remodeling studies because it provides integrated information on cardiac structure, function, tissue characteristics, and fibrosis progression, making it well suited as both a mechanistic and prognostic endpoint [323, 330, 331]. In patients with acute ischemic injury, changes in hsCRP, IL‐6, cell‐free mtDNA, or IL‐18 may be combined with CMR‐derived measures of left ventricular remodeling, infarct size, or myocardial salvage, together with NT‐proBNP, symptom burden, and early HF events [323, 332, 333]. In diabetic cardiomyopathy or metabolic myocardial injury, a more appropriate strategy is to integrate low‐grade inflammatory markers, imaging‐based fibrosis readouts, native T1 mapping in selected contexts, and quality‐of‐life assessments to more comprehensively reflect early pathological phenotypes and their trajectories [334, 335].

These considerations further highlight that defining efficacy solely by changes in a single inflammatory factor provides limited information. In proof‐of‐mechanism trials, a more informative approach is to simultaneously assess three categories of signals [336]. The first includes mechanistic signals, indicating that inflammatory or danger‐sensing activity has indeed been modulated. The second includes imaging or physiological signals, reflecting changes in myocardial remodeling trajectory or functional status. The third includes clinical signals such as natriuretic peptide levels, symptom burden, or early worsening events. Such composite endpoints better align with the nature of network‐guided intervention, since strategies targeting MQC, NLRP3, or cGAS–STING often first influence danger signal input, inflammatory threshold status, and the rate of remodeling progression, rather than immediately translating into differences in traditional hard endpoints [323, 329].

Fifth, patient selection should move beyond broad disease labels. In postmyocardial infarction studies, the most appropriate population may not be all survivors, but those with residual inflammatory activity and high remodeling risk, such as patients with persistently elevated hsCRP or IL‐6, increased NT‐proBNP, and CMR evidence of large infarct burden, microvascular obstruction, or early adverse remodeling trends [323, 337]. In diabetic cardiomyopathy or metabolic myocardial inflammation, enrichment strategies should focus on patients with coexisting metabolic stress, low‐grade persistent inflammation, and evidence of mitochondrial danger signal release [334]. This may include patients with Type 2 diabetes, obesity, insulin resistance, CKD, HFpEF or HFmrEF tendency, subclinical myocardial dysfunction, persistent low‐grade inflammation, and, when feasible, abnormal circulating mtDNA. In other words, future studies should enroll patients in whom the targeted inflammatory network is biologically active, rather than relying only on diagnostic labels.

In terms of safety monitoring, future network‐guided trials should establish layer‐matched alert systems. For interventions targeting IL‐1/IL‐6, NLRP3, or broader inflammatory output layers, routine monitoring should include infection events, leukopenia, neutropenia, liver function abnormalities, and immunosuppression‐related adverse effects. Clinical experience with canakinumab has already shown that anti‐inflammatory benefit may coexist with increased risk of fatal infection or sepsis [311]. For long‐term inhibition of cGAS–STING or broader nucleic acid‐sensing pathways, safety assessment should extend beyond general adverse events to include viral infection, latent virus reactivation, reduced vaccine responses, and potential impairment of tumor immune surveillance [338, 339, 340]. When feasible, baseline evaluation may include infection history, vaccination status, latent viral status, and prior oncologic history, with follow‐up monitoring for infection, viral reactivation, new malignancies or immune‐related abnormalities [340, 341, 342]. Interventions targeting mitophagy and MQC should also not be assumed to be inherently safe, because excessive mitochondrial clearance may disturb the balance between energy supply and demand, impair myocardial adaptation, reduce exercise tolerance, or cause metabolic side effects [4, 343, 344]. Therefore, early‐phase clinical studies should not focus solely on efficacy signals, but must integrate mechanism‐informed safety monitoring throughout patient selection, sampling, endpoint design, and long‐term follow‐up.

Based on this logic, future trial designs could be organized around mechanism‐informed prototypes. One example is a post‐MI Phase IIa study performed after completion of acute reperfusion but before stabilization of ventricular remodeling. Inclusion criteria could include elevated hsCRP, increased cell‐free mtDNA, and baseline CMR evidence of substantial infarct size or increasing left ventricular end‐systolic volume index. The intervention could test strategies targeting upstream danger signal input, intermediate inflammatory amplification, or nucleic acid sensing. Primary endpoints at 8–12 weeks may include hsCRP or IL‐6, circulating mtDNA, and CMR‐derived remodeling indices, with NT‐proBNP as a parallel clinical signal. A second prototype could focus on diabetic cardiomyopathy or metabolic myocardial inflammation, enrolling patients with Type 2 diabetes, early myocardial dysfunction, and persistent low‐grade inflammation. Endpoints over 12–24 weeks could include inflammatory markers, circulating mtDNA, GLS or diastolic function, NT‐proBNP, and fibrosis‐oriented CMR readouts, with exercise capacity and quality‐of‐life measures as secondary endpoints.

Future mechanistic priorities should also include better integration of spatial, temporal, and cellular information. Single‐cell and spatial multiomics, lineage tracing, cell type‐specific genetic models, and longitudinal biomarker profiling may help clarify how inflammatory networks evolve across disease stages and how mitochondrial danger signaling differs among cardiomyocytes, endothelial cells, macrophages, and fibroblasts. Such approaches are particularly important for resolving apparently conflicting findings, such as whether mitophagy should be enhanced or inhibited, whether STING activation is adaptive or harmful, and why NLRP3 blockade may show different effects across disease models. These questions cannot be answered by pathway‐level analysis alone; they require mapping inflammatory modules to disease stage, cell type, and threshold status.

Overall, future progress in cardiovascular inflammatory therapy will depend on moving from pathway‐centered inhibition toward mechanism‐informed network regulation. The most important unresolved issues include defining stage‐specific therapeutic windows, identifying dominant cellular drivers, developing biomarkers of mitochondrial danger signaling and inflammatory threshold states, designing endpoints that match the targeted network layer, and selecting patients whose disease biology reflects active inflammatory network dysregulation. Addressing these priorities will be essential for transforming network‐guided inflammatory intervention from a mechanistic concept into a clinically actionable strategy.

## 8 Conclusion and Prospects

Cardiovascular inflammation is increasingly understood as a dynamic immune network rather than a linear consequence of single‐pathway activation. In this review, we integrated innate immune sensing, inflammasome‐related amplification, nucleic acid recognition, immunometabolic regulation, immunothrombosis, adaptive immune remodeling, and inflammatory resolution into a unified framework. Within this network, MQC and mitochondria‐derived danger signals occupy an upstream organizing position by linking metabolic stress, cellular injury, and sterile immune activation. The stage‐cell‐threshold perspective further helps explain why the same inflammatory pathway may exert adaptive, neutral, or pathogenic effects depending on disease stage, dominant cell type, signal intensity, mitochondrial reserve, and resolution capacity. Based on this integrated view, several major conclusions and future priorities emerge.

### 8.1 Inflammatory Network Mapping

Future studies should move beyond interpreting TLR signaling, NLRP3 inflammasome activation, cGAS–STING signaling, mitophagy, or immunometabolic regulation as isolated modules. These pathways are interconnected through shared danger signals, overlapping transcriptional programs, and reciprocal feedback between mitochondrial stress and immune activation. The next priority is to define how these networks are organized across time, tissue space, and cell type in human CVDs. Single‐cell sequencing, spatial transcriptomics, spatial proteomics, lineage tracing, and longitudinal biomarker profiling may help distinguish cardiomyocyte‐derived danger signaling, endothelial inflammatory entry, macrophage amplification, fibroblast remodeling, and adaptive immune persistence. Such spatiotemporal mapping will be essential for identifying the dominant pathogenic driver in each disease context and for explaining why similar inflammatory pathways can produce different outcomes across ischemic injury, atherosclerosis, diabetic cardiomyopathy, pressure overload, cardiotoxicity, and cardiomyopathy.

### 8.2 Mitochondrial DAMP Thresholds

Mitochondria should be regarded not only as metabolic organelles, but also as upstream regulators of inflammatory threshold states. When MQC is sufficient, damaged mitochondria can be repaired, isolated, or removed before mtDNA, mtROS, CL, oxidized lipids, ATP, and other DAMPs become immunologically active. When this buffering capacity fails, mitochondrial stress is converted into sustained inflammatory input. A major future priority is to determine which combinations of mtDNA release, mtROS accumulation, impaired mitophagy, mitochondrial membrane instability, NAD^+^ depletion, and redox imbalance define the transition from adaptive stress response to pathological inflammation. Establishing measurable mitochondrial DAMP thresholds may transform this concept from a mechanistic framework into a clinically useful disease‐state indicator.

### 8.3 Stage‐ and Cell‐Specific Regulation

The therapeutic meaning of mitophagy, NLRP3, cGAS–STING, and related inflammatory modules cannot be reduced to simple enhancement or inhibition. Mitophagy may be protective when damaged mitochondria are the dominant source of DAMPs, but excessive mitochondrial clearance may worsen energy deficiency when mitochondrial reserve is already compromised. Similarly, cGAS–STING inhibition may be beneficial when persistent DNA sensing drives chronic inflammation, but indiscriminate suppression may impair damage recognition, host defense, and immune surveillance. Future therapies should therefore be designed according to disease stage, dominant cell type, and threshold status. In cardiomyocytes, preserving mitochondrial reserve and limiting mtDNA leakage may be central; in endothelial cells, maintaining barrier integrity and limiting leukocyte recruitment may be more relevant; in macrophages, controlling inflammasome activation and inflammatory polarization may be critical; and in fibroblasts, preventing inflammatory‐to‐fibrotic conversion may become the main priority.

### 8.4 Targeted Drug Development and Mechanism‐Informed Translation

Future drug development should move beyond broad anti‐inflammatory agents and focus on network‐defined targets with clear disease‐stage and cell‐type relevance. Promising directions include selective regulation of MQC and mitophagy, development of safer and more context‐specific NLRP3 or cGAS–STING modulators, restoration of NAD^+^–sirtuin and AMPK/mTOR‐related immunometabolic gating, and discovery of agents that promote inflammatory resolution rather than simply suppress inflammatory output. For natural products and multicomponent formulations, future work should strengthen active constituent identification, target deconvolution, pharmacokinetic characterization, dose standardization, and safety evaluation. In parallel, advanced delivery strategies, including mitochondrial‐targeted delivery, macrophage‐ or endothelial‐directed nanocarriers, controlled‐release systems, and tissue‐responsive formulations, may improve therapeutic precision while reducing systemic immune suppression. Clinical translation will further require validated biomarkers, patient enrichment strategies, mechanism‐informed endpoints, and safety monitoring systems matched to the inflammatory network layer being targeted.

Overall, cardiovascular inflammation should be viewed as a stage‐dependent, cell‐organized, and threshold‐regulated immune network shaped by mitochondrial danger signaling and immunometabolic control. The central challenge for the field is no longer simply to identify additional inflammatory targets, but to determine how inflammatory networks are initiated, spatially organized, temporally sustained, and eventually resolved or fixed into pathological remodeling. Future progress will depend on the integration of mitochondrial DAMP biology, spatially resolved immune mapping, quantitative threshold biomarkers, targeted drug development, and mechanism‐informed clinical trial systems. Such integration may transform cardiovascular inflammatory research from pathway description toward predictive network medicine, in which disease stage, cellular context, danger signal burden, and immune‐metabolic state jointly guide therapeutic decision‐making. Ultimately, this shift from broad inflammatory suppression to precise network regulation may open a new era for preventing inflammatory injury, interrupting adverse remodeling, and improving long‐term outcomes in CVDs.

## Author Contributions

**Jiaxiang Rong**: writing – original draft, writing – review and editing, visualization. **Zhen Wang**: writing – original draft, writing – review and editing, visualization. **Xiaoxiao Lin**: methodology, visualization. **Ziwen Lei**: formal analysis. **Qianqian Huang**: reviewing, revising, conceptualization, supervision. **Hang Liu**: formal analysis, funding acquisition. **Fei Luan, Junbo Zou, and Yajun Shi**: reviewing, revising, conceptualization, supervision, funding acquisition. All the authors have read and approved the final manuscript.

## Funding

This study was financially supported by the Shaanxi Provincial Administration of Traditional Chinese Medicine Research Projects (No. SZY‐KJCYC‐2025‐JC‐043), the Basic Medicine Research Innovation Center for Cardiometabolic Diseases, Ministry of Education Open Projects Fund (No. xnykdxcxzx‐2024‐06), the Shaanxi Province Postdoctoral Research Funding Project (No. 2025BSHSDZZ390), the Science and Technology Innovative Talent Program of Shaanxi University of Chinese Medicine (No. 2024‐CXTD‐03), the Science and Technology Innovative Team Project of Shaanxi Administration of Traditional Chinese Medicine (No. 2025‐CXTD‐02), and the Key Research & Development Program of Shaanxi Provincial Department of Science and Technology (No. 2024SF‐ZDCYL‐03‐21, No. 2024CY‐JJQ‐36, No. 2024CY‐JJQ‐78).

## Ethics Statement

The authors have nothing to report.

## Conflicts of Interest

The authors declare no conflicts of interest.

## Supporting information




**Supporting Information File**: mco270878‐sup‐0001‐SuppMat.docx

## Data Availability

The authors have nothing to report.

## References

[1] S. E. Engelen , A. J. B. Robinson , Y. X. Zurke , et al., “Therapeutic Strategies Targeting Inflammation and Immunity in Atherosclerosis: How to Proceed?,” Nature Reviews Cardiology 19, no. 8 (2022): 522–542. 35102320 10.1038/s41569-021-00668-4 PMC8802279

[2] T. Donia and A. Khamis , “Management of Oxidative Stress and Inflammation in Cardiovascular Diseases: Mechanisms and Challenges,” Environmental Science and Pollution Research International 28, no. 26 (2021): 34121–34153. 33963999 10.1007/s11356-021-14109-9

[3] M. M. Redfield and B. A. Borlaug , “Heart Failure With Preserved Ejection Fraction: A Review,” JAMA 329, no. 10 (2023): 827–838. 36917048 10.1001/jama.2023.2020

[4] G. D. Lopaschuk , Q. G. Karwi , R. Tian , et al., “Cardiac Energy Metabolism in Heart Failure,” Circulation Research 128, no. 10 (2021): 1487–1513. 33983836 10.1161/CIRCRESAHA.121.318241 PMC8136750

[5] G. Heusch , “Myocardial Ischemia/Reperfusion: Translational Pathophysiology of Ischemic Heart Disease,” Med 5, no. 1 (2024): 10–31. 38218174 10.1016/j.medj.2023.12.007

[6] Y. Döring , E. P. C. Van Der Vorst , and C. Weber , “Targeting Immune Cell Recruitment in Atherosclerosis,” Nature Reviews Cardiology 21, no. 11 (2024): 824–840. 38664575 10.1038/s41569-024-01023-z

[7] X. Wang , L. Chen , J. Wei , et al., “The Immune System in Cardiovascular Diseases: From Basic Mechanisms to Therapeutic Implications,” Signal Transduction and Targeted Therapy 10, no. 1 (2025): 166. 40404619 10.1038/s41392-025-02220-z PMC12098830

[8] K. Stark and S. Massberg , “Interplay Between Inflammation and Thrombosis in Cardiovascular Pathology,” Nature Reviews Cardiology 18, no. 9 (2021): 666–682. 33958774 10.1038/s41569-021-00552-1 PMC8100938

[9] F. Porsch and C. J. Binder , “Autoimmune Diseases and Atherosclerotic Cardiovascular Disease,” Nature Reviews Cardiology 21, no. 11 (2024): 780–807. 38937626 10.1038/s41569-024-01045-7

[10] J. Mou , Y. Chen , X. Zhu , et al., “Emerging Role of the cGAS‐STING Pathway in Cardiovascular Diseases: Biologic Function, Mechanisms and Targeted Therapy,” Molecular Medicine 31, no. 1 (2025): 218. 40461989 10.1186/s10020-025-01273-8 PMC12135543

[11] Z. Zhang , H. Zhou , X. Ouyang , et al., “Multifaceted Functions of Sting in Human Health and Disease: From Molecular Mechanism to Targeted Strategy,” Signal Transduction and Targeted Therapy 7, no. 1 (2022): 394. 36550103 10.1038/s41392-022-01252-z PMC9780328

[12] S. K. Mohanta , C. Heron , A. Klaus‐Bergmann , et al., “Metabolic and Immune Crosstalk in Cardiovascular Disease,” Circulation Research 136, no. 11 (2025): 1433–1453. 40403115 10.1161/CIRCRESAHA.125.325496 PMC12286643

[13] G. Fredman and C. N. Serhan , “Specialized Pro‐Resolving Mediators in Vascular Inflammation and Atherosclerotic Cardiovascular Disease,” Nature Reviews Cardiology 21, no. 11 (2024): 808–823. 38216693 10.1038/s41569-023-00984-x PMC12863063

[14] R. S. Wang , B. A. Maron , and J. Loscalzo , “Multiomics Network Medicine Approaches to Precision Medicine and Therapeutics in Cardiovascular Diseases,” Arteriosclerosis, Thrombosis, and Vascular Biology 43, no. 4 (2023): 493–503. 36794589 10.1161/ATVBAHA.122.318731 PMC10038904

[15] P. Rai and M. B. Fessler , “Mechanisms and Effects of Activation of Innate Immunity by Mitochondrial Nucleic Acids,” International Immunology 37, no. 3 (2024): 133–142. 10.1093/intimm/dxae052 PMC13031004 39213393

[16] S. Wang , H. Long , L. Hou , et al., “The Mitophagy Pathway and Its Implications in Human Diseases,” Signal Transduction and Targeted Therapy 8, no. 1 (2023): 304. 37582956 10.1038/s41392-023-01503-7 PMC10427715

[17] A. Phillip West and P. J. Mcguire , “Tipping the Balance: Innate and Adaptive Immunity in Mitochondrial Disease,” Current Opinion in Immunology 95 (2025): 102566. 40424975 10.1016/j.coi.2025.102566 PMC12210220

[18] P. Libby , “The Changing Landscape of Atherosclerosis,” Nature 592, no. 7855 (2021): 524–533. 33883728 10.1038/s41586-021-03392-8

[19] T. J. Guzik , R. Nosalski , P. Maffia , et al., “Immune and Inflammatory Mechanisms in Hypertension,” Nature Reviews Cardiology 21, no. 6 (2024): 396–416. 38172242 10.1038/s41569-023-00964-1

[20] J. Kim , H.‐S. Kim , and J. H. Chung , “Molecular Mechanisms of Mitochondrial DNA Release and Activation of the cGAS‐STING Pathway,” Experimental & Molecular Medicine 55, no. 3 (2023): 510–519. 36964253 10.1038/s12276-023-00965-7 PMC10037406

[21] M. Ma , W. Jiang , and R. Zhou , “Damps and Damp‐Sensing Receptors in Inflammation and Diseases,” Immunity 57, no. 4 (2024): 752–771. 38599169 10.1016/j.immuni.2024.03.002

[22] E. Vringer and S. W. G. Tait , “Mitochondria and Cell Death‐Associated Inflammation,” Cell Death and Differentiation 30, no. 2 (2023): 304–312. 36447047 10.1038/s41418-022-01094-w PMC9950460

[23] J. Antonello and P. Roy , “Damage‐Associated Molecular Patterns (DAMPs) in Vascular Diseases,” Journal of Biological Chemistry 301, no. 6 (2025): 110241. 40381697 10.1016/j.jbc.2025.110241 PMC12173740

[24] Y. Zhou , P. J. Little , L. Downey , et al., “The Role of Toll‐Like Receptors in Atherothrombotic Cardiovascular Disease,” ACS Pharmacology & Translational Science 3, no. 3 (2020): 457–471. 32566912 10.1021/acsptsci.9b00100 PMC7296543

[25] T. Kawai , M. Ikegawa , D. Ori , et al., “Decoding Toll‐Like Receptors: Recent Insights and Perspectives in Innate Immunity,” Immunity 57, no. 4 (2024): 649–673. 38599164 10.1016/j.immuni.2024.03.004

[26] S. Toldo and A. Abbate , “The Role of the NLRP3 Inflammasome and Pyroptosis in Cardiovascular Diseases,” Nature Reviews Cardiology 21, no. 4 (2024): 219–237. 37923829 10.1038/s41569-023-00946-3 PMC11550901

[27] A. Larabi , J. M. Devos , S.‐L. Ng , et al., “Crystal Structure and Mechanism of Activation of Tank‐Binding Kinase 1,” Cell Reports 3, no. 3 (2013): 734–746. 23453971 10.1016/j.celrep.2013.01.034

[28] B. Zhao , C. Shu , X. Gao , et al., “Structural Basis for Concerted Recruitment and Activation of Irf‐3 by Innate Immune Adaptor Proteins,” PNAS 113, no. 24 (2016): E3403–E3412. 27302953 10.1073/pnas.1603269113 PMC4914169

[29] S. Dvorkin , S. Cambier , H. E. Volkman , et al., “New Frontiers in the cGAS‐STING Intracellular DNA‐Sensing Pathway,” Immunity 57, no. 4 (2024): 718–730. 38599167 10.1016/j.immuni.2024.02.019 PMC11013568

[30] Z. Bekassy , I. Lopatko Fagerström , M. Bader , et al., “Crosstalk Between the Renin–Angiotensin, Complement and Kallikrein–Kinin Systems in Inflammation,” Nature Reviews Immunology 22, no. 7 (2022): 411–428. 10.1038/s41577-021-00634-8 PMC8579187 34759348

[31] P. Alcaide , M. Kallikourdis , R. Emig , et al., “Myocardial Inflammation in Heart Failure With Reduced and Preserved Ejection Fraction,” Circulation Research 134, no. 12 (2024): 1752–1766. 38843295 10.1161/CIRCRESAHA.124.323659 PMC11160997

[32] Y. K. Alshoubaki , B. Nayer , Y. Z. Lu , et al., “Tregs Delivered Post‐Myocardial Infarction Adopt an Injury‐Specific Phenotype Promoting Cardiac Repair via Macrophages in Mice,” Nature Communications 15, no. 1 (2024): 6480. 10.1038/s41467-024-50806-y PMC11294480 39090108

[33] R. Pandit , H. Hillman , J. W. Williams , et al., “More Than a Cleanup Crew: The Expanding Biology of Efferocytosis,” Arteriosclerosis, Thrombosis, and Vascular Biology 46, no. 2 (2026): e323211. 41410050 10.1161/ATVBAHA.125.323211 PMC13085017

[34] M. Jin , J. Fang , J. J. Wang , et al., “Regulation of Toll‐Like Receptor (TLR) Signaling Pathways in Atherosclerosis: From Mechanisms to Targeted Therapeutics,” Acta Pharmacologica Sinica 44, no. 12 (2023): 2358–2375. 37550526 10.1038/s41401-023-01123-5 PMC10692204

[35] Y. Zhang , J. Wu , E. Dong , et al., “Toll‐Like Receptors in Cardiac Hypertrophy,” Frontiers in Cardiovascular Medicine 10 (2023): 1143583. 37113698 10.3389/fcvm.2023.1143583 PMC10126280

[36] S. R. Dubey , C. Turnbull , A. Pandey , et al., “Molecular Mechanisms and Regulation of Inflammasome Activation and Signaling: Sensing of Pathogens and Damage Molecular Patterns,” Cellular & Molecular Immunology 22, no. 11 (2025): 1313–1344. 41062723 10.1038/s41423-025-01354-y PMC12575685

[37] H. J. Shin , I. S. Kim , J. K. Kim , et al., “Molecular Mechanisms of NLRP3 Inflammasome Activation,” Experimental & Molecular Medicine 58, no. 3 (2026): 650–663. 41876821 10.1038/s12276-026-01656-9 PMC13049041

[38] S. Paik , J. K. Kim , H. J. Shin , et al., “Updated Insights Into the Molecular Networks for NLRP3 Inflammasome Activation,” Cellular & Molecular Immunology 22, no. 6 (2025): 563–596. 40307577 10.1038/s41423-025-01284-9 PMC12125403

[39] P. Broz , “Pyroptosis: Molecular Mechanisms and Roles in Disease,” Cell Research 35, no. 5 (2025): 334–344. 40181184 10.1038/s41422-025-01107-6 PMC12012027

[40] M. Khair , M. Khair , V. N. Vangaveti , et al., “The Role of the NLRP3 Inflammasome in Atherosclerotic Disease: Systematic Review and Meta‐Analysis,” Journal of Cardiology 84, no. 1 (2024): 14–21. 38521117 10.1016/j.jjcc.2024.03.003

[41] Q. Chen , L. Sun , and Z. J. Chen , “Regulation and Function of the cGAS–STING Pathway of Cytosolic DNA Sensing,” Nature Immunology 17, no. 10 (2016): 1142–1149. 27648547 10.1038/ni.3558

[42] A. Decout , J. D. Katz , S. Venkatraman , et al., “The cGAS–STING Pathway as a Therapeutic Target in Inflammatory Diseases,” Nature Reviews Immunology 21, no. 9 (2021): 548–569. 10.1038/s41577-021-00524-z PMC8029610 33833439

[43] B. Zhao , F. Du , P. Xu , et al., “A Conserved Plplrt/Sd Motif of Sting Mediates the Recruitment and Activation of TBK1,” Nature 569, no. 7758 (2019): 718–722. 31118511 10.1038/s41586-019-1228-x PMC6596994

[44] Q. Zhang , M. J. Lenardo , and D. Baltimore , “30 Years of Nf‐Κb: A Blossoming of Relevance to Human Pathobiology,” Cell 168, no. 1–2 (2017): 37–57. 28086098 10.1016/j.cell.2016.12.012 PMC5268070

[45] S. Liu , X. Cai , J. Wu , et al., “Phosphorylation of Innate Immune Adaptor Proteins MAVS, Sting, and Trif Induces Irf3 Activation,” Science 347 (2015): 6227. 10.1126/science.aaa2630 25636800

[46] T. Abe , G. N. Barber , and B. Williams , “Cytosolic‐DNA‐Mediated, Sting‐Dependent Proinflammatory Gene Induction Necessitates Canonical Nf‐Κb Activation Through TBK1,” Journal of Virology 88, no. 10 (2014): 5328–5341. 24600004 10.1128/JVI.00037-14 PMC4019140

[47] R. Fang , C. Wang , Q. Jiang , et al., “NEMO–IKKβ Are Essential for Irf3 and Nf‐Κb Activation in the cGAS–STING Pathway,” Journal of Immunology 199, no. 9 (2017): 3222–3233. 10.4049/jimmunol.1700699 28939760

[48] Q. Wang , Y. Yu , J. Zhuang , et al., “Demystifying the cGAS‐STING Pathway: Precision Regulation in the Tumor Immune Microenvironment,” Molecular Cancer 24, no. 1 (2025): 178. 40506729 10.1186/s12943-025-02380-0 PMC12160120

[49] S. Chattopadhyay , J. T. Marques , M. Yamashita , et al., “Viral Apoptosis Is Induced by Irf‐3‐Mediated Activation of Bax,” EMBO Journal 29, no. 10 (2010): 1762–1773. 20360684 10.1038/emboj.2010.50 PMC2876960

[50] S. J. Forrester , D. S. Kikuchi , M. S. Hernandes , et al., “Reactive Oxygen Species in Metabolic and Inflammatory Signaling,” Circulation Research 122, no. 6 (2018): 877–902. 29700084 10.1161/CIRCRESAHA.117.311401 PMC5926825

[51] C. Shi , Z. Wen , Y. Yang , et al., “Nad+ Metabolism and Therapeutic Strategies in Cardiovascular Diseases,” Atherosclerosis Plus 57 (2024): 1–12. 38974325 10.1016/j.athplu.2024.06.001 PMC11223091

[52] C. H. Patel and J. D. Powell , “More Tor: The Expanding Role of Mtor in Regulating Immune Responses,” Immunity 58, no. 7 (2025): 1629–1645. 40592340 10.1016/j.immuni.2025.06.010

[53] D. L. Mann , “The Emerging Field of Cardioimmunology: Past, Present and Foreseeable Future,” Circulation Research 134, no. 12 (2024): 1663–1680. 38843286 10.1161/CIRCRESAHA.123.323656 PMC11160976

[54] J. Liu , J. Zhou , Y. Luan , et al., “cGAS‐STING, Inflammasomes and Pyroptosis: An Overview of Crosstalk Mechanism of Activation and Regulation,” Cell Communication and Signaling 22, no. 1 (2024): 22. 38195584 10.1186/s12964-023-01466-w PMC10775518

[55] L. E. Newman and G. S. Shadel , “Mitochondrial DNA Release in Innate Immune Signaling,” Annual Review of Biochemistry 92 (2023): 299–332. 10.1146/annurev-biochem-032620-104401 PMC11058562 37001140

[56] M. Deberge , R. Chaudhary , S. Schroth , et al., “Immunometabolism at the Heart of Cardiovascular Disease,” JACC: Basic to Translational Science 8, no. 7 (2023): 884–904. 37547069 10.1016/j.jacbts.2022.12.010 PMC10401297

[57] A. Baysa , A. A. Maghazachi , K. L. Sand , et al., “Toll‐Like Receptor 9 Signaling After Myocardial Infarction: Role of P66shca Adaptor Protein,” Biochemical and Biophysical Research Communications 644 (2023): 70–78. 36634584 10.1016/j.bbrc.2022.12.085

[58] M. Lenz , A. Kiss , P. Haider , et al., “Short‐Term Toll‐Like Receptor 9 Inhibition Leads to Left Ventricular Wall Thinning After Myocardial Infarction,” ESC Heart Failure 10, no. 4 (2023): 2375–2385. 37190856 10.1002/ehf2.14403 PMC10375131

[59] H. Y. Kweon , E. J. Song , S. J. Jeong , et al., “Extracellular Peroxiredoxin 5 Exacerbates Atherosclerosis via the Tlr4/Myd88 Pathway,” Atherosclerosis 400 (2025): 119052. 39549462 10.1016/j.atherosclerosis.2024.119052

[60] C. Qian , D. Xu , J. Wang , et al., “Toll‐Like Receptor 2 Deficiency Ameliorates Obesity‐Induced Cardiomyopathy via Inhibiting Nf‐Κb Signaling Pathway,” International Immunopharmacology 128 (2024): 111551. 38278067 10.1016/j.intimp.2024.111551

[61] Y. Higashikuni , W. Liu , G. Numata , et al., “NLRP3 Inflammasome Activation Through Heart‐Brain Interaction Initiates Cardiac Inflammation and Hypertrophy During Pressure Overload,” Circulation 147, no. 4 (2023): 338–355. 36440584 10.1161/CIRCULATIONAHA.122.060860

[62] M. Yalcinkaya , W. Liu , L. A. Thomas , et al., “Brcc3‐Mediated NLRP3 Deubiquitylation Promotes Inflammasome Activation and Atherosclerosis in Tet2 Clonal Hematopoiesis,” Circulation 148, no. 22 (2023): 1764–1777. 37781816 10.1161/CIRCULATIONAHA.123.065344 PMC10872582

[63] W. He , J. Wei , X. Liu , et al., “Semaglutide Ameliorates Pressure Overload‐Induced Cardiac Hypertrophy by Improving Cardiac Mitophagy to Suppress the Activation of NLRP3 Inflammasome,” Scientific Reports 14, no. 1 (2024): 11824. 38782946 10.1038/s41598-024-62465-6 PMC11116553

[64] Y. Lei , J. J. Vanportfliet , Y. F. Chen , et al., “Cooperative Sensing of Mitochondrial DNA by Zbp1 and cGAS Promotes Cardiotoxicity,” Cell 186, no. 14 (2023): 3013–3032.e22. 37352855 10.1016/j.cell.2023.05.039 PMC10330843

[65] W. Luo , X. Zou , Y. Wang , et al., “Critical Role of the cGAS‐STING Pathway in Doxorubicin‐Induced Cardiotoxicity,” Circulation Research 132, no. 11 (2023): e223–e242. 37154056 10.1161/CIRCRESAHA.122.321587

[66] K. R. King , A. D. Aguirre , Y. X. Ye , et al., “Irf3 and Type I Interferons Fuel a Fatal Response to Myocardial Infarction,” Nature Medicine 23, no. 12 (2017): 1481–1487. 10.1038/nm.4428 PMC6477926 29106401

[67] Q. Zhang , H. Ding , Z. Dai , et al., “U‐Shaped Association Between Plasma Cyclic Guanosine Monophosphate‐Adenosine Monophosphate (cGAMP) Levels and Myocardial Infarction,” BMC Cardiovascular Disorders [Electronic Resource] 25, no. 1 (2025): 116. 39972291 10.1186/s12872-025-04543-9 PMC11837390

[68] Y. Xiong , Y. Leng , H. Tian , et al., “Decreased Mfn2 Activates the cGAS‐STING Pathway in Diabetic Myocardial Ischaemia‐Reperfusion by Triggering the Release of Mitochondrial DNA,” Cell Communication and Signaling 21, no. 1 (2023): 192. 37537600 10.1186/s12964-023-01216-y PMC10398939

[69] C. Zhang , H. Hao , Y. Wang , et al., “Intercellular Mitochondrial Component Transfer Triggers Ischemic Cardiac Fibrosis,” Science Bulletin (Beijing) 68, no. 16 (2023): 1784–1799. 10.1016/j.scib.2023.07.030 37517989

[70] D. Hu , Y. X. Cui , M. Y. Wu , et al., “Cytosolic DNA Sensor Cgas Plays an Essential Pathogenetic Role in Pressure Overload‐Induced Heart Failure,” American Journal of Physiology. Heart and Circulatory Physiology 318, no. 6 (2020): H1525–H1537. 32383996 10.1152/ajpheart.00097.2020

[71] C. An , F. Sun , C. Liu , et al., “Iqgap1 Promotes Mitochondrial Damage and Activation of the MtDNA Sensor cGAS‐STING Pathway to Induce Endothelial Cell Pyroptosis Leading to Atherosclerosis,” International Immunopharmacology 123 (2023): 110795. 37597406 10.1016/j.intimp.2023.110795

[72] H. Rui , H. Yu , K. Chi , et al., “Aldh2 Deficiency Augments Atherosclerosis Through the Usp14‐Cgas‐Dependent Polarization of Proinflammatory Macrophages,” Redox Biology 76 (2024): 103318. 39178733 10.1016/j.redox.2024.103318 PMC11388276

[73] M. Yan , Y. Li , Q. Luo , et al., “Mitochondrial Damage and Activation of the Cytosolic DNA Sensor cGAS‐STING Pathway Lead to Cardiac Pyroptosis and Hypertrophy in Diabetic Cardiomyopathy Mice,” Cell Death Discovery 8, no. 1 (2022): 258. 35538059 10.1038/s41420-022-01046-w PMC9091247

[74] Q. B. Lu , Y. Ding , Y. Liu , et al., “Metrnl Ameliorates Diabetic Cardiomyopathy via Inactivation of cGAS/STING Signaling Dependent on Lkb1/Ampk/Ulk1‐Mediated Autophagy,” Journal of Advanced Research 51 (2023): 161–179. 36334887 10.1016/j.jare.2022.10.014 PMC10491969

[75] Z. Kang , M. Yang , Y. Liu , et al., “Myocardial Mitochondrial Antiviral Signaling Protein Promotes Heart Ischemia‐Reperfusion Injury via Rig‐I Signaling in Mice,” Nature Communications 16, no. 1 (2025): 5101. 10.1038/s41467-025-60123-7 PMC12130335 40456736

[76] Y. Xiao , X. Hu , C. F. Rudolphi , et al., “The Innate Immune Receptor NLRX1 Is a Novel Required Modulator for Mptp Opening: Implications for Cardioprotection,” Basic Research in Cardiology 120, no. 4 (2025): 707–725. 40536683 10.1007/s00395-025-01124-x PMC12325489

[77] J. Y. Luo , C. K. Cheng , L. He , et al., “Endothelial Ucp2 Is a Mechanosensitive Suppressor of Atherosclerosis,” Circulation Research 131, no. 5 (2022): 424–441. 35899624 10.1161/CIRCRESAHA.122.321187 PMC9390236

[78] M. M. Tomczyk , K. G. Cheung , B. Xiang , et al., “Mitochondrial Sirtuin‐3 (Sirt3) Prevents Doxorubicin‐Induced Dilated Cardiomyopathy by Modulating Protein Acetylation and Oxidative Stress,” Circulation: Heart Failure 15, no. 5 (2022): e008547. 35418250 10.1161/CIRCHEARTFAILURE.121.008547 PMC9117478

[79] X. Zhang , T. D. Evans , S. Chen , et al., “Loss of Macrophage Mtorc2 Drives Atherosclerosis via Foxo1 and Il‐1β Signaling,” Circulation Research 133, no. 3 (2023): 200–219. 37350264 10.1161/CIRCRESAHA.122.321542 PMC10527041

[80] I. Martínez‐Reyes and N. S. Chandel , “Mitochondrial Tca Cycle Metabolites Control Physiology and Disease,” Nature Communications 11, no. 1 (2020): 102. 10.1038/s41467-019-13668-3 PMC6941980 31900386

[81] A. Hinton Jr. , S. M. Claypool , K. Neikirk , et al., “Mitochondrial Structure and Function in Human Heart Failure,” Circulation Research 135, no. 2 (2024): 372–396. 38963864 10.1161/CIRCRESAHA.124.323800 PMC11225798

[82] J. Sun , G. Gao , S. Wang , et al., “Decoding the Influence of Mitochondrial Ca^2+^ Regulation on Neurodegenerative Disease Progression,” Mitochondrial Communications 3 (2025): 1–15.

[83] D. F. Dai , Y. A. Chiao , D. J. Marcinek , et al., “Mitochondrial Oxidative Stress in Aging and Healthspan,” Longevity Healthspan 3 (2014): 6. 24860647 10.1186/2046-2395-3-6 PMC4013820

[84] M. Zhang , T. Zhang , R. Zou , et al., “Mitochondrial Quality Control as a Therapeutic Target in Cardiovascular Disease: Mechanistic Insights and Future Directions,” Journal of Translational Internal Medicine 13, no. 3 (2025): 211–240. 40896290 10.1515/jtim-2025-0030 PMC12392085

[85] D. Xia , Y. Liu , P. Wu , et al., “Current Advances of Mitochondrial Dysfunction and Cardiovascular Disease and Promising Therapeutic Strategies,” American Journal of Pathology 193, no. 10 (2023): 1485–1500. 37481069 10.1016/j.ajpath.2023.06.013

[86] Z. Qin , X. Zhu , Y. Shen , et al., “An Emerging Role of Mitochondrial Quality Control in Bone Metabolism: From Molecular Mechanisms to Targeted Therapeutic Interventions,” Cellular and Molecular Life Sciences 82, no. 1 (2025): 291. 40728722 10.1007/s00018-025-05802-w PMC12307276

[87] A. Roca‐Portoles and S. W. G. Tait , “Mitochondrial Quality Control: From Molecule to Organelle,” Cellular and Molecular Life Sciences 78, no. 8 (2021): 3853–3866. 33782711 10.1007/s00018-021-03775-0 PMC8106605

[88] B. H. Liu , C. Z. Xu , Y. Liu , et al., “Mitochondrial Quality Control in Human Health and Disease,” Military Medical Research 11, no. 1 (2024): 32. 38812059 10.1186/s40779-024-00536-5 PMC11134732

[89] K. H. Kim and C. B. Lee , “Socialized Mitochondria: Mitonuclear Crosstalk in Stress,” Experimental & Molecular Medicine 56, no. 5 (2024): 1033–1042. 38689084 10.1038/s12276-024-01211-4 PMC11148012

[90] S. Pickles , P. Vigié , and R. J. Youle , “Mitophagy and Quality Control Mechanisms in Mitochondrial Maintenance,” Current Biology 28, no. 4 (2018): R170–R185. 29462587 10.1016/j.cub.2018.01.004 PMC7255410

[91] G. Ashrafi and T. L. Schwarz , “The Pathways of Mitophagy for Quality Control and Clearance of Mitochondria,” Cell Death and Differentiation 20, no. 1 (2013): 31–42. 22743996 10.1038/cdd.2012.81 PMC3524633

[92] A. Li , M. Gao , B. Liu , et al., “Mitochondrial Autophagy: Molecular Mechanisms and Implications for Cardiovascular Disease,” Cell Death & Disease 13, no. 5 (2022): 444. 35534453 10.1038/s41419-022-04906-6 PMC9085840

[93] X. Zhou , J. Wang , L. Yu , et al., “Mitophagy and cGAS‐STING Crosstalk in Neuroinflammation,” Acta Pharmaceutica Sinica B 14, no. 8 (2024): 3327–3361. 39220869 10.1016/j.apsb.2024.05.012 PMC11365416

[94] P. Ge , V. L. Dawson , and T. M. Dawson , “Pink1 and Parkin Mitochondrial Quality Control: A Source of Regional Vulnerability in Parkinson's Disease,” Molecular Neurodegeneration 15, no. 1 (2020): 20. 32169097 10.1186/s13024-020-00367-7 PMC7071653

[95] Z. Yang , S. R. Yoshii , Y. Sakai , et al., “Autophagy Adaptors Mediate Parkin‐Dependent Mitophagy by Forming Sheet‐Like Liquid Condensates,” EMBO Journal 43, no. 22 (2024): 5613–5634. 39420095 10.1038/s44318-024-00272-5 PMC11574277

[96] E. Adriaenssens , T. N. Nguyen , J. Sawa‐Makarska , et al., “Control of Mitophagy Initiation and Progression by the TBK1 Adaptors Nap1 and Sintbad,” Nature Structural & Molecular Biology 31, no. 11 (2024): 1717–1731. 10.1038/s41594-024-01338-y PMC11564117 38918639

[97] M. Zachari and N. T. Ktistakis , “Mammalian Mitophagosome Formation: A Focus on the Early Signals and Steps,” Frontiers in Cell and Developmental Biology 8 (2020): 171. 32258042 10.3389/fcell.2020.00171 PMC7093328

[98] W. Zhang , “The Mitophagy Receptor Fun14 Domain‐Containing 1 (Fundc1): A Promising Biomarker and Potential Therapeutic Target of Human Diseases,” Genes & Diseases 8, no. 5 (2021): 640–654. 34291135 10.1016/j.gendis.2020.08.011 PMC8278526

[99] X. Wu , F. H. Wu , Q. Wu , et al., “Phylogenetic and Molecular Evolutionary Analysis of Mitophagy Receptors Under Hypoxic Conditions,” Frontiers in Physiology 8 (2017): 539. 28798696 10.3389/fphys.2017.00539 PMC5526904

[100] L. Liu , K. Sakakibara , Q. Chen , et al., “Receptor‐Mediated Mitophagy in Yeast and Mammalian Systems,” Cell Research 24, no. 7 (2014): 787–795. 24903109 10.1038/cr.2014.75 PMC4085769

[101] R. A. Hanna , M. N. Quinsay , A. M. Orogo , et al., “Microtubule‐Associated Protein 1 Light Chain 3 (LC3) Interacts With Bnip3 Protein to Selectively Remove Endoplasmic Reticulum and Mitochondria via Autophagy,” Journal of Biological Chemistry 287, no. 23 (2012): 19094–19104. 22505714 10.1074/jbc.M111.322933 PMC3365942

[102] M. Matsushima , T. Fujiwara , E. Takahashi , et al., “Isolation, Mapping, and Functional Analysis of a Novel Human cDNA (Bnip3l) Encoding a Protein Homologous to Human Nip3,” Genes, Chromosomes & Cancer 21, no. 3 (1998): 230–235. 9523198

[103] J. Zhang and P. A. Ney , “Role of Bnip3 and Nix in Cell Death, Autophagy, and Mitophagy,” Cell Death and Differentiation 16, no. 7 (2009): 939–946. 19229244 10.1038/cdd.2009.16 PMC2768230

[104] R. Deng , Y. Wang , Y. Bu , et al., “Bnip3 Mediates the Different Adaptive Responses of Fibroblast‐Like Synovial Cells to Hypoxia in Patients With Osteoarthritis and Rheumatoid Arthritis,” Molecular Medicine 28, no. 1 (2022): 64. 35690741 10.1186/s10020-022-00490-9 PMC9188199

[105] Z. J. Fu , Z. Y. Wang , L. Xu , et al., “Hif‐1Alpha‐Bnip3‐Mediated Mitophagy in Tubular Cells Protects Against Renal Ischemia/Reperfusion Injury,” Redox Biology 36 (2020): 101671. 32829253 10.1016/j.redox.2020.101671 PMC7452120

[106] I. Novak , V. Kirkin , D. G. Mcewan , et al., “Nix Is a Selective Autophagy Receptor for Mitochondrial Clearance,” EMBO Reports 11, no. 1 (2010): 45–51. 20010802 10.1038/embor.2009.256 PMC2816619

[107] R. L. Schweers , J. Zhang , M. S. Randall , et al., “Nix Is Required for Programmed Mitochondrial Clearance During Reticulocyte Maturation,” PNAS 104, no. 49 (2007): 19500–19505. 18048346 10.1073/pnas.0708818104 PMC2148318

[108] M. Yazdankhah , S. Ghosh , P. Shang , et al., “Bnip3l‐Mediated Mitophagy Is Required for Mitochondrial Remodeling During the Differentiation of Optic Nerve Oligodendrocytes,” Autophagy 17, no. 10 (2021): 3140–3159. 33404293 10.1080/15548627.2020.1871204 PMC8526037

[109] C. Wang , X. Dai , S. Wu , et al., “Fundc1‐Dependent Mitochondria‐Associated Endoplasmic Reticulum Membranes Are Involved in Angiogenesis and Neoangiogenesis,” Nature Communications 12, no. 1 (2021): 2616. 10.1038/s41467-021-22771-3 PMC8110587 33972548

[110] L. Liu , D. Feng , G. Chen , et al., “Mitochondrial Outer‐Membrane Protein Fundc1 Mediates Hypoxia‐Induced Mitophagy in Mammalian Cells,” Nature Cell Biology 14, no. 2 (2012): 177–185. 22267086 10.1038/ncb2422

[111] W. Wu , C. Lin , K. Wu , et al., “Fundc1 Regulates Mitochondrial Dynamics at the Er‐Mitochondrial Contact Site Under Hypoxic Conditions,” EMBO Journal 35, no. 13 (2016): 1368–1384. 27145933 10.15252/embj.201593102 PMC4864280

[112] M. Chen , Z. Chen , Y. Wang , et al., “Mitophagy Receptor Fundc1 Regulates Mitochondrial Dynamics and Mitophagy,” Autophagy 12, no. 4 (2016): 689–702. 27050458 10.1080/15548627.2016.1151580 PMC4836026

[113] T. Murakawa , O. Yamaguchi , A. Hashimoto , et al., “Bcl‐2‐Like Protein 13 Is a Mammalian Atg32 Homologue That Mediates Mitophagy and Mitochondrial Fragmentation,” Nature Communications 6 (2015): 7527. 10.1038/ncomms8527 PMC4501433 26146385

[114] K. Otsu , T. Murakawa , and O. Yamaguchi , “Bcl2l13 Is a Mammalian Homolog of the Yeast Mitophagy Receptor Atg32,” Autophagy 11, no. 10 (2015): 1932–1933. 26506896 10.1080/15548627.2015.1084459 PMC4824574

[115] J. Wang , A. Chen , Z. Xue , et al., “Bcl2l13 Promotes Mitophagy Through Dnm1l‐Mediated Mitochondrial Fission in Glioblastoma,” Cell Death & Disease 14, no. 9 (2023): 585. 37660127 10.1038/s41419-023-06112-4 PMC10475114

[116] P. Y. Bi , S. A. Killackey , L. Schweizer , et al., “NLRX1: Versatile Functions of a Mitochondrial Nlr Protein That Controls Mitophagy,” Biomedical Journal 47, no. 1 (2024): 100635. 37574163 10.1016/j.bj.2023.100635 PMC10837482

[117] S. A. Killackey , Y. Bi , F. Soares , et al., “Mitochondrial Protein Import Stress Regulates the Lc3 Lipidation Step of Mitophagy Through NLRX1 and Rrbp1,” Molecular Cell 82, no. 15 (2022): 2815–2831.e5. 35752171 10.1016/j.molcel.2022.06.004

[118] M. O. Aguilera , E. Robledo , M. Melani , et al., “Fkbp8 Is a Novel Molecule That Participates in the Regulation of the Autophagic Pathway,” Biochimica et Biophysica Acta ‐ Molecular Cell Research 1869, no. 5 (2022): 119212. 35090967 10.1016/j.bbamcr.2022.119212

[119] Z. Bhujabal , A. B. Birgisdottir , E. Sjottem , et al., “Fkbp8 Recruits Lc3a to Mediate Parkin‐Independent Mitophagy,” EMBO Reports 18, no. 6 (2017): 947–961. 28381481 10.15252/embr.201643147 PMC5452039

[120] Y. Wei , W. C. Chiang , R. Sumpter Jr. , et al., “Prohibitin 2 Is an Inner Mitochondrial Membrane Mitophagy Receptor,” Cell 168, no. 1–2 (2017): 224–238.e10. 28017329 10.1016/j.cell.2016.11.042 PMC5235968

[121] M. Vara‐Perez , B. Felipe‐Abrio , and P. Agostinis , “Mitophagy in Cancer: A Tale of Adaptation,” Cells 8, no. 5 (2019): 493. 31121959 10.3390/cells8050493 PMC6562743

[122] C. Yan , L. Gong , L. Chen , et al., “Phb2 (Prohibitin 2) Promotes Pink1‐Prkn/Parkin‐Dependent Mitophagy by the Parl‐Pgam5‐Pink1 Axis,” Autophagy 16, no. 3 (2020): 419–434. 31177901 10.1080/15548627.2019.1628520 PMC6999623

[123] M. N. Iriondo , A. Etxaniz , Y. R. Varela , et al., “Lc3 Subfamily in Cardiolipin‐Mediated Mitophagy: A Comparison of the Lc3a, Lc3b and Lc3c Homologs,” Autophagy 18, no. 12 (2022): 2985–3003. 35414338 10.1080/15548627.2022.2062111 PMC9673933

[124] Z. Jiang , T. Shen , H. Huynh , et al., “Cardiolipin Regulates Mitochondrial Ultrastructure and Function in Mammalian Cells,” Genes (Basel) 13, no. 10 (2022): 1889. 36292774 10.3390/genes13101889 PMC9601307

[125] X. Yang , R. Zhang , K. Nakahira , et al., “Mitochondrial DNA Mutation, Diseases, and Nutrient‐Regulated Mitophagy,” Annual Review of Nutrition 39 (2019): 201–226. 10.1146/annurev-nutr-082018-124643 31433742

[126] Z. Anton , A. Landajuela , J. H. Hervas , et al., “Human Atg8‐Cardiolipin Interactions in Mitophagy: Specific Properties of Lc3b, Gabarapl2 and Gabarap,” Autophagy 12, no. 12 (2016): 2386–2403. 27764541 10.1080/15548627.2016.1240856 PMC5172498

[127] K. H. Fisher‐Wellman , J. T. Hagen , P. D. Neufer , et al., “On the Nature of Ceramide‐Mitochondria Interactions—Dissection Using Comprehensive Mitochondrial Phenotyping,” Cell Signalling 78 (2021): 109838. 33212155 10.1016/j.cellsig.2020.109838 PMC7775270

[128] R. D. Sentelle , C. E. Senkal , W. Jiang , et al., “Ceramide Targets Autophagosomes to Mitochondria and Induces Lethal Mitophagy,” Nature Chemical Biology 8, no. 10 (2012): 831–838. 22922758 10.1038/nchembio.1059 PMC3689583

[129] E. Marques , R. Kramer , and D. G. Ryan , “Multifaceted Mitochondria in Innate Immunity,” NPJ Metabolic Health and Disease 2, no. 1 (2024): 6. 38812744 10.1038/s44324-024-00008-3 PMC11129950

[130] J. Yoon , S. Kim , M. Lee , et al., “Mitochondrial Nucleic Acids in Innate Immunity and Beyond,” Experimental & Molecular Medicine 55, no. 12 (2023): 2508–2518. 38036728 10.1038/s12276-023-01121-x PMC10766607

[131] L. Giordano , S. A. Ware , C. J. Lagranha , et al., “Mitochondrial DNA Signals Driving Immune Responses: Why, How, Where?,” Cell Communication and Signaling 23, no. 1 (2025): 192. 40264103 10.1186/s12964-025-02042-0 PMC12012978

[132] A. Koenig and I. A. Buskiewicz‐Koenig , “Redox Activation of Mitochondrial Damps and the Metabolic Consequences for Development of Autoimmunity,” Antioxidants & Redox Signaling 36, no. 7–9 (2022): 441–461. 35352943 10.1089/ars.2021.0073 PMC8982130

[133] M.‐M. Hu and H.‐B. Shu , “Mitochondrial DNA‐Triggered Innate Immune Response: Mechanisms and Diseases,” Cellular & Molecular Immunology 20, no. 12 (2023): 1403–1412. 37932533 10.1038/s41423-023-01086-x PMC10687031

[134] S. Antonucci , F. Di Lisa , and N. Kaludercic , “Mitochondrial Reactive Oxygen Species in Physiology and Disease,” Cell Calcium 94 (2021): 102344. 33556741 10.1016/j.ceca.2020.102344

[135] P. Pelegrin , “P2×7 Receptor and the NLRP3 Inflammasome: Partners in Crime,” Biochemical Pharmacology 187 (2021): 114385. 33359010 10.1016/j.bcp.2020.114385

[136] Y. Zhuang , M. L. Yu , and S. F. Lu , “Purinergic Signaling in Myocardial Ischemia‐Reperfusion Injury,” Purinergic Signal 19, no. 1 (2023): 229–243. 35254594 10.1007/s11302-022-09856-4 PMC9984618

[137] M. Pizzuto and P. Pelegrin , “Cardiolipin in Immune Signaling and Cell Death,” Trends in Cell Biology 30, no. 11 (2020): 892–903. 33011017 10.1016/j.tcb.2020.09.004

[138] A. E. Atici , T. R. Crother , and M. Noval Rivas , “Mitochondrial Quality Control in Health and Cardiovascular Diseases,” Frontiers in Cell and Developmental Biology 11 (2023): 1290046. 38020895 10.3389/fcell.2023.1290046 PMC10657886

[139] Y. Luan , Y. Luan , Y. Jiao , et al., “Broadening Horizons: Exploring Mtdamps as a Mechanism and Potential Intervention Target in Cardiovascular Diseases,” Aging and Disease 15, no. 6 (2024): 2395–2416. 10.14336/AD.2023.1130 PMC11567272 38270118

[140] M. P. Murphy and L. A. J. O'neill , “A Break in Mitochondrial Endosymbiosis as a Basis for Inflammatory Diseases,” Nature 626, no. 7998 (2024): 271–279. 38326590 10.1038/s41586-023-06866-z

[141] J. I. Jiménez‐Loygorri , B. Villarejo‐Zori , Á. Viedma‐Poyatos , et al., “Mitophagy Curtails Cytosolic mtDNA‐Dependent Activation of Cgas/Sting Inflammation During Aging,” Nature Communications 15, no. 1 (2024): 830. 10.1038/s41467-024-45044-1 PMC10821893 38280852

[142] C. Rocca , T. Soda , E. M. De Francesco , et al., “Mitochondrial Dysfunction at the Crossroad of Cardiovascular Diseases and Cancer,” Journal of Translational Medicine 21, no. 1 (2023): 635. 37726810 10.1186/s12967-023-04498-5 PMC10507834

[143] M. K. Torp , J. Vaage , and K. O. Stensløkken , “Mitochondria‐Derived Damage‐Associated Molecular Patterns and Inflammation in the Ischemic‐Reperfused Heart,” Acta Physiologica (Oxford, England) 237, no. 3 (2023): e13920. 36617670 10.1111/apha.13920

[144] W. Chen , M. Ma , Y. Song , et al., “Exercise Attenuates Myocardial Ischemia‐Reperfusion Injury by Regulating Endoplasmic Reticulum Stress and Mitophagy Through M(2) Acetylcholine Receptor,” Antioxidants & Redox Signaling 40, no. 4–6 (2024): 209–221. 37294203 10.1089/ars.2022.0168

[145] Q. Xu , S. Liu , Q. Gong , et al., “Notch1 Protects Against Ischemic‐Reperfusion Injury by Suppressing Pten‐Pink1‐Mediated Mitochondrial Dysfunction and Mitophagy,” Cells 12, no. 1 (2022): 137. 36611931 10.3390/cells12010137 PMC9818205

[146] Z. Chen , T. Liu , H. Yuan , et al., “Bioinformatics Integration Reveals Key Genes Associated With Mitophagy in Myocardial Ischemia‐Reperfusion Injury,” BMC Cardiovascular Disorders [Electronic Resource] 24, no. 1 (2024): 183. 38539069 10.1186/s12872-024-03834-x PMC10967080

[147] T. L. Lee , W. C. Shen , Y. C. Chen , et al., “Mir221‐ and Mir222‐Enriched Adsc‐Exosomes Mitigate Pm Exposure‐Exacerbated Cardiac Ischemia‐Reperfusion Injury Through the Modulation of the Bnip3‐Map1lc3b‐Bbc3/Puma Pathway,” Autophagy 21, no. 2 (2025): 374–393. 39245438 10.1080/15548627.2024.2395799 PMC11760231

[148] Z. Qiu , H. Ming , S. Lei , et al., “Roles of Hdac3‐Orchestrated Circadian Clock Gene Oscillations in Diabetic Rats Following Myocardial Ischaemia/Reperfusion Injury,” Cell Death & Disease 12, no. 1 (2021): 43. 33414413 10.1038/s41419-020-03295-y PMC7791027

[149] Y. Zhang , H. Nie , S. Li , et al., “Carbon Monoxide‐Saturated Polymerized Placenta Hemoglobin Optimizes Mitochondrial Function and Protects Heart Against Ischemia‐Reperfusion Injury,” Journal of Cardiovascular Pharmacology 77, no. 6 (2021): 814–821. 34001725 10.1097/FJC.0000000000001022

[150] Q. Wang , X. Xue , P. Wang , et al., “Angiotensin 1 Peptide‐Conjugated Cdse/Zns Quantum Dots for Cardiac‐Specific Hydrogen Sulfide Targeted Therapy in Myocardial Ischemia‐Reperfusion Injury,” Frontiers in Pharmacology 15 (2024): 1435282. 39415837 10.3389/fphar.2024.1435282 PMC11479923

[151] H. Li , S. Qin , Q. Liang , et al., “Exercise Training Enhances Myocardial Mitophagy and Improves Cardiac Function via Irisin/Fndc5‐Pink1/Parkin Pathway in Mi Mice,” Biomedicines 9, no. 6 (2021): 701. 34205641 10.3390/biomedicines9060701 PMC8234442

[152] M. Sun , W. Zhang , Y. Bi , et al., “Ndp52 Protects Against Myocardial Infarction‐Provoked Cardiac Anomalies Through Promoting Autophagosome‐Lysosome Fusion via Recruiting TBK1 and Rab7,” Antioxidants & Redox Signaling 36, no. 16–18 (2022): 1119–1135. 34382418 10.1089/ars.2020.8253

[153] B. S. Wu , H. Q. Xiang , Y. W. Yu , et al., “3,4‐Benzo[a]Pyrene Aggravates Myocardial Infarction Injury by Activating NLRP3‐Related Pyroptosis Through Pink1/Parkin‐Mitophagy‐Mptp Opening Axis,” International Immunopharmacology 122 (2023): 110481. 37390647 10.1016/j.intimp.2023.110481

[154] T. Xin , W. Lv , D. Liu , et al., “Opa1 Reduces Hypoxia‐Induced Cardiomyocyte Death by Improving Mitochondrial Quality Control,” Frontiers in Cell and Developmental Biology 8 (2020): 853. 32984338 10.3389/fcell.2020.00853 PMC7483501

[155] J. Hu , T. Liu , F. Fu , et al., “Omentin1 Ameliorates Myocardial Ischemia‐Induced Heart Failure via Sirt3/Foxo3a‐Dependent Mitochondrial Dynamical Homeostasis and Mitophagy,” Journal of Translational Medicine 20, no. 1 (2022): 447. 36192726 10.1186/s12967-022-03642-x PMC9531426

[156] C. Guo , R. Y. Wu , J. H. Dou , et al., “Mitophagy‐Dependent Cardioprotection of Resistance Training on Heart Failure,” Journal of Applied Physiology (1985) 135, no. 6 (2023): 1390–1401. 10.1152/japplphysiol.00674.2023 37942531

[157] Q. Li , Y. Liu , Q. Huang , et al., “Hypoxia Acclimation Protects Against Heart Failure Postacute Myocardial Infarction via Fundc1‐Mediated Mitophagy,” Oxidative Medicine and Cellular Longevity 2022 (2022): 8192552. 35422895 10.1155/2022/8192552 PMC9005280

[158] Y. Jin , Y. Liu , L. Xu , et al., “Novel Role for Caspase 1 Inhibitor Vx765 in Suppressing NLRP3 Inflammasome Assembly and Atherosclerosis via Promoting Mitophagy and Efferocytosis,” Cell Death & Disease 13, no. 5 (2022): 512. 35641492 10.1038/s41419-022-04966-8 PMC9156694

[159] P. Li , J. Wang , X. Zhao , et al., “Pten Inhibition Attenuates Endothelial Cell Apoptosis in Coronary Heart Disease via Modulating the Ampk‐Creb‐Mfn2‐Mitophagy Signaling Pathway,” Journal of Cellular Physiology 235, no. 5 (2020): 4878–4889. 31654396 10.1002/jcp.29366

[160] A. Gao , J. Zou , Z. Mao , et al., “Sumo2‐Mediated Sumoylation of Sh3glb1 Promotes Ionizing Radiation‐Induced Hypertrophic Cardiomyopathy Through Mitophagy Activation,” European Journal of Pharmacology 924 (2022): 174980. 35487252 10.1016/j.ejphar.2022.174980

[161] Y. Tan , Y. Zhang , J. He , et al., “Dual Specificity Phosphatase 1 Attenuates Inflammation‐Induced Cardiomyopathy by Improving Mitophagy and Mitochondrial Metabolism,” Molecular Metabolism 64 (2022): 101567. 35944900 10.1016/j.molmet.2022.101567 PMC9418987

[162] E. Li , X. Li , J. Huang , et al., “Bmal1 Regulates Mitochondrial Fission and Mitophagy Through Mitochondrial Protein Bnip3 and Is Critical in the Development of Dilated Cardiomyopathy,” Protein Cell 11, no. 9 (2020): 661–679. 32277346 10.1007/s13238-020-00713-x PMC7452999

[163] Y. Sun , F. Lu , X. Yu , et al., “Exogenous H(2)S Promoted Usp8 Sulfhydration to Regulate Mitophagy in the Hearts of Db/Db Mice,” Aging and Disease 11, no. 2 (2020): 269–285. 32257541 10.14336/AD.2019.0524 PMC7069468

[164] W. Huang , A. Lou , J. Wang , et al., “Tmbim1 Ameliorates Sepsis‐Induced Cardiac Dysfunction by Promoting Parkin‐Mediated Mitophagy,” FASEB Journal 39, no. 4 (2025): e70397. 39937566 10.1096/fj.202402599RR

[165] Z. Pei , Y. Liu , S. Liu , et al., “Fundc1 Insufficiency Sensitizes High Fat Diet Intake‐Induced Cardiac Remodeling and Contractile Anomaly Through Acsl4‐Mediated Ferroptosis,” Metabolism 122 (2021): 154840. 34331963 10.1016/j.metabol.2021.154840

[166] Z. Song , H. Song , D. Liu , et al., “Overexpression of Mfn2 Alleviates Sorafenib‐Induced Cardiomyocyte Necroptosis via the Mam‐Camkiiδ Pathway In Vitro and In Vivo,” Theranostics 12, no. 3 (2022): 1267–1285. 35154486 10.7150/thno.65716 PMC8771548

[167] S. Lei , Y. Feng , P. Huang , et al., “Ophiopogonin D'‐Induced Mitophagy and Mitochondrial Damage Are Associated With Dysregulation of the Pink1/Parkin Signaling Pathway in Ac16 Cells,” Toxicology 477 (2022): 153275. 35905946 10.1016/j.tox.2022.153275

[168] M. O. El‐Derany and S. G. Abdelhamid , “Bone Marrow Mesenchymal Stem Cells and Their Derived Extracellular Vesicles Attenuate Non‐Alcoholic Steatohepatitis‐Induced Cardiotoxicity via Modulating Cardiac Mechanisms,” Life (Basel) 12, no. 3 (2022): 355. 35330106 10.3390/life12030355 PMC8952775

[169] A. Chowdhury , S. Witte , and A. Aich , “Role of Mitochondrial Nucleic Acid Sensing Pathways in Health and Patho‐Physiology,” Frontiers in Cell and Developmental Biology 10 (2022): 796066. 35223833 10.3389/fcell.2022.796066 PMC8873532

[170] F. Civril , T. Deimling , C. C. De Oliveira Mann , et al., “Structural Mechanism of Cytosolic DNA Sensing by cGAS,” Nature 498, no. 7454 (2013): 332–337. 23722159 10.1038/nature12305 PMC3768140

[171] C. Ritchie , J. A. Carozza , and L. Li , “Biochemistry, Cell Biology, and Pathophysiology of the Innate Immune cGAS–cGAMP–STING Pathway,” Annual Review of Biochemistry 91, no. 1 (2022): 599–628. 10.1146/annurev-biochem-040320-101629 35287475

[172] H. Kwak , E. Lee , and R. Karki , “DNA Sensors in Metabolic and Cardiovascular Diseases: Molecular Mechanisms and Therapeutic Prospects,” Immunological Reviews 329, no. 1 (2025): e13382. 39158380 10.1111/imr.13382 PMC11744256

[173] R. Kitazume‐Taneike , M. Taneike , S. Omiya , et al., “Ablation of Toll‐Like Receptor 9 Attenuates Myocardial Ischemia/Reperfusion Injury in Mice,” Biochemical and Biophysical Research Communications 515, no. 3 (2019): 442–447. 31160091 10.1016/j.bbrc.2019.05.150 PMC6590932

[174] Q. Wang , Y. Sun , T. Y. Li , et al., “Mitophagy in the Pathogenesis and Management of Disease,” Cell Research 36, no. 1 (2026): 11–37. 41486294 10.1038/s41422-025-01203-7 PMC12765899

[175] L. K. Billingham , J. S. Stoolman , K. Vasan , et al., “Mitochondrial Electron Transport Chain Is Necessary for NLRP3 Inflammasome Activation,” Nature Immunology 23, no. 5 (2022): 692–704. 35484407 10.1038/s41590-022-01185-3 PMC9098388

[176] B. S. Saller , S. Wöhrle , L. Fischer , et al., “Acute Suppression of Mitochondrial Atp Production Prevents Apoptosis and Provides an Essential Signal for NLRP3 Inflammasome Activation,” Immunity 58, no. 1 (2025): 90–107.e11. 39571574 10.1016/j.immuni.2024.10.012

[177] H. Xian , K. Watari , E. Sanchez‐Lopez , et al., “Oxidized DNA Fragments Exit Mitochondria via Mptp‐ and Vdac‐Dependent Channels to Activate NLRP3 Inflammasome and Interferon Signaling,” Immunity 55, no. 8 (2022): 1370–1385.e8. 35835107 10.1016/j.immuni.2022.06.007 PMC9378606

[178] A. Cabral , J. E. Cabral , A. Wang , et al., “Differential Binding of NLRP3 to Non‐Oxidized and Ox‐mtDNA Mediates NLRP3 Inflammasome Activation,” Communications Biology 6, no. 1 (2023): 578. 37253813 10.1038/s42003-023-04817-y PMC10229695

[179] L. Luo , F. Wang , X. Xu , et al., “Stat3 Promotes NLRP3 Inflammasome Activation by Mediating NLRP3 Mitochondrial Translocation,” Experimental & Molecular Medicine 56, no. 9 (2024): 1980–1990. 39218978 10.1038/s12276-024-01298-9 PMC11446920

[180] A. Akbal , A. Dernst , M. Lovotti , et al., “How Location and Cellular Signaling Combine to Activate the NLRP3 Inflammasome,” Cellular & Molecular Immunology 19, no. 11 (2022): 1201–1214. 36127465 10.1038/s41423-022-00922-w PMC9622870

[181] W. Fu , S. C. Liu , T. X. Xu , et al., “Emodin Inhibits NLRP3 Inflammasome Activation and Protects Against Sepsis via Promoting Fundc1‐Mediated Mitophagy,” International Journal of Biological Sciences 21, no. 8 (2025): 3631–3648. 40520006 10.7150/ijbs.110904 PMC12160860

[182] C. De Torre‐Minguela , A. I. Gómez , I. Couillin , et al., “Gasdermins Mediate Cellular Release of Mitochondrial DNA During Pyroptosis and Apoptosis,” FASEB Journal 35, no. 8 (2021): e21757. 34233045 10.1096/fj.202100085R

[183] M. Wu , C. Yu , F. Wen , et al., “NLRP3 Inflammasome Inhibits Mitophagy During the Progression of Temporal Lobe Epilepsy,” Scientific Reports 15, no. 1 (2025): 16341. 40348802 10.1038/s41598-025-01087-y PMC12065917

[184] C. Li , Y. Zhu , W. Liu , et al., “Impaired Mitophagy Causes Mitochondrial DNA Leakage and Sting Activation in Ultraviolet B‐Irradiated Human Keratinocytes Hacat,” Archives of Biochemistry and Biophysics 737 (2023): 109553. 36842493 10.1016/j.abb.2023.109553

[185] D. Liu , H. Wu , C. Wang , et al., “Sting Directly Activates Autophagy to Tune the Innate Immune Response,” Cell Death and Differentiation 26, no. 9 (2018): 1735–1749. 30568238 10.1038/s41418-018-0251-z PMC6748081

[186] X. Gui , H. Yang , T. Li , et al., “Autophagy Induction via Sting Trafficking Is a Primordial Function of the cGAS Pathway,” Nature 567, no. 7747 (2019): 262–266. 30842662 10.1038/s41586-019-1006-9 PMC9417302

[187] B. Lv , W. A. Dion , H. Yang , et al., “A TBK1‐Independent Primordial Function of Sting in Lysosomal Biogenesis,” Molecular Cell 84, no. 20 (2024): 3979–3996.e9. 39423796 10.1016/j.molcel.2024.08.026 PMC11490688

[188] S. Guha and N. Laguette , “Sting Signalling as a Mediator Between Lipid Metabolism and Innate Immunity,” Nature Reviews Molecular Cell Biology 27, no. 6 (2026): 413–415. 10.1038/s41580-026-00967-z 41912686

[189] C. Garlanda , I. Di Ceglie , and S. Jaillon , “Il‐1 Family Cytokines in Inflammation and Immunity,” Cellular & Molecular Immunology 22, no. 11 (2025): 1345–1362. 41087719 10.1038/s41423-025-01358-8 PMC12575874

[190] Y. Zheng , K. Wei , P. Jiang , et al., “Macrophage Polarization in Rheumatoid Arthritis: Signaling Pathways, Metabolic Reprogramming, and Crosstalk With Synovial Fibroblasts,” Frontiers in Immunology 15 (2024): 1394108. 38799455 10.3389/fimmu.2024.1394108 PMC11116671

[191] A. J. Hruby and R. Higuchi‐Sanabria , “Mitochondrial Dysfunction in Cellular Senescence: A Bridge to Neurodegenerative Disease,” NPJ Aging 11, no. 1 (2025): 99. 41402339 10.1038/s41514-025-00291-4 PMC12708852

[192] A. Mongelli , A. Mengozzi , M. Geiger , et al., “Mitochondrial Epigenetics in Aging and Cardiovascular Diseases,” Frontiers in Cardiovascular Medicine 10 (2023): 1204483. 37522089 10.3389/fcvm.2023.1204483 PMC10382027

[193] W. Chen , Z. Zhao , Z. Geng , et al., “Advances in Mitochondria‐Nucleus Crosstalk in Septic Cardiomyopathy,” Cell Biology and Toxicology 41, no. 1 (2025): 136. 41051583 10.1007/s10565-025-10090-y PMC12500838

[194] Y. You and Z. Wang , “Roles of Sirt3 in Aging and Aging‐Related Diseases,” International Journal of Biological Sciences 21, no. 11 (2025): 5135–5163. 40860195 10.7150/ijbs.115518 PMC12374834

[195] Z. Lin , X. Lu , G. Xu , et al., “The Mitochondrial E3 Ligase Mapl Sumoylates Drp1 to Facilitate Mitochondrial Fission in Intervertebral Disc Degeneration,” Bone Research 13, no. 1 (2025): 72. 40796734 10.1038/s41413-025-00449-6 PMC12343876

[196] J. Willemsen , M.‐T. Neuhoff , T. Hoyler , et al., “Tnf Leads to mtDNA Release and cGAS/STING‐Dependent Interferon Responses That Support Inflammatory Arthritis,” Cell Reports 37, no. 6 (2021): 109977. 34758308 10.1016/j.celrep.2021.109977

[197] H. Xian and M. Karin , “Oxidized Mitochondrial DNA: A Protective Signal Gone Awry,” Trends in Immunology 44, no. 3 (2023): 188–200. 36739208 10.1016/j.it.2023.01.006 PMC12045651

[198] Z. Chai , Y. Zhou , S. Hossain , et al., “Cardiolipin's Multifaceted Role in Immune Response: A Focus on Interacting Proteins,” Frontiers in Immunology 16 (2025): 1680326. 41438747 10.3389/fimmu.2025.1680326 PMC12719303

[199] L. Ye , X. Fu , and Q. Li , “Mitochondrial Quality Control in Health and Disease,” MedComm 6, no. 8 (2025): e70319. 40821693 10.1002/mco2.70319 PMC12356995

[200] C. Abad , I. Pinal‐Fernandez , C. Guillou , et al., “IFNγ Causes Mitochondrial Dysfunction and Oxidative Stress in Myositis,” Nature Communications 15, no. 1 (2024): 5403. 10.1038/s41467-024-49460-1 PMC11208592 38926363

[201] N. Natarajan , J. Florentin , E. Johny , et al., “Aberrant Mitochondrial DNA Synthesis in Macrophages Exacerbates Inflammation and Atherosclerosis,” Nature Communications 15, no. 1 (2024): 7337. 10.1038/s41467-024-51780-1 PMC11347661 39187565

[202] Y. Guo , Y. You , F. F. Shang , et al., “INOS Aggravates Pressure Overload‐Induced Cardiac Dysfunction via Activation of the Cytosolic‐mtDNA‐Mediated cGAS‐STING Pathway,” Theranostics 13, no. 12 (2023): 4229–4246. 37554263 10.7150/thno.84049 PMC10405855

[203] X. Wang , H. Zhang , Y. Wang , et al., “DNA Sensing via the cGAS/STING Pathway Activates the Immunoproteasome and Adaptive T‐Cell Immunity,” EMBO Journal 42, no. 8 (2023): e110597. 36912165 10.15252/embj.2022110597 PMC10106989

[204] T. Lu , Z. Zhang , Z. Bi , et al., “TFAM Deficiency in Dendritic Cells Leads to Mitochondrial Dysfunction and Enhanced Antitumor Immunity Through cGAS‐STING Pathway,” Journal for ImmunoTherapy of Cancer 11, no. 3 (2023): e005430. 36858460 10.1136/jitc-2022-005430 PMC9980377

[205] S. E. Weinberg and N. S. Chandel , “Mitochondria Reactive Oxygen Species Signaling in Immune Responses,” Immunity 58, no. 8 (2025): 1904–1921. 40763730 10.1016/j.immuni.2025.07.012 PMC12371701

[206] L. Shang , X. Jiang , X. Zhao , et al., “Mitochondrial DNA‐Boosted Dendritic Cell‐Based Nanovaccination Triggers Antitumor Immunity in Lung and Pancreatic Cancers,” Cell Reports Medicine 5, no. 7 (2024): 101648. 38986624 10.1016/j.xcrm.2024.101648 PMC11293323

[207] M. P. Cabral‐Piccin , L. Papagno , X. Lahaye , et al., “Primary Role of Type I Interferons for the Induction of Functionally Optimal Antigen‐Specific Cd8(+) T Cells in HIV Infection,” EBioMedicine 91 (2023): 104557. 37058769 10.1016/j.ebiom.2023.104557 PMC10130611

[208] M. Borsa , A. V. Lechuga‐Vieco , A. H. Kayvanjoo , et al., “Autophagy‐Regulated Mitochondrial Inheritance Controls Early Cd8+ T Cell Fate Commitment,” Nature Cell Biology 28, no. 1 (2026): 66–81. 41419571 10.1038/s41556-025-01835-2 PMC12807857

[209] E. M. Steinert , B. Furtado Bruza , V. D. Danchine , et al., “Mitochondrial Respiration Is Necessary for Cd8+ T Cell Proliferation and Cell Fate,” Nature Immunology 26, no. 8 (2025): 1267–1274. 40670617 10.1038/s41590-025-02202-x PMC12307223

[210] X. Zhao , J. Zhang , C. Li , et al., “Mitochondrial Mechanisms in Treg Cell Regulation: Implications for Immunotherapy and Disease Treatment,” Mitochondrion 80 (2025): 101975. 39491776 10.1016/j.mito.2024.101975

[211] L. Alic , K. Dendinovic , and N. Papac‐Milicevic , “The Complement System in Lipid‐Mediated Pathologies,” Frontiers in Immunology 15 (2024): 1511886. 39635529 10.3389/fimmu.2024.1511886 PMC11614835

[212] A. Imbesi , A. Greco , M. Spagnolo , et al., “Targeting Inflammation After Acute Myocardial Infarction,” JACC 86, no. 15 (2025): 1146–1169. 41062230 10.1016/j.jacc.2025.07.064

[213] F. G. P. Welt , W. Batchelor , J. R. Spears , et al., “Reperfusion Injury in Patients With Acute Myocardial Infarction: JACC Scientific Statement,” Journal of the American College of Cardiology 83, no. 22 (2024): 2196–2213. 38811097 10.1016/j.jacc.2024.02.056

[214] G. A. Mensah , N. Arnold , S. D. Prabhu , et al., “Inflammation and Cardiovascular Disease: 2025 ACC Scientific Statement: A Report of the American College of Cardiology,” JACC 87, no. 11 (2026): 1381–1404. 10.1016/j.jacc.2025.08.047 41020749

[215] C. Liu , R. Wu , H. Yang , et al., “Immune Cell Dynamics and Their Role in Cardiac Injury: Mechanisms and Therapeutic Implications,” Biomedicine & Pharmacotherapy 192 (2025): 118608. 41016152 10.1016/j.biopha.2025.118608

[216] R. Dharmakumar , R. A. Kloner , M. Fishbein , et al., “Reperfused Myocardial Infarction: The Road to Ccs Classification of Acute Mi and Beyond,” JACC: Advances 4, no. 2 (2025): 101528. 40021272 10.1016/j.jacadv.2024.101528 PMC11905164

[217] S. Nesci and S. Rubattu , “Mitochondrial Permeability Transition Pore: The Cardiovascular Disease's Molecular Achilles Heel,” Biomedicines 13, no. 12 (2025): 3014. 41463026 10.3390/biomedicines13123014 PMC12731116

[218] S. Gastaldi , M. Giordano , F. Blua , et al., “Novel NLRP3 Inhibitor Inf195: Low Doses Provide Effective Protection Against Myocardial Ischemia/Reperfusion Injury,” Vascular Pharmacology 156 (2024): 107397. 38897555 10.1016/j.vph.2024.107397

[219] A. Ajoolabady , D. Pratico , L. Lin , et al., “Inflammation in Atherosclerosis: Pathophysiology and Mechanisms,” Cell Death & Disease 15, no. 11 (2024): 817. 39528464 10.1038/s41419-024-07166-8 PMC11555284

[220] J. Komuro , H. Hashimoto , T. Katsuki , et al., “Heart Failure‐Specific Cardiac Fibroblasts Contribute to Cardiac Dysfunction via the Myc–Cxcl1–Cxcr2 Axis,” Nature Cardiovascular Research 4, no. 9 (2025): 1135–1151. 10.1038/s44161-025-00698-y PMC12436193 40931092

[221] K. A. Walker , N. Basisty , D. M. Wilson 3rd , et al., “Connecting Aging Biology and Inflammation in the Omics Era,” Journal of Clinical Investigation 132, no. 14 (2022): e158448. 35838044 10.1172/JCI158448 PMC9282936

[222] M. A. Matter , F. Paneni , P. Libby , et al., “Inflammation in Acute Myocardial Infarction: The Good, the Bad and the Ugly,” European Heart Journal 45, no. 2 (2024): 89–103. 37587550 10.1093/eurheartj/ehad486 PMC10771378

[223] A. L. Koenig , I. Shchukina , J. Amrute , et al., “Single‐Cell Transcriptomics Reveals Cell‐Type‐Specific Diversification in Human Heart Failure,” Nature Cardiovascular Research 1, no. 3 (2022): 263–280. 10.1038/s44161-022-00028-6 PMC9364913 35959412

[224] S. Van Kesteren , L. Smeehuijzen , R. Stevenson , and J. Kroon , “Endothelial Cells Modulate Immune Cell Responses During Atherosclerosis,” Trends in Immunology (2026): S1471‐4906(26)00041‐00044, 10.1016/j.it.2026.03.001, online ahead of print.41925412

[225] P. Libby and O. Soehnlein , “Inflammation in Atherosclerosis: Lessons and Therapeutic Implications,” Immunity 58, no. 10 (2025): 2383–2401. 41045921 10.1016/j.immuni.2025.09.012

[226] M. Yalcinkaya , W. Liu , T. Xiao , et al., “Cholesterol Trafficking to the Er Leads to the Activation of Camkii/Jnk/NLRP3 and Promotes Atherosclerosis,” Journal of Lipid Research 65, no. 4 (2024): 100534. 38522750 10.1016/j.jlr.2024.100534 PMC11031842

[227] L. Zhuang , X. Zong , Q. Yang , et al., “Interleukin‐34‐Nf‐Κb Signaling Aggravates Myocardial Ischemic/Reperfusion Injury by Facilitating Macrophage Recruitment and Polarization,” eBioMedicine 95 (2023): 104744. 37556943 10.1016/j.ebiom.2023.104744 PMC10433018

[228] K. Jin , Z. Ma , X. Wang , et al., “The Role of Cardiac Macrophages in Inflammation and Fibrosis After Myocardial Ischemia‐Reperfusion,” Reviews in Cardiovascular Medicine 25, no. 11 (2024): 419. 39618853 10.31083/j.rcm2511419 PMC11607502

[229] G. Fragasso , D. Stolfo , M. S. Anker , et al., “The Crosstalk Between Immune Activation and Metabolism in Heart Failure. A Scientific Statement of the Heart Failure Association of the ESC,” European Journal of Heart Failure 27, no. 9 (2025): 1700–1719. 40521614 10.1002/ejhf.3703 PMC12502469

[230] J. M. Amrute , X. Luo , V. Penna , et al., “Targeting Immune‐Fibroblast Cell Communication in Heart Failure,” Nature 635, no. 8038 (2024): 423–433. 39443792 10.1038/s41586-024-08008-5 PMC12334188

[231] S. Van Linthout , I. Matz , A. González , et al., “Cardiac Fibroblasts in Myocardial Injury and Heart Failure,” European Heart Journal 47, no. 19 (2025): 2271–2285. 10.1093/eurheartj/ehaf902 PMC13183181 41288379

[232] A. E. Williams , L. S. Dunaway , Z. J. Juśkiewicz , et al., “Impact of Endothelial Diversity and Dysfunction on Cardiovascular Disease,” Comprehensive Physiology 15, no. 6 (2025): e70064. 41178013 10.1002/cph4.70064 PMC12580567

[233] T. Li , N. Wang , D. Yi , et al., “Ros‐Mediated Ferroptosis and Pyroptosis in Cardiomyocytes: An Update,” Life Sciences 370 (2025): 123565. 40113077 10.1016/j.lfs.2025.123565

[234] A. Rao , A. Gupta , V. Kain , et al., “Extrinsic and Intrinsic Modulators of Inflammation‐Resolution Signaling in Heart Failure,” American Journal of Physiology. Heart and Circulatory Physiology 325, no. 3 (2023): H433–H448. 37417877 10.1152/ajpheart.00276.2023 PMC10538986

[235] L. Guenin‐Mace , P. Konieczny , and S. Naik , “Immune‐Epithelial Cross Talk in Regeneration and Repair,” Annual Review of Immunology 41 (2023): 207–228. 10.1146/annurev-immunol-101721-062818 PMC10750769 36696569

[236] S. Schuermans , C. Kestens , and P. E. Marques , “Systemic Mechanisms of Necrotic Cell Debris Clearance,” Cell Death & Disease 15, no. 8 (2024): 557. 39090111 10.1038/s41419-024-06947-5 PMC11294570

[237] Y. Z. Lu , B. Nayer , S. K. Singh , et al., “Cgrp Sensory Neurons Promote Tissue Healing via Neutrophils and Macrophages,” Nature 628, no. 8008 (2024): 604–611. 38538784 10.1038/s41586-024-07237-y PMC11023938

[238] H. Mao , X. Zhao , and S.‐C. Sun , “Nf‐Κb in Inflammation and Cancer,” Cellular & Molecular Immunology 22, no. 8 (2025): 811–839. 40562870 10.1038/s41423-025-01310-w PMC12310982

[239] O. A. Peña and P. Martin , “Cellular and Molecular Mechanisms of Skin Wound Healing,” Nature Reviews Molecular Cell Biology 25, no. 8 (2024): 599–616. 38528155 10.1038/s41580-024-00715-1

[240] H. Liu , C. Zhen , J. Xie , et al., “Tfam Is an Autophagy Receptor That Limits Inflammation by Binding to Cytoplasmic Mitochondrial DNA,” Nature Cell Biology 26, no. 6 (2024): 878–891. 38783142 10.1038/s41556-024-01419-6

[241] F. S. Younesi , A. E. Miller , T. H. Barker , et al., “Fibroblast and Myofibroblast Activation in Normal Tissue Repair and Fibrosis,” Nature Reviews Molecular Cell Biology 25, no. 8 (2024): 617–638. 38589640 10.1038/s41580-024-00716-0

[242] Y. Huang , W. Jiang , and R. Zhou , “Damp Sensing and Sterile Inflammation: Intracellular, Intercellular and Inter‐Organ Pathways,” Nature Reviews Immunology 24, no. 10 (2024): 703–719. 10.1038/s41577-024-01027-3 38684933

[243] K. Yusri , S. Jose , K. S. Vermeulen , et al., “The Role of Nad+ Metabolism and Its Modulation of Mitochondria in Aging and Disease,” NPJ Metabolic Health and Disease 3, no. 1 (2025): 26. 40604314 10.1038/s44324-025-00067-0 PMC12177089

[244] D. Trinh , L. Al Halabi , H. Brar , et al., “The Role of Sirt3 in Homeostasis and Cellular Health,” Frontiers in Cellular Neuroscience 18 (2024): 1434459. 39157755 10.3389/fncel.2024.1434459 PMC11327144

[245] T. K. T. Smith , L. K. Townsend , W. J. Smiles , et al., “Ampk at the Interface of Nutrient Sensing, Metabolic Flux and Energy Homeostasis,” Nature Metabolism 8, no. 1 (2026): 27–51. 10.1038/s42255-025-01442-3 41526585

[246] A. C. Doran , “Inflammation Resolution: Implications for Atherosclerosis,” Circulation Research 130, no. 1 (2022): 130–148. 34995137 10.1161/CIRCRESAHA.121.319822 PMC8842990

[247] I. Hilgendorf , S. Frantz , and N. G. Frangogiannis , “Repair of the Infarcted Heart: Cellular Effectors, Molecular Mechanisms and Therapeutic Opportunities,” Circulation Research 134, no. 12 (2024): 1718–1751. 38843294 10.1161/CIRCRESAHA.124.323658 PMC11164543

[248] E. Bertero , T. A. Popoiu , and C. Maack , “Mitochondrial Calcium in Cardiac Ischemia/Reperfusion Injury and Cardioprotection,” Basic Research in Cardiology 119, no. 4 (2024): 569–585. 38890208 10.1007/s00395-024-01060-2 PMC11319510

[249] Q. Li , Y. Luo , H. Guo , et al., “Compound Danshen Tablets Ameliorate Myocardial Ischemia/Reperfusion Injury‐Induced Ventricular Remodeling by Regulating Autophagy via Ampk/Mtor Signaling Pathway,” Chinese Herbal Medicines 17, no. 3 (2025): 548–554. 40734915 10.1016/j.chmed.2024.03.003 PMC12301916

[250] C. Kim , H. Kim , W.‐S. Sim , et al., “Spatiotemporal Control of Neutrophil Fate to Tune Inflammation and Repair for Myocardial Infarction Therapy,” Nature Communications 15, no. 1 (2024): 8481. 10.1038/s41467-024-52812-6 PMC11445496 39353987

[251] K. M. Kindberg , K. Broch , G. Ø. Andersen , et al., “Neutrophil Extracellular Traps in St‐Segment Elevation Myocardial Infarction: Reduced by Tocilizumab and Associated With Infarct Size,” JACC: Advances 3, no. 9, pt. 1 (2024): 101193. 39247678 10.1016/j.jacadv.2024.101193 PMC11378880

[252] Y. Wan , J. Zhang , and Y. Yang , “The Role and Therapeutic Advances of Neutrophils in Acute Myocardial Infarction: From Traditional Chinese Medicine Modulation to Modern Therapeutic Strategies,” Chinese Medicine 20, no. 1 (2025): 213. 41345695 10.1186/s13020-025-01261-4 PMC12679801

[253] C. Yuan , B. Yu , L. Li , et al., “Sucnr 1 Promotes Atherosclerosis by Inducing Endoplasmic Reticulum Stress Mediated Er‐Mito Crosstalk,” International Immunopharmacology 143, no. pt. 3 (2024): 113510. 39486175 10.1016/j.intimp.2024.113510

[254] Z. Chen , X. Lai , J. Li , et al., “Brg1 Deficiency Promotes Cardiomyocyte Inflammation and Apoptosis by Activating the cGAS‐STING Signaling in Diabetic Cardiomyopathy,” Inflammation 48, no. 1 (2025): 299–315. 38867118 10.1007/s10753-024-02058-7 PMC11807080

[255] M. E. Pepin and R. M. Gupta , “The Role of Endothelial Cells in Atherosclerosis: Insights From Genetic Association Studies,” American Journal of Pathology 194, no. 4 (2024): 499–509. 37827214 10.1016/j.ajpath.2023.09.012 PMC10988759

[256] J. Ma , Y. Wang , W. Xu , et al., “Macrophage Pyroptosis in Atherosclerosis: Therapeutic Potential,” Acta Biochimica et Biophysica Sinica (Shanghai) 57, no. 6 (2025): 857–870. 10.3724/abbs.2025004 PMC12411746 39953798

[257] M. J. De Blasio and R. H. Ritchie , “Inflammatory Signalling in Diabetic Cardiomyopathy: Molecular Mechanisms and Potential Therapeutic Strategies,” Nature Reviews Cardiology (2026), 10.1038/s41569-026-01274-y, online ahead of print. 41862749

[258] L. Wang , S. Zhang , J. Han , et al., “Activation of Sting Pathway Contributed to Cisplatin‐Induced Cardiac Dysfunction via Promoting the Activation of Tnf‐Alpha‐Ap‐1 Signal Pathway,” Frontiers in Pharmacology 12 (2021): 711238. 34483919 10.3389/fphar.2021.711238 PMC8415915

[259] M. Bellemare , L. Bourcier , J. Iglesies‐Grau , et al., “Mechanisms of Diabetic Cardiomyopathy: Focus on Inflammation,” Diabetes, Obesity & Metabolism 27, no. 5 (2025): 2326–2338. 10.1111/dom.16242 PMC11964996 39930551

[260] L. Song , Q. Qiu , F. Ju , et al., “Mechanisms of Doxorubicin‐Induced Cardiac Inflammation and Fibrosis; Therapeutic Targets and Approaches,” Archives of Biochemistry and Biophysics 761 (2024): 110140. 39243924 10.1016/j.abb.2024.110140

[261] T. Ma , L. Wang , X. Yan , et al., “Targeted Anti‐Inflammatory Therapy in Cardiovascular Events: Challenges and Opportunities,” Journal of Clinical Hypertension (Greenwich) 27, no. 11 (2025): e70172. 10.1111/jch.70172 PMC12628085 41257435

[262] A. M. Parker , J. G. Lees , A. J. Murray , et al., “Precision Medicine: Therapeutically Targeting Mitochondrial Alterations in Heart Failure,” JACC: Basic to Translational Science 10, no. 9 (2025): 101345. 40845481 10.1016/j.jacbts.2025.101345 PMC12539467

[263] X. Liu , W. Zhang , X. Miao , et al., “Natural Metabolites Used in Traditional Chinese Medicine for Cardiovascular Diseases: Pharmacological Mechanisms, Evidence, and Future Directions,” Frontiers in Pharmacology 16 (2025): 1656751. 41293245 10.3389/fphar.2025.1656751 PMC12641511

[264] W. L. Hong , H. Huang , X. Zeng , et al., “Targeting Mitochondrial Quality Control: New Therapeutic Strategies for Major Diseases,” Military Medical Research 11, no. 1 (2024): 59. 39164792 10.1186/s40779-024-00556-1 PMC11337860

[265] L. Ai , J. De Freitas Germano , C. Huang , et al., “Enhanced Parkin‐Mediated Mitophagy Mitigates Adverse Left Ventricular Remodelling After Myocardial Infarction: Role of Pr‐364,” European Heart Journal 46, no. 4 (2025): 380–393. 39601359 10.1093/eurheartj/ehae782 PMC11745530

[266] P. A. Glogowski , F. Fogacci , C. Algieri , et al., “Reprogramming the Mitochondrion in Atherosclerosis: Targets for Vascular Protection,” Antioxidants (Basel) 14, no. 12 (2025): 1462. 41462662 10.3390/antiox14121462 PMC12729673

[267] H. Zhang , S. Xie , and W. Deng , “Mitophagy in Doxorubicin‐Induced Cardiotoxicity: Insights Into Molecular Biology and Novel Therapeutic Strategies,” Biomolecules 14, no. 12 (2024): 1614. 39766321 10.3390/biom14121614 PMC11674137

[268] H. Zheng , H. Zhu , X. Liu , et al., “Mitophagy in Diabetic Cardiomyopathy: Roles and Mechanisms,” Frontiers in Cell and Developmental Biology 9 (2021): 750382. 34646830 10.3389/fcell.2021.750382 PMC8503602

[269] Q. Zhang , L. Shen , H. Ruan , et al., “cGAS‐STING Signaling in Cardiovascular Diseases,” Frontiers in Immunology 15 (2024): 1402817. 38803502 10.3389/fimmu.2024.1402817 PMC11128581

[270] M. K. Woolls , C. M. Elliott , H. M. Ivester , et al., “Mystery Machine: The Complex Roles of NLRX1 in Viral Infection,” Frontiers in Immunology 16 (2025): 1581313. 40356929 10.3389/fimmu.2025.1581313 PMC12066249

[271] G. V. Halade , M. Bäck , and V. Kain , “Inflammation‐Resolution Signalling in Cardiac Repair, Remodelling, and Heart Failure,” European Heart Journal 5, no. 6 (2025): oeaf157. 10.1093/ehjopen/oeaf157 PMC12728415 41451243

[272] A. Li , X. Wang , R. Yang , et al., “Therapeutic Potential and Mechanisms of Traditional Chinese Medicine in Regulating Energy Metabolism Imbalance in Heart Failure,” Chinese Herbal Medicines 17, no. 4 (2025): 685–702. 41399793 10.1016/j.chmed.2025.07.002 PMC12702449

[273] Y. Liu , L. Li , Z. Wang , et al., “Myocardial Ischemia‐Reperfusion Injury; Molecular Mechanisms and Prevention,” Microvascular Research 149 (2023): 104565. 37307911 10.1016/j.mvr.2023.104565

[274] Q. Lu , S. Luo , C. Guan , et al., “Research Progress of Regulating Intestinal Flora by Traditional Chinese Medicine in Treating Coronary Heart Disease,” Chinese Herbal Medicines 17, no. 3 (2025): 464–472. 40734911 10.1016/j.chmed.2025.04.007 PMC12301970

[275] M. Wang , M. Yan , L. Tan , et al., “Non‐Coding RNAs: Targets for Chinese Herbal Medicine in Treating Myocardial Fibrosis,” Frontiers in Pharmacology 15 (2024): 1337623. 38476331 10.3389/fphar.2024.1337623 PMC10928947

[276] J. Zhou , Z. Wang , Y. He , et al., “Qiliqiangxin Reduced Cardiomyocytes Apotosis and Improved Heart Function in Infarcted Heart Through Pink1/Parkin‐Mediated Mitochondrial Autophagy,” BMC Complementary Medicine and Therapies 20, no. 1 (2020): 203. 32615967 10.1186/s12906-020-02992-7 PMC7330946

[277] J. Hu , L. Zhang , F. Fu , et al., “Cardioprotective Effect of Ginsenoside Rb1 via Regulating Metabolomics Profiling and AMP‐Activated Protein Kinase‐Dependent Mitophagy,” Journal of Ginseng Research 46, no. 2 (2022): 255–265. 35509816 10.1016/j.jgr.2021.06.011 PMC9058834

[278] W. Sijia , L. Yingying , W. Haonan , et al., “Protective Effects of Optimised Tanyu Tongzhi Decoction Against Myocardial Ischemia‐Reperfusion No‐Reflow in Rats via Inhibiting Nets Generation,” Chinese Journal of Experimental Traditional Medical Formulae 32, no. 8 (2025): 99–107.

[279] J. K. Li , Z. P. Song , and X. Z. Hou , “Scutellarin Ameliorates Ischemia/Reperfusion Injury‑Induced Cardiomyocyte Apoptosis and Cardiac Dysfunction via Inhibition of the cGAS‑STING Pathway,” Experimental and Therapeutic Medicine 25, no. 4 (2023): 155. 36911381 10.3892/etm.2023.11854 PMC9996299

[280] Z. Kong , P. Sun , Y. Lu , et al., “Yi Mai Granule Improve Energy Supply of Endothelial Cells in Atherosclerosis via miRNA‐125a‐5p Regulating Mitochondrial Autophagy Through Pink1‐Mfn2‐Parkin Pathway,” Journal of Ethnopharmacology 319, no. pt. 1 (2024): 117114. 37678420 10.1016/j.jep.2023.117114

[281] X. Wang , M. Zhang , C. Mao , et al., “Icariin Alleviates Ferroptosis‐Related Atherosclerosis by Promoting Autophagy in Xo‐LDL‐Induced Vascular Endothelial Cell Injury and Atherosclerotic Mice,” Phytotherapy Research 37, no. 9 (2023): 3951–3963. 37344941 10.1002/ptr.7854

[282] C. Huang , L. Huang , Q. Huang , et al., “Mitophagy Disorder Mediates Cardiac Deterioration Induced by Severe Hypoglycemia in Diabetic Mice,” Molecular and Cellular Endocrinology 575 (2023): 111994. 37330037 10.1016/j.mce.2023.111994

[283] Z. Chen , S. Li , M. Liu , et al., “Nicorandil Alleviates Cardiac Microvascular Ferroptosis in Diabetic Cardiomyopathy: Role of the Mitochondria‐Localized Ampk‐Parkin‐Acsl4 Signaling Pathway,” Pharmacological Research 200 (2024): 107057. 38218357 10.1016/j.phrs.2024.107057

[284] L. Zhu , Z. Liu , J. Liu , et al., “Ncoa4 Linked to Endothelial Cell Ferritinophagy and Ferroptosis: A Key Regulator Aggravate Aortic Endothelial Inflammation and Atherosclerosis,” Redox Biology 79 (2025): 103465. 39700692 10.1016/j.redox.2024.103465 PMC11729014

[285] X. Jiangying , W. Yanping , and Z. Yilin , “Effects of Baicalin on Inflammatory Responses in Diabetic Cardiomyopathy Rats by Regulating cGAS‐STING Signaling Pathway,” New Chinese Medicine 57, no. 04 (2025): 165–171.

[286] W. Li , Z. Huang , Y. Luo , et al., “Tetrandrine Alleviates Atherosclerosis via Inhibition of STING‐TBK1 Pathway and Inflammation in Macrophages,” International Immunopharmacology 119 (2023): 110139. 37099944 10.1016/j.intimp.2023.110139

[287] L. Lu , Y. Shao , X. Xiong , et al., “Irisin Improves Diabetic Cardiomyopathy‐Induced Cardiac Remodeling by Regulating GSDMD‐Mediated Pyroptosis Through Mitol/STING Signaling,” Biomedicine & Pharmacotherapy 171 (2024): 116007. 38171238 10.1016/j.biopha.2023.116007

[288] S. Gong , Y. Sui , M. Xiao , et al., “Canagliflozin Mediates Mitophagy Through the Ampk/Pink1/Parkin Pathway to Alleviate Iso‐Induced Cardiac Remodeling,” Journal of Cardiovascular Pharmacology 84, no. 5 (2024): 496–505. 39150485 10.1097/FJC.0000000000001625

[289] L. Li , S. Nie , B. Wang , et al., “Shenfuyixin Granules Enhance Mitochondrial Autophagy After Myocardial Infarction by Regulating Protein Deacetylation via the Sirt3/Foxo1 Signaling Axis,” Phytomedicine 139 (2025): 156503. 39986233 10.1016/j.phymed.2025.156503

[290] Z. Chen , M. Zhang , Q. Xu , et al., “Huangqi‐Danshen Decoction Improves Heart Failure by Regulating Pericardial Adipose Tissue Derived Extracellular Vesicular Mir‐27a‐3p to Activate Ampkalpha2 Mediated Mitophagy,” Phytomedicine 135 (2024): 156187. 39488874 10.1016/j.phymed.2024.156187

[291] Y. Yaxin , S. Yan , C. Yu , et al., “Effect of Modified Yiwei Shengyang Decoction on Myocardial Fibrosis in Heart Failure With Preserved Ejection Fraction Mice,” Shanghai Journal of Traditional Chinese Medicine 59, no. 06 (2025): 22–27+35.

[292] T. Siqin , G. Bing , L. Liang , et al., “Study on the Regulatory Effect of Xinkang Granules on Inflammatory Factors in Rats With Chronic Heart Failure Based on the cGAS/STING Signaling Pathway,” Traditional Chinese Drug Research & Clinical Pharmacology 35, no. 05 (2024): 674–680.

[293] Y. Zhi , W. Jun , A. Sheng , et al., “Qingdu Wenxin Formula Mitigates Doxorubicin‐Induced Cardiotoxicity via Inhibition of cGAS/STING/Nf‐Κb Pathway‐Mediated Inflammation,” China Journal of Chinese Materia Medica 50, no. 20 (2025): 5820–5829. 41508213 10.19540/j.cnki.cjcmm.20250609.703

[294] Y. Yao , L. Lin , W. Tang , et al., “Pretreatment With Geniposide Mitigates Myocardial Ischemia/Reperfusion Injury by Modulating Inflammatory Response Through Tlr4/Nf‐Κb Pathway,” European Journal of Histochemistry 67, no. 3 (2023): 3742. 37682077 10.4081/ejh.2023.3742 PMC10518652

[295] S. Yanan , L. Bohan , S. Shuaifeng , et al., “Inhibition of Mogroside Iiie on Isoproterenol‐Induced Myocardial Fibrosis Through the Tlr4/Myd88/Nf‐Κb Signaling Pathway,” Iranian Journal of Basic Medical Sciences 26, no. 1 (2023): 114–120. 36594066 10.22038/IJBMS.2022.67908.14848 PMC9790049

[296] L. Zhang , S. Zhao , and Y. Wang , “Diannexin Alleviates Myocardial Ischemia‐Reperfusion Injury by Orchestrating Cardiomyocyte Oxidative Damage, Macrophage Polarization and Fibrotic Process by Tlr4‐Nf‐Kb‐Mediated Inactivation of NLRP3 Inflammasome,” International Immunopharmacology 130 (2024): 111668. 38417368 10.1016/j.intimp.2024.111668

[297] S. Yin , K. Han , D. Wu , et al., “Tilianin Suppresses NLRP3 Inflammasome Activation in Myocardial Ischemia/Reperfusion Injury via Inhibition of Tlr4/Nf‐Κb and Nek7/NLRP3,” Frontiers in Pharmacology 15 (2024): 1423053. 39508038 10.3389/fphar.2024.1423053 PMC11538317

[298] B. Ye , X. Cai , X. Liang , et al., “Emodin Suppresses NLRP3/Gsdmd‐Induced Inflammation via the Tlr4/Myd88/Nf‐Κb Signaling Pathway in Atherosclerosis,” Cardiovascular Drugs and Therapy 39, no. 6 (2025): 1289–1301. 39715879 10.1007/s10557-024-07659-w

[299] M. I. H. Sagor , Q. Wang , J. Wang , et al., “Cyclodextrin Attenuates Atherosclerosis by Diminishing Gasdermin D (GSDMD)‐Mediated Pyroptosis,” Scientific Reports 15, no. 1 (2025): 21605. 40594437 10.1038/s41598-025-04889-2 PMC12218160

[300] K. Tian , L. Song , L. Liu , et al., “Rutin Protects Myocardial Ischemia‐Reperfusion Injury via the Nf‐Κb/NLRP3/Pyroptosis Pathway,” ACS Omega 10, no. 21 (2025): 21777–21785. 40488073 10.1021/acsomega.5c01408 PMC12138673

[301] Y. Hu , S. Zhang , H. Lou , et al., “Aloe‐Emodin Derivative, an Anthraquinone Compound, Attenuates Pyroptosis by Targeting NLRP3 Inflammasome in Diabetic Cardiomyopathy,” Pharmaceuticals (Basel) 16, no. 9 (2023): 1275. 37765083 10.3390/ph16091275 PMC10536457

[302] S. Ma , J. Feng , X. Lin , et al., “Nicotinamide Riboside Alleviates Cardiac Dysfunction and Remodeling in Pressure Overload Cardiac Hypertrophy,” Oxidative Medicine and Cellular Longevity 2021 (2021): 5546867. 34567409 10.1155/2021/5546867 PMC8463245

[303] C. Zhong , Y. Xie , H. Wang , et al., “Berberine Inhibits NLRP3 Inflammasome Activation by Regulating Mtor/Mtros Axis to Alleviate Diabetic Cardiomyopathy,” European Journal of Pharmacology 964 (2024): 176253. 38096968 10.1016/j.ejphar.2023.176253

[304] J. Fu , H. Xu , F. Wu , et al., “Empagliflozin Inhibits Macrophage Inflammation Through Ampk Signaling Pathway and Plays an Anti‐Atherosclerosis Role,” International Journal of Cardiology 367 (2022): 56–62. 35931206 10.1016/j.ijcard.2022.07.048

[305] F. Peng , M. Liao , W. Jin , et al., “2‐Apqc, a Small‐Molecule Activator of Sirtuin‐3 (Sirt3), Alleviates Myocardial Hypertrophy and Fibrosis by Regulating Mitochondrial Homeostasis,” Signal Transduction and Targeted Therapy 9, no. 1 (2024): 133. 38744811 10.1038/s41392-024-01816-1 PMC11094072

[306] P. Kleinbongard , C. G. Arriola , L. Badimon , et al., “The Improving Preclinical Assessment of Cardioprotective Therapies (Impact): Multicenter Pig Study on the Effect of Ischemic Preconditioning,” Basic Research in Cardiology 119, no. 6 (2024): 893–909. 39422732 10.1007/s00395-024-01083-9 PMC11628588

[307] F. P. Kreutzer , A. Meinecke , K. Schmidt , et al., “Alternative Strategies in Cardiac Preclinical Research and New Clinical Trial Formats,” Cardiovascular Research 118, no. 3 (2021): 746–762. 10.1093/cvr/cvab075 PMC7989574 33693475

[308] D. M. Morariu‐Briciu , A. R. Jîjie , S. L. Bolintineanu , et al., “Medicinal Plants and Phytochemicals in Cardioprotection‐Mechanistic Pathways and Translational Roadmap,” Life (Basel) 16, no. 1 (2026): 175. 41598329 10.3390/life16010175 PMC12843228

[309] H. K. Kim , S. J. Kim , W. J. Gil , et al., “Exploring the Therapeutic Potential of Phytochemicals: Challenges and Strategies for Clinical Translation,” Phytomedicine 145 (2025): 157090. 40716124 10.1016/j.phymed.2025.157090

[310] S. Choi , I. G. Ju , M. Lee , et al., “Naeso‐San, a Traditional Herbal Formula, Attenuates Hcl/Ethanol‐Induced Gastric Injury via Mapk and Nf‐Κb Pathway Modulation in Mice,” Frontiers in Pharmacology 16 (2025): 1672854. 41601984 10.3389/fphar.2025.1672854 PMC12833003

[311] P. M. Ridker , B. M. Everett , T. Thuren , et al., “Antiinflammatory Therapy With Canakinumab for Atherosclerotic Disease,” New England Journal of Medicine 377, no. 12 (2017): 1119–1131. 28845751 10.1056/NEJMoa1707914

[312] A. Abbate , M. C. Kontos , J. D. Grizzard , et al., “Interleukin‐1 Blockade With Anakinra to Prevent Adverse Cardiac Remodeling After Acute Myocardial Infarction (Virginia Commonwealth University Anakinra Remodeling Trial [Vcu‐Art] Pilot Study),” American Journal of Cardiology 105, no. 10 (2010): 1371–1377.e1. 20451681 10.1016/j.amjcard.2009.12.059

[313] O. Kleveland , G. Kunszt , M. Bratlie , et al., “Effect of a Single Dose of the Interleukin‐6 Receptor Antagonist Tocilizumab on Inflammation and Troponin T Release in Patients With Non‐St‐Elevation Myocardial Infarction: A Double‐Blind, Randomized, Placebo‐Controlled Phase 2 Trial,” European Heart Journal 37, no. 30 (2016): 2406–2413. 27161611 10.1093/eurheartj/ehw171

[314] P. M. Ridker , M. Devalaraja , F. M. M. Baeres , et al., “IL‐6 Inhibition With Ziltivekimab in Patients at High Atherosclerotic Risk (Rescue): A Double‐Blind, Randomised, Placebo‐Controlled, Phase 2 Trial,” Lancet 397, no. 10289 (2021): 2060–2069. 34015342 10.1016/S0140-6736(21)00520-1

[315] P. M. Ridker , B. M. Everett , A. Pradhan , et al., “Low‐Dose Methotrexate for the Prevention of Atherosclerotic Events,” New England Journal of Medicine 380, no. 8 (2019): 752–762. 30415610 10.1056/NEJMoa1809798 PMC6587584

[316] N. Bouabdallaoui , J. C. Tardif , D. D. Waters , et al., “Time‐to‐Treatment Initiation of Colchicine and Cardiovascular Outcomes After Myocardial Infarction in the Colchicine Cardiovascular Outcomes Trial (Colcot),” European Heart Journal 41, no. 42 (2020): 4092–4099. 32860034 10.1093/eurheartj/ehaa659 PMC7700755

[317] M. A. D'entremont , S. F. Lee , R. Mian , et al., “Design and Rationale of the Clear Synergy (Oasis 9) Trial: A 2×2 Factorial Randomized Controlled Trial of Colchicine Versus Placebo and Spironolactone Vs Placebo in Patients With Myocardial Infarction,” American Heart Journal 275 (2024): 173–182. 38936755 10.1016/j.ahj.2024.06.007

[318] M. L. O'donoghue , R. Glaser , M. A. Cavender , et al., “Effect of Losmapimod on Cardiovascular Outcomes in Patients Hospitalized With Acute Myocardial Infarction: A Randomized Clinical Trial,” JAMA 315, no. 15 (2016): 1591–1599. 27043082 10.1001/jama.2016.3609

[319] G. F. Wohlford , B. W. Van Tassell , H. E. Billingsley , et al., “Phase 1b, Randomized, Double‐Blinded, Dose Escalation, Single‐Center, Repeat Dose Safety and Pharmacodynamics Study of the Oral NLRP3 Inhibitor Dapansutrile in Subjects With Nyha II–III Systolic Heart Failure,” Journal of Cardiovascular Pharmacology 77, no. 1 (2021): 49–60. 10.1097/FJC.0000000000000931 PMC7774821 33235030

[320] J. C. Tardif , J. F. Tanguay , S. R. Wright , et al., “Effects of the P‐Selectin Antagonist Inclacumab on Myocardial Damage After Percutaneous Coronary Intervention for Non‐St‐Segment Elevation Myocardial Infarction: Results of the Select‐Acs Trial,” Journal of the American College of Cardiology 61, no. 20 (2013): 2048–2055. 23500230 10.1016/j.jacc.2013.03.003

[321] X. Zhao , T. Williamson , Y. Gong , et al., “Immunomodulatory Therapy for Ischemic Heart Disease,” Circulation 150, no. 13 (2024): 1050–1058. 39325497 10.1161/CIRCULATIONAHA.124.070368 PMC11521113

[322] O. A. Smiseth , O. Rider , M. Cvijic , et al., “Myocardial Strain Imaging: Theory, Current Practice, and the Future,” JACC Cardiovasc Imaging 18, no. 3 (2025): 340–381. 39269417 10.1016/j.jcmg.2024.07.011

[323] M. C. Gissler , P. Antiochos , Y. Ge , et al., “Cardiac Magnetic Resonance Evaluation of Lv Remodeling Post‐Myocardial Infarction: Prognosis, Monitoring and Trial Endpoints,” JACC: Cardiovascular Imaging 17, no. 11 (2024): 1366–1380. 38819335 10.1016/j.jcmg.2024.03.012

[324] H. Morgan , K. Little , S. Dutta , et al., “Cell‐Free Nucleic Acids in Cardiovascular Disease: From Biomarkers to Mechanistic Drivers and Therapeutic Opportunities,” Cells 15, no. 1 (2026): 33. 10.3390/cells15010033 PMC12786228 41511317

[325] D. Kabelitz , M. Zarobkiewicz , M. Heib , et al., “Signal Strength of Sting Activation Determines Cytokine Plasticity and Cell Death in Human Monocytes,” Scientific Reports 12, no. 1 (2022): 17827. 36280676 10.1038/s41598-022-20519-7 PMC9590392

[326] A. Aimo , J. Butler , L. De Michieli , et al., “Biomarkers for Cardiovascular Drug Development: Jacc State‐of‐the‐Art Review,” Journal of the American College of Cardiology 87, no. 15 (2026): 1950–1972. 41670555 10.1016/j.jacc.2025.12.072

[327] T. G. Osório , E. Pavesi , K. A. El‐Ardat , et al., “Single‐Cell Multi‐Omics for Precision Cardiovascular and Longevity Medicine: From Methods to Clinical Translation,” Frontiers in Aging 6 (2025): 1656727. 41393100 10.3389/fragi.2025.1656727 PMC12696164

[328] A. Mengozzi , S. Armenia , N. De Biase , et al., “Circulating Mitochondrial DNA Signature in Cardiometabolic Patients,” Cardiovascular Diabetology 24, no. 1 (2025): 106. 40045401 10.1186/s12933-025-02656-1 PMC11884014

[329] M. A. Psotka , W. T. Abraham , M. Fiuzat , et al., “Functional and Symptomatic Clinical Trial Endpoints: The Hfc‐Arc Scientific Expert Panel,” JACC Heart Failure 10, no. 12 (2022): 889–901. 36456063 10.1016/j.jchf.2022.09.012

[330] M. G. Rabbat , R. Y. Kwong , J. F. Heitner , et al., “The Future of Cardiac Magnetic Resonance Clinical Trials,” JACC: Cardiovascular Imaging 15, no. 12 (2022): 2127–2138. 34922874 10.1016/j.jcmg.2021.07.029

[331] A. Das , C. Kelly , I. Teh , et al., “Pathophysiology of Lv Remodeling Following STEMI: A Longitudinal Diffusion Tensor CMR Study,” JACC: Cardiovascular Imaging 16, no. 2 (2023): 159–171. 36412993 10.1016/j.jcmg.2022.04.002 PMC9902278

[332] S. Cederström , T. Jernberg , A. Samnegård , et al., “Inflammatory Biomarkers and Long‐Term Outcome in Young Patients Three Months After a First Myocardial Infarction,” Cytokine 182 (2024): 156696. 39059290 10.1016/j.cyto.2024.156696

[333] J. Butler , K. Hammonds , K. M. Talha , et al., “Incident Heart Failure and Recurrent Coronary Events Following Acute Myocardial Infarction,” European Heart Journal 46, no. 16 (2025): 1540–1550. 39874177 10.1093/eurheartj/ehae885 PMC12011519

[334] V. Rizza , L. Tondi , A. M. Patti , et al., “Diabetic Cardiomyopathy: Pathophysiology, Imaging Assessment and Therapeutical Strategies,” International Journal of Cardiology: Cardiovascular Risk and Prevention 23 (2024): 200338. 39734497 10.1016/j.ijcrp.2024.200338 PMC11681223

[335] K. Barnard‐Kelly , T. Battelino , F. C. Brosius , et al., “Defining Patient‐Reported Outcomes in Diabetes, Obesity, Cardiovascular Disease, and Chronic Kidney Disease for Clinical Practice Guidelines—Perspectives of the Taskforce of the Guideline Workshop,” Cardiovascular Diabetology 24, no. 1 (2025): 68. 39920737 10.1186/s12933-024-02550-2 PMC11806799

[336] J. Butler , C. E. Hamo , J. E. Udelson , et al., “Reassessing Phase II Heart Failure Clinical Trials: Consensus Recommendations,” Circulation: Heart Failure 10, no. 4 (2017): e003800. 28356300 10.1161/CIRCHEARTFAILURE.116.003800 PMC5400283

[337] C. Tiller , M. Reindl , M. Holzknecht , et al., “Association of Intramyocardial Hemorrhage With Inflammatory Biomarkers in Patients With ST‐Segment Elevation Myocardial Infarction,” JACC: Advances 4, no. 4 (2025): 101647. 40080922 10.1016/j.jacadv.2025.101647 PMC11953969

[338] S. Yum , M. Li , Y. Fang , et al., “TBK1 Recruitment to Sting Activates Both Irf3 and Nf‐Κb That Mediate Immune Defense Against Tumors and Viral Infections,” PNAS 118, no. 14 (2021): e2100225118. 33785602 10.1073/pnas.2100225118 PMC8040795

[339] A. Reyahi , M. Studahl , M. K. Skouboe , et al., “An Ikbke Variant Conferring Functional Cgas/Sting Pathway Deficiency and Susceptibility to Recurrent Hsv‐2 Meningitis,” JCI Insight 8, no. 21 (2023): e173066. 37937644 10.1172/jci.insight.173066 PMC10721272

[340] K. Paulsen , R. Chan , L. Gay , et al., “Kshv miRNAs Target Sting to Evade Innate Immunity and Facilitate Kshv Lytic Reactivation From Latency,” Cell Reports 44, no. 6 (2025): 115741. 40413741 10.1016/j.celrep.2025.115741 PMC12713167

[341] G. E. Fragoulis , E. Nikiphorou , M. Dey , et al., “2022 Eular Recommendations for Screening and Prophylaxis of Chronic and Opportunistic Infections in Adults With Autoimmune Inflammatory Rheumatic Diseases,” Annals of the Rheumatic Diseases 82, no. 6 (2023): 742–753. 36328476 10.1136/ard-2022-223335

[342] G. V. Papatheodoridis , V. Lekakis , T. Voulgaris , et al., “Hepatitis B Virus Reactivation Associated With New Classes of Immunosuppressants and Immunomodulators: A Systematic Review, Meta‐Analysis, and Expert Opinion,” Journal of Hepatology 77, no. 6 (2022): 1670–1689. 35850281 10.1016/j.jhep.2022.07.003

[343] Y. Liu , Y. Wang , Y. Bi , et al., “Emerging Role of Mitophagy in Heart Failure: From Molecular Mechanism to Targeted Therapy,” Cell Cycle 22, no. 8 (2023): 906–918. 36658777 10.1080/15384101.2023.2167949 PMC10054314

[344] L. Lu , Y. Shao , N. Wang , et al., “Follistatin‐Like Protein 1 Attenuates Doxorubicin‐Induced Cardiomyopathy by Inhibiting Msrb2‐Mediated Mitophagy,” Molecular and Cellular Biochemistry 479, no. 7 (2024): 1817–1831. 38696001 10.1007/s11010-024-04955-9

