# Supplementary material for: The Role of Inflammation and Immunity in Cardiovascular Disease: Molecular Mechanisms and Therapeutic Targets
Source: MedComm (2020). 2026 Jul 19;7(8):e70878. doi: 10.1002/mco2.70878 (PMC13382382; doi:10.1002/mco2.70878)
Supplement: Supplementary file 1 — Supporting Information File: mco270878‐sup‐0001‐SuppMat.docx [file MCO2-7-e70878-s001.docx]

**Supplementary Materials:**

**The role of inflammation and immunity in cardiovascular disease: molecular mechanisms and therapeutic targets**

**Jiaxiang Rong ^a, 1^, Zhen Wang ^a, 1^,** **Xiaoxiao Lin** **^a^,** **Ziwen Lei ^a^, Qianqian Huang ^a^,**

**Hang Liu ^b^,** **Fei Luan ^a,^ *, Junbo Zou** **^a,^ *, and Yajun Shi ^a,^ ***

^a^ Shaanxi Province Key Laboratory of New Drugs and Chinese Medicine Foundation Research, School of Pharmacy, Shaanxi University of Chinese Medicine, Xi’an 712046, Shaanxi, China;

^b^ Department of Pharmacy, Affiliated Hospital of Shaanxi University of Chinese Medicine, Xianyang 712000, P.R. China;

* **Corresponding authors at**: Shaanxi Province Key Laboratory of New Drugs and Chinese Medicine Foundation Research, School of Pharmacy, Shaanxi University of Chinese Medicine, No. 1, Shiji Avenue, Xi Xian New District, Xi’an City, 712046, Shaanxi, China; E-mail addresses: luanfeiren@163.com (**F. Luan**); 2051078@sntcm.edu.cn (**J. Zou**); 2051004@sntcm.edu.cn (**Y. Shi**); Tel./fax: +86-29-3818-5175.

**^1^ These authors contributed equally to this work.**

**Literature search and scope of this review:**

This review used a structured narrative search to identify studies related to inflammation and immunity in cardiovascular diseases. Literature published up to April 2026 was retrieved primarily from PubMed (https://pubmed.ncbi.nlm.nih.gov), Web of Science (https://www.webofscience.com/), China National Knowledge Infrastructure (https://www.cnki.net/index/), Scopus (https://www.elsevier.com/products/scopus), and ClinicalTrials.gov (https://clinicaltrials.gov). Reference lists of relevant original articles and reviews were also manually screened to identify additional studies of interest.

The main search terms included combinations of “cardiovascular disease”, “inflammation”, “immunity”, “mitophagy”, “mitochondrial DNA”, “cGAS-STING”, “TLR”, “NLRP3”, “inflammasome”, “immunothrombosis”, “adaptive immunity”, “immune metabolism”, “oxidative stress”, and related terms. Priority was given to studies with clear mechanistic relevance to cardiovascular inflammatory signaling, representative preclinical investigations, and clinical or translational studies involving inflammatory modulation in cardiovascular disease. Seminal earlier studies were also included when necessary to provide conceptual or mechanistic context.

The scope of this review encompasses studies addressing the immune landscape of cardiovascular diseases, core inflammatory signaling networks, mitochondrial danger signaling, mitophagy-related regulation, nucleic acid sensing pathways, immunometabolic control, and network-guided therapeutic strategies. Both experimental and clinical studies published in English and Chinese were considered. Studies with limited relevance to the present thematic focus, substantial overlap in mechanistic content, or insufficient direct connection to cardiovascular inflammation were not discussed in detail. As a narrative review, this article aims to provide an integrated and mechanism-oriented synthesis rather than a formal systematic review or meta-analysis.
